# Computational Analysis and Mapping of Novel Open Reading Frames in Influenza A Viruses

**DOI:** 10.1371/journal.pone.0115016

**Published:** 2014-12-15

**Authors:** Yu-Nong Gong, Guang-Wu Chen, Chi-Jene Chen, Rei-Lin Kuo, Shin-Ru Shih

**Affiliations:** 1 Department of Computer Science and Information Engineering, School of Electrical and Computer Engineering, College of Engineering, Chang Gung University, Taoyuan, Taiwan; 2 Research Center for Emerging Viral Infections, College of Medicine, Chang Gung University, Taoyuan, Taiwan; 3 Department of Medical Biotechnology and Laboratory Sciences, College of Medicine, Chang Gung University, Taoyuan, Taiwan; The University of Hong Kong, Hong Kong

## Abstract

The influenza A virus contains 8 segmented genomic RNAs and was considered to encode 10 viral proteins until investigators identified the 11^th^ viral protein, PB1-F2, which uses an alternative reading frame of the PB1 gene. The recently identified PB1-N40, PA-N155 and PA-N182 influenza A proteins have shown the potential for using a leaking ribosomal scanning mechanism to generate novel open reading frames (ORFs). These novel ORFs provide examples of the manner in which the influenza A virus expands its coding capacity by using overlapping reading frames. In this study, we performed a computational search, based on a ribosome scanning mechanism, on all influenza A coding sequences to identify possible forward-reading ORFs that could be translated into novel viral proteins. We specified that the translated products had a prevalence ≥5% to eliminate sporadic ORFs. A total of 1,982 ORFs were thus identified and presented in terms of their locations, lengths and Kozak sequence strengths. We further provided an abridged list of ORFs by requiring every candidate an upstream start codon (within the upstream third of the primary transcript), a strong Kozak consensus sequence and high prevalence (≥95% and ≥50% for in-frame and alternative-frame ORFs, respectively). The PB1-F2, PB1-N40, PA-N155 and PA-N182 proteins all fulfilled our filtering criteria. Subject to these three stringent settings, we additionally named 16 novel ORFs for all influenza A genomes except for HA and NA, for which 43 HA and 11 NA ORFs from their respective subtypes were also recognized.

## Introduction

In molecular biology, an open reading frame (ORF) is a nucleic acid sequence that does not contain a stop codon in a given reading frame, and can thus potentially be translated into a functional protein product. Ribosomal scanning is extensively used to detect ORFs from the 5′ end of the capped mRNA, initiating translation at the first identified AUG for the majority of proteins [1]–[4]. Other mechanisms exist that do not apply the first-AUG rule for generating proteins include leaky ribosomal scanning for betaA3/A1-crystallin [5], and reinitiation for the human T-call leukemia virus type l [6].

The influenza A virus is a human and animal pathogen that poses a threat to global public health, such as the H1N1 Spanish flu pandemic in 1918, the avian influenza A (H5N1) virus, which has caused human disease since 1997 [7], the pandemic influenza H1N1 (H1N1pdm or new H1N1) virus in 2009 [8], and the avian influenza A (H7N9) virus in 2013 [9]. The influenza A virus contains 8 segmented genomic RNAs and was considered to encode 10 viral proteins (PB2, PB1, PA, HA, NP, NA, M1, M2, NS1 and NS2) until investigators identified the 11^th^ influenza viral protein, PB1-F2, in 2001 [10]. As the name suggests, PB1-F2 is translated from a frame-2 ORF in PB1 transcript that was originally known to only code for PB1 protein. Following its discovery, the PB1-F2 protein rapidly received major attention from the influenza research community. Gibbs et al identified that the PB1-F2 protein localizes to mitochondria, causing cell death, and exemplifies the manner in which a virus can expand the coding capacity of its genome by using overlapping reading frames [11]. The PB1-F2 is predominantly localized in mitochondria, where it exerts proapoptotic and proinflammatory effects [12], disturbs the regulation of the innate immune response [13], and has IFN evasive and/or proinflammatory properties [14]. However, not all viruses encode PB1-F2. The Influenza Virus Resource (IVR) [15] of the National Center for Biotechnology Information (NCBI) estimated an average prevalence of approximately 90% for all published PB1 sequences, assuming that a functional PB1-F2 contains ≥79 amino acids (aa). This proportion varies among hosts (humans 90%, swine 76%, other mammals 100% and birds 95%) [16], [17]. Trifonov et al described that the H1N1pdm lacks a functional PB1-F2 because of a premature stop codon [18]. The PB1-F2 of this virus is thus only 11 aa in length.

ORF candidates of the influenza A virus identified after PB1-F2 include the PB1-N40 [19], PA-N155, PA-N182 [20], PA-X [21], NS3 [22] and M42 [23]. Studies have also described a hypothetical protein, NS1-NEG8 [24]. The PB1-N40 is a 718-aa truncated protein coded by leaky ribosome scanning by discarding the first 39 codons from the regular 757-aa PB1 [19]. This protein provides an example of a novel ORF translated in the same reading frame (in-frame) that begins at an alternative downstream AUG. The novel PA-related proteins PA-N155 and PA-N182 are also in-frame, which are translated from the 11^th^ and 13^th^ AUG codons in the PA transcript, discarding the first 154 and 181 codons from the regular 716-aa PA gene [20]. Jagger et al showed that translating PA mRNA can generate the novel protein PA-X through a frame-shifting event [21]. This ORF fuses a 191-aa translated product from position 1 to 573 of the PA transcript, and a 61-aa translated product from position 575 to 760, by shifting to the +1 reading frame (frame-2) on an UCC_UUU_CGU motif from position 568 to 576. Wise et al (2012) identified an M2-like product, M42, from a spliced variant of the MP gene [22]. This novel 99-aa protein, which has an alternative ectodomain, can functionally replace M2. Selman et al (2012) identified a novel donor splice site with a nucleotide (nt) substitution A374G in the NS gene, which leads to a novel 194-aa NS3 protein [23]. A putative NS1-NEG8 ORF is a 216-aa product translated from position 793 to 144 of the mRNA of the NS1 transcript in a negative direction [24]. However, the molecular mechanisms involved in its activity remain unclear.

Xu et al described a number of factors essential for identifying potential coding transcripts: the start codon position and the ORF length, as well as the Kozak consensus sequences [25]. They focused only on dual-coding proteins involving two overlapping ORFs. One was default to the original coding sequence that begins with an AUG, and the other came either downstream of the first AUG or extended upstream into the 5′ untranslated region (5′UTR). They additionally required both ORFs to be at least 500-nt long. While it is generally known that a strong Kozak sequence would increase the efficiency of protein translation [26]–[28], Xu et al investigated the Kozak strength combinations of the two ORFs and suggested that certain combinations would render both ORFs to exist. These rules, however, are not applicable for influenza viruses for the following reasons. First, it is already known that more than two ORFs can exist in an influenza genomic segment. For example, PB1, PB1-F2 and PB1-N40 all came from the same PB1 transcript. Second, the length of PB1-F2 is 90 aa (or 270 nt), which is considerably less than the 500-nt cutoff value. Third, most influenza virus sequences available from the database do not include their 5′UTRs. In other words, we cannot evaluate the Kozak sequence strength for the first AUG and any ones that may exist in the 5′UTR.

In this study, based on the ribosome scanning mechanism, we performed a computational investigation of all full-length coding sequences to list all possible ORFs in 3 forward-reading frames (frame-1, -2 and -3, also known as the in-frame frame-1, and two alternative-frame frame-2 and frame-3). The coding sequences researched included the traditional PB2, PB1, PA, HA, NP, NA, M1, M2, NS1 and NS2 transcripts. We did not investigate ORFs generated by a frame-shifting mechanism, such as in PA-X. Neither did we search for novel spliced mRNAs that lead to novel proteins such as NS3 and M42. However, we discussed any alternative initiation sites that might lead to novel ORFs from the 2 transcripts. We identified putative ORFs with ≥5% of the total sequence count of the translated products to eliminate the sporadic ORFs, and summarized them in maps, which schematically represent the ORF locations, lengths and Kozak sequences. Following the large count of novel ORFs meeting the 5% prevalence threshold, we further prepared an abridged list of putative ORFs for having an upstream start codon (within the upstream third of the transcript), a strong Kozak consensus sequence and a high prevalence (≥95% and ≥50% prevalence for in-frame and alternative-frame ORFs, respectively).

## Materials and Methods

### Coding sequences of the influenza A virus

All genome sequences of the influenza A virus were retrieved from the IVR [15] in October 2013. Full-length coding sequences that translate into their known protein products, including PB2, PB1, PA, NP, M1, M2, NS1 and NS2, were assessed. The HA and NA of each subtype were investigated separately because of massive sequence diversity between subtypes. H1–H18 and N1–N11 subtypes were evaluated. All sequences containing “N” were eliminated to avoid erroneous protein translation. Nucleotide coordinates used throughout this manuscript start from 1 for each investigated transcript.

### Preparation of NS3 and M42 transcripts

Wise et al identified a novel 300-nt M42 gene (including a stop codon, or 99-aa translated product), which fuses a 32-nt segment from position 89 to 120 of the M1 transcript and a 268-nt segment from position 715 to 982 [22]. Selman et al reported a novel donor splice site in the NS gene, which produces a novel 585-nt protein NS3 (including a stop codon, or 194-aa translated product) by fusing a 373-nt segment from position 1 to 373 and a 212-nt segment from position 503 to 714 [23]. These 2 rules were applied in this study to produce M42 and NS3 transcripts. The putative ORFs deriving from the 2 alternatively spliced transcripts were identified.

### Detection of open reading frames

For each of the collected influenza A transcripts, ORFs that begin with a start codon AUG and end with stop codons UAA, UAG or UGA were detected. These ORFs were sorted according to their start positions to identify the unique ORFs, which were further sorted into frame-1, -2 and -3 ORFs. Frame-1 ORFs are aligned (in-frame) with the original coding sequence, with downstream AUGs encoding identical, but shorter, protein sequences than those typically reported. Frame-2 and -3 (alternative-frame) ORFs encode different protein sequences to frame-1 ORFs (for example, PB1-F2 compared with PB1 protein). The ORFs were of various lengths, depending on where a stop codon was detected. A putative ORF was defined as having ≥5% prevalence in the sequence counts downloaded from the IVR. This specification avoided sporadic ORFs being detected because spontaneous mutations often occur in influenza A viruses.

### Kozak sequences in detected open reading frames

The rules of a Kozak sequence [19], [26] were as follows. In strong Kozak sequences, A/G (A or G) and G are the conserved nt at the third upstream (−3) and the fourth downstream (+4) positions from the start codon, respectively. For example, the consensus sequence “ACCAUGG” has “A” in position −3 and “G” in position +4. In medium Kozak sequences, A/G or G are at positions −3 and +4, respectively. All remaining nt combinations for positions −3 and +4 are classified as weak Kozak sequences. Since only coding sequences were included, no evaluation of the first AUG would be possible in this study.

### Protein database search for sequence and structural homologues

To test if any of the identified ORFs have a known protein homologue, BLASTP (version 2.2.29) [29] searches against a BLASTP database of non-redundant (NR) protein sequences downloaded from NCBI FTP site (ftp://ftp.ncbi.nih.gov/blast/db/) in June 2014 were performed on their translated aa products. The E-value and word size of the search were set at <10^−5^ and ≥3-aa length, respectively. In addition, we utilized an HHblits tool (version 2.0.15) [30] based on HMM-to-HMM comparisons against an HMM database of Protein Data Bank (PDB) [31] to search for structural homologues with default settings, including an E-value threshold of 0.001 and no less than 95% probability of being true positive. HHblits tool and database were implemented in the HH-suite which is an open-source software package (ftp://toolkit.genzentrum.lmu.de/pub/HH-suite/). PB1-F2 and putative ORFs containing partial PB1-F2 sequences were excluded for BLASTP and HHblits searches, due to that they will apparently hit to the sequences and structure (PDB ID 2HN8) of PB1-F2.

## Results

### Forward-reading open reading frames of viral transcripts

Table 1 lists data on all of the influenza A viral transcripts on which we performed ribosomal scanning for putative ORFs. We scanned approximately 20,000 sequences for the traditional viral coding segments, including PB2, PB1, PA, NP, M1, M2, NS1 and NS2. We manually assembled the transcripts for M42 and NS3 from the downloaded MP and NS primary transcripts, respectively. Although the sequence counts for M42 were comparable with those of other transcripts, only 512 NS3 transcripts were available for subsequent analysis. HA and NA transcripts were separately collected per subtype. The current influenza A sequence collection is considered to be highly biased in subtypes [32], as shown by the counts for the various HA and NA subtypes. For example, the HA subtypes were overwhelmingly dominated by H1, followed by H3, with counts as low as 3 and 1 for the newly identified H17 and H18, respectively. The NA subtypes were dominated by N1 and N2, whereas N10 and N11 had only 3 and 1 sequences, respectively.

**Table 1:** ORF data for influenza A viruses.

Gene	Length of transcripts(aa)	Sequence countsanalyzed	Total ORF counts(F1, F2, F3)	Initiation sites peramino acid (×10^−2^)
PB2	759	19,320	70 (37, 26, 7)	9.2
PB1	757	19,378	81 (38, 33, 10)	10.7
PA	716	20,404	77 (26, 35, 16)	10.8
NP	498	20,219	58 (26, 28, 4)	11.6
M1	252	28,216	24 (14, 8, 2)	9.5
M2	97	24,218	8 (2, 3, 3)	8.2
M42	99	18,349	8 (2, 3, 3)	8.1
NS1	230	23,125	28 (17, 7, 4)	12.2
NS2	121	21,536	15 (10, 2, 3)	12.4
NS3	194	512	16 (11, 4, 1)	8.2
H1	566	11,238	62 (9, 38, 15)	11.0
H2	562	468	76 (17, 44, 15)	13.5
H3	566	8025	69 (11, 46, 12)	12.2
H4	564	909	50 (6, 32, 12)	8.9
H5	568	1501	55 (17, 30, 8)	9.7
H6	566	1085	71 (16, 42, 13)	12.5
H7	560	750	67 (19, 36, 12)	12.0
H8	566	92	60 (14, 36, 10)	10.6
H9	560	1180	66 (16, 37, 13)	11.8
H10	561	509	57 (17, 27, 13)	10.2
H11	565	382	59 (8, 36, 15)	10.4
H12	564	124	60 (12, 34, 14)	10.6
H13	566	38	68 (12, 44, 12)	12.0
H14	568	11	42 (7, 29, 6)	7.4
H15	570	10	48 (14, 24, 10)	8.4
H16	565	31	65 (12, 38, 15)	11.5
H17	564	3	40 (11, 24, 5)	7.1
H18	561	1	39 (8, 23, 8)	7.0
N1	469	9918	55 (13, 25, 17)	11.7
N2	469	9651	52 (10, 27, 15)	11.1
N3	469	699	55 (9, 33, 13)	11.7
N4	470	188	60 (11, 30, 19)	12.8
N5	473	167	48 (10, 24, 14)	10.1
N6	470	1182	57 (15, 23, 19)	12.1
N7	470	471	44 (9, 22, 13)	9.4
N8	470	1362	61 (10, 31, 20)	13.0
N9	470	421	53 (7, 25, 21)	11.3
N10	442	3	28 (9, 12, 7)	6.3
N11	447	1	28 (6, 16, 6)	6.3

An ORF is defined as containing a start codon AUG at a given genomic position, with ≥5% prevalence from all analyzed sequences from the NCBI.

We performed ORF scanning on all viral transcripts shown in Table 1, and used the PB1 gene to standardize our scanning process. We retrieved 19,378 full-length sequences, all of which contained the complete 2274-nt PB1 coding segment (including the stop codon triplet). Forward scanning these sequences provided 284 ORFs, including the 757-aa PB1 protein at position 1. Each ORF began with AUG and ended with a stop codon, and could be uniquely identified by its genomic position based on the complete PB1 coding sequence. Eighty-one of the 284 ORFs had ≥5% (969) of the total sequence count (19,378 sequences), among which 78 ORFs had >12.99% prevalence (2519 sequences) (Table 1). By contrast, the sequence counts of the remaining 203 putative PB1 ORFs were all <2.99% (581 sequences), suggesting that a cutoff value of 5% frequency is effective for filtering sporadic ORFs, including those identified in other segments (data not shown).

Among the 81 putative PB1 ORFs that fulfilled the 5% prevalence threshold, we identified 38, 33 and 10 (shown in parentheses in Table 1) in reading frame-1, -2 and -3, respectively. The second-longest of the 38 in-frame (or frame-1) ORFs was PB1-N40. The in-frame ORFs are typically highly prevalent. For example, 35 have >95% prevalence, and the remaining 3 have 92%, 57% and 26%, respectively. We knew that all in-frame ORFs had not undergone early termination because early-terminated coding sequences would not have been deposited to the database.

The lengths of the frame-2 and frame-3 (or alternative-frame) ORFs varied. Therefore, we only reported the dominant lengths of such ORFs. Of the 33 frame-2 ORFs identified by analyzing all PB1 sequences, 25 had a prevalence ≥50%, including the well-known PB1-F2 and 2 short ORFs (8-aa sORF1 and 2-aa sORF2), which are reportedly associated with PB1-F2 and PB1-N40 synthesis [33]. Of the 10 frame-3 ORFs achieving the 5% prevalence threshold, only 2 had a prevalence ≥50%. Detailed prevalence, length and Kozak sequence statistics of PB1 ORFs can be found in S1 Table of S1 File.

**Figure 1:**
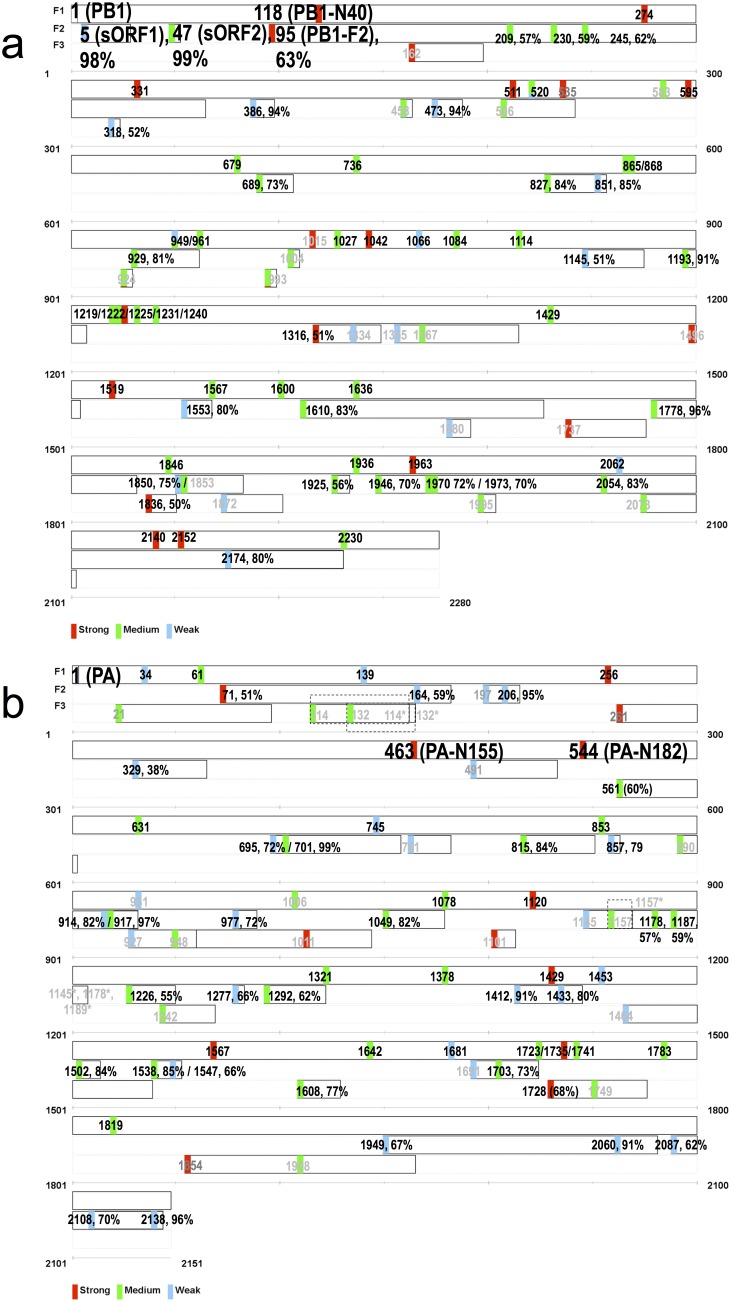
ORF maps of PB1 and PA genes. a) Eighty-one putative ORFs of PB1 gene are graphed in 3 forward-reading frames: F1, F2 and F3. Each colored box represents the start codon of an ORF, with red, green and blue indicating strong, medium and weak Kozak sequences, respectively. Numbers from 1 are used by each start codon to indicate positions. For ORFs with mixed populations of different Kozak strengths, multiple colors are used to represent the population proportions. For example, the ORF at position 520 is 74% green and 26% blue. F2 and F3 ORFs of major prevalence (>50%) are additionally labeled by their frequencies. ORFs of lower prevalence (<95% for F1, <50% for F2/F3) are grey-labelled. Each ORF ends with a solid line. For F2 or F3 ORFs of various ORF sizes, the corresponding dominant length is used to close the ORF. Four previously reported PB1 ORFs, PB1-F2, PB1-N40, sORF1 and sORF2, are bold-highlighted. b) Seventy-seven putative ORFs of the PA gene. The previously reported PA-N155 and PA-N182 are bold-highlighted at positions 463 and 544, respectively. Note that some ORFs overlap, such as those at positions 114 and 132. In such cases, 114* and 132*, in grey, are used to mark the end of the individual ORF, starting from position 114 and 132, respectively.

To visually summarize these results, we graphed the 81 PB1 putative ORFs in Fig. 1a. We bold-labelled the ORFs reported in previous studies, including PB1 and PB1-N40, and 3 frame-2 ORFs (PB1-F2, sORF1 and sORF2), which represent the first 5 AUGs in the entire PB1 gene. We graphed other newly identified ORFs according to their start positions, indicating Kozak strength (red: strong; green: medium; blue: weak). We labelled more abundant frame-2 and -3 ORFs with prevalence ≥50% according to their frequencies. For example, the seventh (nt position 209), eighth (230) and ninth (245) AUGs are ORFs with frame-2 translations. They are in-frame with PB1-F2 and shorter because of their downstream reinitiations. Their prevalence are comparable (57–62%) with that of PB1-F2 (63%). Note that PB1-F2 is only considered intact when it is >57 aa in length. When we calculated the percentage 63% we only included the dominant 90-aa PB1-F2 proteins from 19,378 PB1 sequences. We grey-labelled the ORFs with low prevalence (<95% in-frame and <50% alternative-frame) in Fig. 1a to highlight the abundant ORFs. For example, we grey-labelled 3 low prevalence frame-1 ORFs, according to their start codon locations, as 583 (92%), 535 (57%) and 1015 (26%), respectively. The 8 low prevalence frame-2 ORFs were at positions 458 (44%), 506 (2%), 1004 (41%), 1314 (40%), 1355 (47%), 1367 (48%), 1496 (42%) and 1853 (23%). The only 2 frame-3 ORFs with >50% prevalence were 318 (52%) and 1836 (50%).

Fig. 1b shows the putative ORF map for PA, which we obtained by analyzing 20,404 complete 2151-nt PA coding segments (including the stop codon triplet). The complete PA coding segment encodes a 716-aa PA protein and a 252-aa PA-X protein. Seventy-seven of 318 identified ORFs had ≥5% prevalence (1020 sequences), among which 26, 35 and 16 were reading frame-1, -2 and -3, respectively. The 2 in-frame ORFs (PA-N155 and PA-N182) and the primary translated product PA are bold-highlighted in Fig. 1b. Other conventions used in Fig. 1b are the same as those applied in Fig. 1a. Note the 2 frame-3 ORFs beginning at positions 114 and 132 (labeled in grey). Recall that we graphed ORF length according to the dominant length only. Although we identified that 20,347 of all 20,404 analyzed PA transcripts have a start codon at position 114, this ORF has variable lengths, including 8086, 6186 and 5116 with 16-aa, 17-aa and 20-aa lengths, respectively, in which the dominant length 16-aa was used to graph this ORF in Fig. 1b. Similarly, we observed 4635 PA transcripts encoding an ORF beginning at position 132, including 3420, 951 and 197 with 11-aa, 10-aa and 22-aa lengths, respectively. As a result, this ORF was graphed by its dominant length of 11-aa. Consequently, the 2 ORFs at position 114 (of 16-aa long) and 132 (of 11-aa long) overlapped because their start codons were only 6 aa apart. Graphically we used “114*” and “132*” to signal the end of these two overlapping ORFs. This also explained the using of grey labels for these two ORFs, because each was computed for a prevalence of less than 50% by using only their ORFs of dominant length (8086/20,404 = 39.6% for ORF at position 114, and 3420/20,404 = 16.8% for ORF at position 132). S2 Table in S1 File provides details on the 77 PA ORFs.

Table 1 lists the putative ORF counts for all 39 influenza A transcripts investigated in this study. In general these counts ranged from approximately 6.3–16.2×10^−2^ per amino acid (or 6.3–16.2 per 100 aa). Therefore, we considered that an influenza A transcript would have the potential to encode an ORF every 6–16 codons on average. Following the criteria and conventions used in Fig. 1, we summarized the putative ORFs for the remaining influenza A transcripts in our Supporting Materials. S1–S8 Figs. of S2 File show ORF maps for the PB2, NP, M1, M2, M42, NS1, NS2 and NS3 transcripts. S9–S26 and S27–S7 Figs. in S2 File show the putative ORF maps for the 18 HA and 11 NA subtypes, respectively. S3–S39 Tables in S1 File provide supplementary information on the putative ORFs in S1–S37 Figs. in S2 File.

### Abridged open reading frame candidates for the ribosomal scanning mechanism

Table 1, Fig. 1, S1–S37 Figs. in S2 File and S1–S39 Tables in S1 File show all 39 ORF maps and summarize the putative influenza A virus ORFs. A total of 1982 influenza A virus ORFs were thus documented. Previous studies have suggested that a strong Kozak sequence, suitable start codon location and high prevalence are equally essential in a putative ORF. Consequently, we screened the 1982 ORFs with a strong Kozak consensus sequence, an upstream AUG location (within the upstream third of the transcript), and high prevalence (≥95% and ≥50% prevalence for in-frame and alternative-frame ORFs, respectively). We named the resulting ORFs according to known influenza ORF nomenclature conventions. For example, investigators named PB1-N40 because it starts at the 40^th^ codon of a PB1 transcript [19]. We named a 666-aa frame-1 PB1 ORF as PB1-N92 because it is 91 aa shorter than the regular 757-aa PB1 protein. We named other frame-2 and frame-3 ORFs in the same manner as PB1-F2. Excluding the 4 known ORFs (PB1-F2, PB1-N40, PA-N155 and PA-N182), Table 2 shows data on 16 ORFs, except for HA and NA, for which we identified 43 HA and 11 NA ORF candidates for 18 HA and 11 NA subtypes and listed them in S40 Table of S1 File. These are considered the most probable novel ORFs to emerge in influenza A viruses from those 1982 putative ORFs listed in Table 1.

**Table 2:** An abridged ORF list with additional constraints.

ORF	Position (Frame)	Dominant length (aa)	Prevalence (%)
PB2-N51	151 (1)	709	99
PB2-N90	268 (1)	670	98
PB2-F3	291 (3)	3	72
PB2-F2	305 (2)	23	75
PB2-N164	490 (1)	596	99
PB1-N92	274 (1)	666	99
PB1-N111	331 (1)	647	97
PB1-N171	511 (1)	587	96
PB1-N199	595 (1)	559	99
PA-F2	71 (2)	37	51
PA-N86	256 (1)	631	97
NP-N66	196 (1)	433	99
NP-F2	302 (2)	4	52
NP-F3	309 (3)	8	53
NP-N159	475 (1)	340	99
M1-F3	132 (3)	12	81

The ORFs in Table 1 were screened with additional filters, strong Kozak consensus sequence, upstream AUG location (within the first third of the transcript), and high prevalence (≥95% and ≥50% for in-frame and alternative-frame ORFs, respectively), to obtain 16 novel ORFs. These ORFs were named, according to conventions, as PB1-N40, PA-N155, PA-N182 (in-frame) and PB1-F2 (alternative-frame). The 4 known ORFs are excluded from this table.

All of the ORFs in Table 2 derive from PB2, PB1, PA and NP genes, except for one that derives from M1. All PB1 ORFs are in-frame as in PB1-N40; therefore, PB1-F2 was the only alternative-frame ORF to fulfill the filtering criteria applied in Table 2. All in-frame ORFs were highly prevalent (96%–99%). We identified one frame-2 (PB2-F2) and one frame-3 (PB2-F3) ORF in the PB2 gene, in which PB2-F3 was at a slightly upstream position of 291 than PB2-F2’s 305. According to Table 2, the 3-aa PB2-F3 was found in 72% of all PB2 sequences analyzed. However, other PB2-F3 sizes included 22 aa (in 1276 sequences, all with strong Kozak sequences), 18 aa (in 219 sequences, of which 118 had strong and 101 had medium Kozak sequences), and other sporadic PB2 sequences of various lengths or Kozak strengths (S3 Table). In other words, some ‘extended’ PB2-F3 would contain common RNA segment with PB2-F2 located only 14 nt downstream. Furthermore, the dominated 3-aa PB2-F3 might simply be ‘truncated’ forms of those with 18-aa or longer translated products.

Turning to PB2-F2, in addition to the 75% PB2 sequences containing a 23-aa product, the ORF sizes also included 3 aa (3851 sequences, all strong Kozak), 20 aa (59 sequences, all strong Kozak), and other sporadic sequences (S3 Table). We described the 4 remaining alternative-frame ORFs according to their various lengths and Kozak strengths (details in S2, S4 and S5 Tables in S1 File) below. The dominant PA-F2 ORFs are 37 aa in length (in 51% of all PA sequences). Other PA-F2 ORF sizes are 4 aa (5099 sequences, all strong Kozak), 18 aa (3187 sequences, strong Kozak), 39 aa (747 sequences, strong Kozak), 30 aa (276 sequences, strong Kozak), 6 aa (100 sequences, strong Kozak) and others. The dominant NP-F2 size is 4 aa (in 52% of the NP sequences), followed by 4 aa (5254 sequences, medium Kozak), 7 aa (199 sequences, strong Kozak), and others. The dominant NP-F3 size is 8 aa (in 53% of the NP sequences), followed by 41 aa (959 sequences, strong Kozak), 8 aa (865 sequences, medium Kozak), 21 aa (193 sequences, strong Kozak), 20 aa (139 sequences, strong Kozak), and others. The dominant M1-F3 is 12 aa in length (in 81% of the M1 sequences), followed by 26 aa in length (314 sequences, strong Kozak), and others.

### Correlation between open reading frame length and viral phenotype

**Figure 2:**
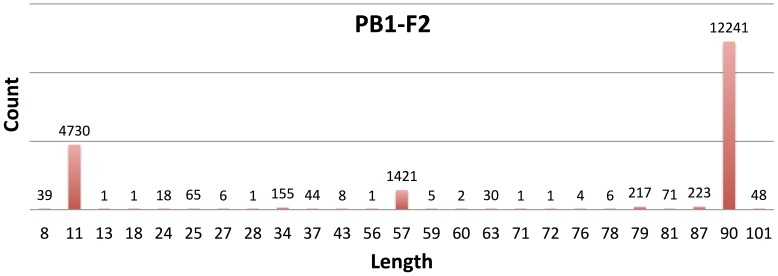
Length variability of PB1-F2. Of 19,378 PB1 sequences analyzed, 19,339 contain a start codon at position 95, signaling the beginning of the PB1-F2 ORF. The downstream stop codons vary, resulting in different ORF sizes. The 3 major PB1-F2 lengths are 90, 57 and 11 aa. It is generally assumed that an intact PB1-F2 is ≥79 aa in length.

The preceding two paragraphs illustrated the genetic characteristics of novel ORFs from frame-2 and frame-3. The inherited genetic diversity (through the accumulation of spontaneous mutations) of influenza A viruses have resulted in various appearing of stop codons in the reading frame, leading to different lengths for the same ORF. Not only such complexity presented difficulties in preparing a comprehensive ORF map/table in terms of length and prevalence, the variations in ORF length might also be associated with certain viral phenotypes, such as virus subtypes or hosts from which the viruses were isolated. Fig. 2 uses PB1-F2 as an example, and shows the distributions of lengths of all 19,339 PB1-F2 ORFs from 19,378 PB1 transcripts. Dominant lengths included 90 aa (12,241 sequences), 57 aa (1421 sequences) and 11 aa (4730 sequences). Minor lengths included 87 aa (223 sequences), 79 aa (217 sequences) and 34 aa (155 sequences). Of the 4730 11-aa PB1-F2 ORFs, 4090 ORFs were derived from human H1N1pdm sequences isolated in 2009 or later. Of 1421 57-aa ORFs, 1047 were derived from human H1N1 sequences isolated prior to 2009 (exclusive). These observations suggested that a truncated PB1-F2 ≤57 aa in length was predominantly derived from human H1N1 viruses. Therefore, non-H1N1 human and other nonhuman viruses contributed to the PB1-F2 of other lengths, predominantly those ≥79 aa. S1 Table in S1 File contains length statistics of PB1-F2 that led to Fig. 2. It, along with S2–S39 Tables in S1 File, provide data on the lengths of all other putative ORFs. Based on the multiple sequence alignment for a given transcript and associated data we included in these supporting tables, one would be able to investigate how a given ORF may correlate to certain phenotype, as we have demonstrated on PB1-F2 here.

### BLASTP searches for database homologues of putative open reading frames

We used the putative ORFs in Table 1 to query the NR database by using BLASTP, except for the frame-1 ORFs because they would match the longest proteins from their respective primary transcripts. We also excluded PB1-F2 and any of its in-frame downstream ORFs (at positions 209, 230 and 245, as shown in Fig. 1a) because they would hit to the PB1-F2 proteins that are already well-annotated in the database.

Table 3 shows the BLASTP hits to the NR database, including the query sequence (the ORF) and the subject (the highest scoring match returned from the database). We grouped the search results in Table 3 into 8 distinct categories for subsequent discussion. Case 1 has PB1 ORFs at position 47, which hit to a number of PB1-F2 proteins. According to Fig. 1a, this is a short ORF, 2 aa in length, and referred to as sORF2 [33]. However, among the 19,378 PB1 transcripts analyzed, we observed sporadic strains whose sORF2 were >2 aa in length. In fact, 26 such ORFs were 50 aa (one strain), 73 aa (7 strains), 95 aa (6 strains) or 106 aa (12 strains) in length. Because this ORF is upstream of PB1-F2 by only 16 aa, the 26 long sORF2 ORFs overlapped with PB1-F2 and thus locally aligned to PB1-F2 in the database. The 26 viruses included 15 swine influenza A viruses (11 H3N2, 2 H1N1, one H1N2 and one H5N1), 6 avian viruses (3 H5N1, one H2N2, one H5N2 and one H9N2), 4 human viruses (3 H3N2 and one H7N9) and one environment sample (H5N1).

**Table 3:** BLASTP hits against the NR database for all putative ORFs.

Case	Gene/Position (frame)	Accession numbers of database hits	Database hit info
1	PB1/47 (2)	Q288Y7, P0C5U7, AGR49480, AGO51397, AFV71377, AFR42732, AFJ12638, AEZ01190, AEX35415, AEO89179, ACL12403, ACJ26075, ACH58918, AGQ83473, AFO83333, ACO36494, AHB51242, AHB22709, ABI85213	PB1-F2
2	PB1/1925 (2), PB1/1946 (2), PB1/1970 (2), PB1/1973 (2), PB1/2054 (2)	AHB23179, AHB23700, AHB23730, AHB23769, AHB23797, AHB23845, AHB24489, AHB24622, AHB51934, AHB51987, AHB52010, AHB52086, AHB52204, AHB52240, AHB52291	PB1-F2
3	PA/1949 (2)	BAA01430	DI-2 protein, A/WSN/1933(H1N1)
4	H3/1596 (3)	AAA72667, AAA72249	Fusion protein, synthetic construct
5	PB1/1680 (3), PB1/1737 (3)	AEI29961	A/environment/Korea/CSM3/2002(H3N6)
6	NP/605 (2), NP/632 (2), NP/656 (2)	AAV68025	A/swine/Korea/S452/2004(H9N2)
7	H4/1277 (2)	AAA43224	A/seal/Massachussetts/133/1982(H4N5)
8	N2/728 (3)	ACA04672	A/duck/Eastern China/48/2002(H11N2)

All hits for the putative ORFs listed in Table 1 are grouped into 8 cases according to the queried ORFs and hits returned from the database.

Case 2 had 5 PB1 ORFs at downstream positions 1925, 1946, 1970, 1973 and 2054, which hit to PB1-F2 sequences in the database. These ORFs are all frame-2 ORFs with dominant lengths of 3, 95, 87, 86 and 59 aa (S1 Table in S1 File), and seemingly high 56%, 70%, 72%, 70% and 83% prevalence, respectively. Similar to the sORF2 in Case 1, a small proportion of 102 PB1 strains yielded a long 50-aa ORF starting at position 1925 instead of the commonly seen 3-aa ORFs. These extended ORFs at position 1925, along with the other 4 long and overlapping ORFs (59 to 95-aa) at positions 1946 to 2054, shared a common aa segment that was found to align to the reported database sequences (Table 3). The manner in which these ORFs matched to PB1-F2, which is located far upstream of the PB1 coding sequence, is unclear. When we inspected the 15 PB1-F2 sequences returned from the database, we observed that they were all translated from partial PB1 sequences and that the corresponding nt sequences were all aligned to a downstream full-length PB1 sequence (based on PB1 of A/Puerto Rico/8/34) rather than the location at which PB1-F2 would be expected to appear. Upon further cross-checking, we observed that these 15 PB1-F2 sequences showed no similarity to the other PB1-F2 sequences (data not shown). We consider these 15 PB1-F2 sequences to have been erroneously annotated in the database. These downstream PB1 ORFs are thus deemed dissimilar to the commonly known PB1-F2.

Case 3 reports the BLASTP results for a frame-2 PA ORF at position 1949. This ORF is located near the C-terminus with a dominant length of 44 aa, and occurs in 67% of the total PA sequences analyzed. More than 99% of these 44-aa ORFs hit to a 40-aa C-terminal segment from a 93-aa DI-2 protein (BAA01430), which was translated from a 432-nt record D10573 (CDS 25..306). D10573 is derived from a laboratory strain (A/WSN/1933(H1N1)) which joined 2 PA segments: one from the 5′ end and one from the 3′ end. Therefore, the C-terminal part of this translated 93-aa DI-2 protein shares high similarity with our frame-2 ORF at position 1949. Because this DI-2 protein is a laboratory-derived product and the only one in the database that matches our ORFs, we do not consider this particular PA ORF to have any homologues in the database. Similarly, Case 4 reports a frame-3 H3 ORF at position 1596 hit to 2 synthetic constructs: AAA72249 (an HA mutant based on A/Udorn/72(H3N2) with deletions at the 3′ end), which is 31 aa in length, and AAA72667 (a human transferrin receptor/influenza hemagglutinin fusion protein based on A/Victoria/3/75(H3N2)), which is 38 aa in length. We thus concluded that no homologues of this H3 ORF exist in wild-type influenza A viruses or other organisms.

Cases 5–8 report different frame-2 or frame-3 ORFs, each matched to a number of influenza A sequences in the database. The numbers of ORFs responsible for the database hits vary. For example, we observed that 1510 PB1 ORFs at position 1680 (Case 5) hit to the PB1 protein of A/environment/Korea/CSM3/2002(H3N6), and that only 2 H4 ORFs at position 728 (Case 7) hit to the HA protein of A/seal/Massachusetts/133/1982(H4N5). To determine the manner in which a frame-2 or frame-3 ORF hits to a frame-1 database sequence deriving from a wild-type virus, we compared the 4 database sequences with other wild-type viruses and observed that they all contain single-nt insertions or deletions in the gene segment of interest (S38 Fig. in S2 File). In other words, a frame-shifting occurs in the event of an insertion or deletion in these reported wild-type strains. Consequently, a very small portion of these database sequences got translated into frame-2 or frame-3 products, which resembled our frame-2 and frame-3 ORFs. Since such resemblance was really rare in wild-type viruses, we do not consider them to be new homologues matched to our putative ORFs.

### HHblits searches for structural homologues of putative open reading frames

HHblits was used to search PDB database for structural homologues with default settings. Fifteen structural hits were found for 20 putative ORFs complying an E-value threshold of 0.001 and no less than 95% true positive probability. A number of these structural hits from some abundant ORFs were demonstrated in Table 4, while others were included in S41 Table of S1 File. For example, a dominated case in Table 4 is three frame-2 PB1 ORFs (PB1-1946, PB1-1973 and PB1-2054) as well as two frame-2 NP ORFs (NP-1103 and NP-1109) hitting to 1WRG (Light-Harvesting Complex 1 Beta Subunit from Wild-Type Rhodospirillum rubrum). The counts of these PB1 and NP ORFs hitting to this structure are 1222 and 42, respectively. It is mentioned that frame-2 and frame-3 translations can turn synonymous mutations originally seen in frame-1 into non-synonymous ones. As a result, certain degree of sequence diversity exists in the same ORF from different strains, leaving a slightly different E-value, sequence identity and positive probability being observed for these ORFs. Accordingly, E-value, sequence identity and probability were provided in Table 4 by averaging ones from these many PB1 or NP ORFs hitting to 1WRG. To display how the query and PDB sequence were matched, a representative alignment with the most significant E-value was also presented. Other found structural homologues shown in Table 4 included 32 H1-1316 ORFs hitting to zinc-binding domain of E. Coli and 40 N7-282 ORFs hitting to eIF2alpha protein kinase of yeast.

**Table 4:** HHblits hits against PDB database for alternative-frame ORFs.

Query (Count)/Frame No.	Avg. E-value	Avg. identity (%)	Avg. prob. (%)
PDB ID: Definition/Classification/Organism/Molecule Name			
One representative alignment			
PB1-1946 (22), PB1-1973 (6), PB1-2054 (1194)/Frame-2	6.74E-05	68.7	95.7
a1WRG: Light-Harvesting Complex 1 Beta Subunit/Membrane Protein/Rhodospirillum rubrum/Light-harvesting protein B880, beta chain			
PB1-2054 8 AAIYLKSFSPVVHIGGQLEFLAW-WRPWCPGPELM 41(59)			
Consensus 8 ∼∼iF∼∼sf∼aFvaIAvvAHvLaW-WRPWlPG∼eG∼ 41(59)			
−++|++||++|++||++||+|+| ||||+|||||−			
Consensus 20 H∼∼f∼∼∼∼∼∼f∼∼iA∼vAH∼L∼w∼wrPWlpg∼∼g∼ 54(55)			
1WRG 20 HKIFTSSILVFFGVAAFAHLLVWIWRPWVPGPNGY 54(55)			
NP-1103 (5), NP-1109 (37)/Frame-2	3.11E-06	53.0	96.7
a1WRG: (Same as above)			
NP-1109 1 MWKPWIPTP 9 (9)			
Consensus 1 ∼wRPWlpg∼ 9 (9)			
+||||+||+			
Consensus 43 ∼wrPWlpg∼ 51 (55)			
1WRG 43 IWRPWVPGP 51 (55)			
H1-1316 (32)/Frame-2	1.82E-07	50.5	97.3
2DS5: Structure of the ZBD in the orthorhomibic crystal form/Metal Binding Protein, Protein Binding/Escherichia coli/ATP-dependent Clp protease ATP-binding subunit clpX			
H1-1316 2 LNCSFCSKTK 11 (13)			
Consensus 2 ∼∼CSFCGk∼∼ 11 (13)			
.+||||||++			
Consensus 12 ∼∼CSFCGk∼∼ 21 (51)			
2DS5 12 LYCSFCGKSQ 21 (51)			
N7-282 (40)/Frame-3	5.00E-06	42.0	96.6
1ZY4: Crystal Structure of eIF2alpha Protein Kinase GCN2/Transferase/Saccharomyces cerevisiae/Serine/threonine-protein kinase GCN2			
N7-282 1 MGSGGQGQCYKI 12 (13)			
Consensus 1 i∼∼g∼∼∼∼v∼∼∼ 12 (13)			
||+|+||+||+|			
Consensus 14 lG∼G∼fg∼V∼∼∼ 25 (303)			
1ZY4 14 LGQGAFGQVVKA 25 (303)			

HHblits hits were grouped by PDB ID or putative ORF. E-value, sequence identity and probability were provided by averaging ones from HHblits hits. The alignments between putative ORFs and structures were generated by HHblits tool. The number in parenthesis following a sequence alignment is the length of either query or subject. Consensus sequences are shown in HMM format for both the query and the subject, in which capital and lowercase letters are used to represent conserved columns with ≥60 and ≥40 probabilities, respectively, and a tilde “∼” is used to represent non-conserved column. Based on default settings with two iterations, HHblits tool adds significant hit(s) from the previous search/iteration to the HMM query for the next search/iteration. It results to that our query may not be single sequence. Five symbols are used to show the alignment quality, in which “|”, “+”, “.”, “−” and “ = ” each represents a quality from the perfect to the worst.

a Two groups of putative ORFs from PB1 and NP genes hit to 1WRG structure.

## Discussion

We based our hypothesis for identifying novel ORFs on the leaky ribosomal scanning mechanism, which uses a downstream AUG to initiate the translation rather than the first AUG encountered in the mRNA. The PB1-F2 provides an example of an ORF that uses the fourth AUG of PB1 to encode a frame-2 protein, and has been shown to be involved in several aspects of influenza virology. Studies on the PB1-N40, PA-N155 and PA-N182 and associated functions have suggested that other in-frame ORFs initiated at downstream AUGs might exist and play crucial roles. It appears that influenza A viruses are free to encode either in-frame (frame-1) or alternative-frame (frame-2 and -3) ORFs. Note that the identification of putative ORFs by only scanning AUGs downstream of the major product of each segment would omit a number of new proteins being identified. For example, we considered PA-X one such transcripts from PA, which was obtained from skipping the 574^th^ nucleotide of the regular PA transcript. We also considered a number of NS and MP spliced transcripts for searching their downstream AUGs. These alternative transcripts (via frame-shifting or splicing) must be known first before including them into our ribosomal scanning algorithm.

The influenza virus is an RNA virus that undergoes spontaneous mutations, resulting in sequence diversity that complicates finding ORF start codons. These mutations also affect corresponding stop codons for determining ORF sizes. Therefore, a single mutated nt can often hide an AUG, or truncate or elongate an ORF in some particular influenza A viruses without affecting the others. These events commonly occur in frame-2 and frame-3 ORFs. In short, some ORFs may exist only in a subpopulation rather than the entire set of influenza A viruses. The prevalence of a putative ORF provides an indicator of its coding potential. The more frequent an AUG is seen at a given genomic location, the more likely this particular ORF is crucial for influenza viruses. Note that the variable ORF sizes as well as how the sequences were sampled can perplex the presentation of a prevalence. For example, Zell et al identified 1930 PB1 sequences encoding PB1-F2 based on 2226 PB1 mRNAs collected in June 2006. The prevalence was 87%, based on requiring a PB1-F2 be at least 79-aa long [17]. Trifonov et al indicated that all 2009 H1N1pdm influenza A viruses encoded 11-aa PB2-F2. They required a PB1-F2 ≥57 aa in length, thus concluded the absence of PB1-F2 in these viruses [18]. In Fig. 2 we showed that 19,339 PB1-F2 ORFs of variable lengths were encoded from 19,378 PB1 sequences. They were mostly of 90-aa long, followed by a few noticeable ones of 87-aa, 79-aa, 57-aa and 11-aa long. We mentioned that an ORF length was presented based on the most dominant length in this study. As a result PB1-F2 was labeled 90-aa long, and a prevalence computed as 63% (from 12,241 90-aa ORFs out of 19,378 sequences) in Fig. 1a. The prevalence would increase to 73% if we included all ORFs ≥57 aa in length, and nearly 100% if we counted the PB1-F2 of all sizes. The reasons that previous reports only took 57-aa or longer PB1-F2 into consideration is that these truncated forms from their longer (mostly 90-aa) versions maintained key PB1-F2 function domains (such as the mitochondrial targeting sequence (MTS), etc.). For other novel ORFs we identified in this study, however, no functional studies are available to support how long an ORF can be considered intact. Wise et al demonstrated the two short PB1 ORFs (8 aa and 2 aa, referred as sORF1 and sORF2, respectively) helped in regulating PB1-F2 and PB1-N40 synthesis [33]. For these reasons, we did not require a putative ORF to meet a minimum length threshold.

A strong Kozak sequence is known to promote the initiation of translation. The PB1-F2, PB1-N40, PA-N155 and PA-N182 all contain strong Kozak sequences. Keep in mind, however, that a strong Kozak sequence is not required for some influenza ORFs. For example, the two mentioned PB1 sORF1 and sORF2 each had weak and medium Kozak consensus, respectively. Kozak et al [34] also summarized that leaky ribosomal scanning can occur far from the first AUG, such as in the peanut clump, southern bean mosaic and rice tungro bacilliform viruses. The second AUG of these viral mRNAs can be as far as 500 nt downstream from the first AUG [34]. Indeed that PA-N182 is the 13^th^ AUG starting at position 544 of a full-length 2151-nt PA transcript. No literatures seem to rule out the possibility that an ORF can occur at more downstream positions. For these reasons, we did not require an ORF to have strong Kozak sequence, nor did we restrict an ORF to appear only at some upper stream locations for the entire transcript. Our only specification was a 5% prevalence in all available database sequences analyzed for filtering out sporadic ORFs. Currently, >20,000 sequences are available for the majority of influenza A virus genes. According to our threshold, >1000 viruses would render one particular ORF. This leads to the ORFs we summarized in Table 1, Fig. 1 and accompanying S1–S37 Figs. in S2 File and S1–S39 Tables in S1 File. We consider these to contain the most comprehensive influenza ORF data that can be used as reference material by the research community. Nevertheless, we presented an abridged ORF list in Table 2 (as well as in S40 Table in S1 File) which fulfills 3 criteria: an upstream start position (within the upstream third of the transcript), strong Kozak sequence and high prevalence (≥95% and ≥50% prevalence for in-frame and alternative-frame ORFs, respectively). These would be in general the most probable influenza A ORFs by satisfying these stringent conditions.

While a BLASTP search for all frame-2 and -3 ORFs did not seem to find anything more than the known PF1-F2, HHblits helped revealing a number of structural homologues from PDB as shown in Tables 4 and S41 Table in S1 File. This tool utilizes hidden Markov model to group PDB structures into consensus profiles, enabling a hit to be found even the sequence identities are as low as 14% between a query and a database subject. Although the found matches apparently met the computational criteria incorporated in HHblits for statistical significance, the aligned regions were all local and fragmented to include only discrete secondary structures. Whether they may suggest known functions to those putative ORFs of influenza A viruses would certainly require further justification.

Finally we elaborate more about a number of short ORFs in Table 2. As shown in this table, PB2-F3 is 3 aa in length, with 72% prevalence. Although the high prevalence indicates it a common ORF in influenza A viruses, this ORF could be too small to contain any functional domains (although short ORFs can play some regulatory roles, such as the 2-aa sORF2 in synthesizing PB1-N40 and PB1-F2). We observed that 1276 (7%) PB2 sequences contain a 22-aa PB2-F3 (S3 Table in S1 File). If this is ORF is functional, the dominant 3-aa counterparts might be simply a truncated form of PB2-F3, similar to the 11-aa version of PB1-F2. Similarly, in NP-F3 and M1-F3, the second-dominant ORF sizes are 41 aa and 26 aa, respectively, suggesting that the dominant ORFs (8 aa and 12 aa) might be also truncated forms. These examples showed that some short ORFs might be listed simply because the short ones dominated the entire ORF population. Our Supporting Tables provide details to assist with interpreting the provided ORF maps and tables.

## Conclusion

In this study, we performed a computational search, based on ribosome scanning, of all full-length coding sequences to investigate all possible ORFs in 3 forward-reading frames. An ORF is defined by labelling a start codon AUG at a specific genomic location. The scanning procedure eliminated sporadic ORFs by requiring ≥5% prevalence of the total number of sequences analyzed for an individual transcript. We summarized all of the putative ORFs in maps, which schematically represent their locations, lengths and Kozak sequences. We further prepared an abridged list of putative ORFs by additionally requiring each of them to have an upstream start codon (within the upstream third of the transcript), strong Kozak consensus sequence and high prevalence (≥95% and ≥50% prevalence for in-frame and alternative-frame ORFs, respectively). Although we focused on the ribosomal scanning of known transcripts in this study, we cannot eliminate that other mechanisms, such as alternative splicing and frame-shifting, can identify additional ORFs.

The complexity and variability of the ORFs can be attributed to the continuous changing of genomic outfits by RNA viruses. Thus, our maps and tables should be routinely updated for novel ORFs. Our data can be used to specifically follow-up an ORF of interest. For example, over the years, investigators have monitored variations in length, amino acid substitutions and host or subtype specificities in PB1-F2. Additional analyses and laboratory studies are required to confirm that the ORFs proposed in this study are produced and to investigate their roles in influenza virology.

## Supporting Information

S1 File **S1–S39 Tables. Length and count data for influenza A putative ORFs accompanying** **Figs. 1a** **and** **1b** **, and S1–S37 Figures.** ORFs are listed according to their start codon positions in nt in their corresponding transcript. For an ORF of various lengths, multiple entries are displayed, ordered by the sequence counts per ORF length. For example, in S1 Table, ORFs starting at position 5 are grouped into 4 rows containing 19 148 sequences of 8 aa, 16 sequences of 16 aa, 13 sequences of 5 aa and 3 sequences of 3 aa in length. **S40** **Table. An abridged ORF list of influenza HA and NA genes.** The filtering criteria used in Table 2, strong Kozak consensus sequence, an upstream AUG location within the upstream third of the transcript and high prevalence (≥95% and ≥50% for in-frame and alternative-frame ORFs, respectively), were applied to obtain the most probable HA and NA ORF candidates from Figs. S9–S37 (S11–S39 Tables). Because some of the genes have multiple frame-2 or frame-3 ORFs, we did not name the ORFs according to Table 2. Instead, their starting positions are provided for identification purposes. **S41** **Table. HHblits hits against PDB database for alternative-frame ORFs.** This table supplements Table 4 for the hits of structural homologues to all frame-2 and -3 influenza A novel ORFs. E-value, sequence identity and probability were provided by averaging ones from HHblits hits. The alignments between putative ORFs and structures were generated by HHblits tool. The number in parenthesis following a sequence alignment is the length of either query or subject. Consensus sequences are shown in HMM format for both the query and the subject, in which capital and lowercase letters are used to represent conserved columns with ≥60 and ≥40 probabilities, respectively, and a tilde “∼” is used to represent non-conserved column. Based on default settings with two iterations, HHblits tool adds significant hit(s) from the previous search/iteration to the HMM query for the next search/iteration. It results to that our query may not be single sequence. Five symbols are used to show the alignment quality, in which “|”, “+”, “.”, “−” and “ = ” each represents a quality from the perfect to the worst.
(PDF)

S2 File **S1–S37 Figures. ORF maps of PB2, NP, M1, M2, M42, NS1, NS2, NS3, H1–H18 and N1–N11 transcripts.** Symbols and legends used are as defined in Figure 1. Briefly, the putative ORFs are graphed in 3 forward-reading frames: F1, F2 and F3. Each colored box represents the start codon of an ORF, with red, green and blue indicating strong, medium and weak Kozak sequences, respectively. Numbers from 1 indicate the positions of start codons. For ORFs with mixed populations of different Kozak strengths, multiple colors indicate the population proportions. F2 and F3 ORFs of major prevalence (>50%) are additionally labelled by their frequencies. ORFs of lower prevalence (<95% for F1, <50% for F2/F3) are grey-labelled. Each ORF ends with a solid line. For F2/F3 ORFs of various sizes, the corresponding dominant length is used to close the ORF. For overlapping ORFs, an asterisk (*) accompanying the start position is additionally labelled in grey to mark the end of the ORF. **S38** **Figure. Nucleotide alignments for the BLASTP reports.** Four nt sequence alignments are used to illustrate the single nt insertions or deletions (red) that disturbed the reading frames, producing database hits to the ORFs (Table 3). (a) Corresponds to Case 5 in Table 3. Query = JN087010, which translates into AEI29961 in Table 3. Sbjct = HM145493, the second-closest hit in the NT database, excluding the query sequence itself. b) Corresponds to Case 6 in Table 3. Query = AY790308, which translates into AAV68025 in Table 3. Sbjct = AY862651, the second-closest hit in the NT database, excluding the query sequence itself. c) Corresponds to Case 7 in Table 3. Query = M25291, which translates into AAA43224 in Table 3. Sbjct = CY005956, the second-closest hit in the NT database, excluding the query sequence itself. d) Corresponds to Case 8 in Table 3. Query = EU429730, which translates into ACA04672 in Table 3. Sbjct = EU429760, the second-closest hit in the NT database, excluding the query sequence itself.
(PDF)
