# Supplementary material for: Computational Analysis and Mapping of Novel Open Reading Frames in Influenza A Viruses
Source: PLoS One. 2014 Dec 15;9(12):e115016. doi: 10.1371/journal.pone.0115016 (PMC4266615; doi:10.1371/journal.pone.0115016)
Supplement: S1 File — S1–S39 Tables. Length and count data for influenza A putative ORFs accompanying Figs. 1a and 1b , and S1–S37 Figures. ORFs are listed according to their start codon positions in nt in their corresponding transcript. For an ORF of various lengths, multiple entries are displayed, ordered by the sequence counts per ORF length. For example, in S1 Table, ORFs starting at position 5 are grouped into 4 rows containing 19 148 sequences of 8 aa, 16 sequences of 16 aa, 13 sequences of 5 aa and 3 sequences of 3 aa in length. S40 Table. An abridged ORF list of influenza HA and NA genes. The filtering criteria used in Table 2, strong Kozak consensus sequence, an upstream AUG location within the upstream third of the transcript and high prevalence (≥95% and ≥50% for in-frame and alternative-frame ORFs, respectively), were applied to obtain the most probable HA and NA ORF candidates from Figs. S9–S37 (S11–S39 Tables). Because some of the genes have multiple frame-2 or frame-3 ORFs, we did not name the ORFs according to Table 2. Instead, their starting positions are provided for identification purposes. S41 Table. HHblits hits against PDB database for alternative-frame ORFs. This table supplements Table 4 for the hits of structural homologues to all frame-2 and -3 influenza A novel ORFs. E-value, sequence identity and probability were provided by averaging ones from HHblits hits. The alignments between putative ORFs and structures were generated by HHblits tool. The number in parenthesis following a sequence alignment is the length of either query or subject. Consensus sequences are shown in HMM format for both the query and the subject, in which capital and lowercase letters are used to represent conserved columns with ≥60 and ≥40 probabilities, respectively, and a tilde “∼” is used to represent non-conserved column. Based on default settings with two iterations, HHblits tool adds significant hit(s) from the previous search/iteration to the HMM query for the next search/iteration. [file pone.0115016.s001.pdf]

Tables S1–S39. Length and count data for influenza A putative ORFs accompanying Figs. 1a and 1b, and Supporting Figures S1–S37. ORFs are listed according to their start codon positions in nt in their corresponding transcript. For an ORF of various lengths, multiple entries are displayed, ordered by the sequence counts per ORF length. For example, in Table S1, ORFs starting at position 5 are grouped into 4 rows containing 19 148 sequences of 8 aa, 16 sequences of 16 aa, 13 sequences of 5 aa and 3 sequences of 3 aa in length.

Tables S1. Length and count data for influenza A putative ORFs accompanying Fig. 1a (PB1).

Position	Kozak	Frame	Length	Count
1	W	1	757	19378
5	W	2	8	19148
5	W	2	16	16
5	W	2	5	13
5	W	2	3	3
47	M	2	2	19260
47	M	2	106	12
47	M	2	73	7
47	M	2	95	6
47	S	2	2	3
47	W	2	2	2
47	M	2	50	1
95	S	2	90	12241
95	S	2	11	4730
95	S	2	57	1421
95	S	2	87	223
95	S	2	79	217
95	S	2	34	155
95	S	2	81	71
95	S	2	25	65
95	S	2	101	48
95	S	2	37	44
95	S	2	8	39
95	S	2	63	30
95	S	2	24	18
95	S	2	43	8
95	S	2	27	6
95	S	2	78	6
95	S	2	59	5
95	S	2	76	4
95	S	2	60	2
95	S	2	71	1
95	S	2	18	1
95	S	2	56	1
95	S	2	72	1
95	S	2	28	1
95	S	2	13	1
118	S	1	718	19314
118	M	1	718	4

162	S	3	12	3912
162	S	3	15	3617
162	S	3	5	478
162	S	3	20	160
162	S	3	7	139
162	S	3	23	54
162	S	3	3	28
162	S	3	2	24
162	S	3	22	8
162	M	3	5	5
162	M	3	12	4
162	S	3	35	4
162	M	3	15	2
209	M	2	52	11140
209	M	2	19	5835
209	M	2	49	235
209	M	2	41	222
209	M	2	43	71
209	M	2	63	40
209	M	2	25	39
209	M	2	5	8
209	M	2	40	6
209	M	2	21	5
209	M	2	22	4
209	M	2	38	4
209	M	2	34	1
209	M	2	3	1
209	S	2	52	1
209	M	2	18	1
209	M	2	33	1
230	M	2	45	11508
230	M	2	12	5758
230	M	2	42	249
230	M	2	34	219
230	M	2	36	70
230	M	2	56	44
230	M	2	18	38
230	M	2	15	5
230	M	2	14	5
230	M	2	31	4
230	M	2	33	2
230	S	2	45	2
230	M	2	11	1

230	M	2	26	1
245	M	2	40	12169
245	M	2	7	5840
245	M	2	37	242
245	M	2	29	141
245	M	2	51	48
245	M	2	13	38
245	M	2	31	11
245	M	2	28	6
245	M	2	10	5
245	M	2	9	5
245	M	2	26	3
245	S	2	40	2
245	M	2	6	1
245	S	2	7	1
245	M	2	22	1
245	M	2	21	1
274	S	1	666	19358
274	M	1	666	10
318	W	3	2	10107
318	W	3	14	70
318	W	3	23	38
318	W	3	28	4
331	S	1	647	19080
331	M	1	647	2
386	W	2	4	18284
386	W	2	18	6
386	W	2	14	2
458	M	2	2	8331
458	W	2	2	267
458	M	2	10	206
458	M	2	11	55
458	M	2	3	25
458	M	2	14	1
473	W	2	5	18308
473	W	2	6	114
473	M	2	5	48
473	W	2	12	6
473	W	2	9	1
506	M	2	12	505
506	M	2	13	270
506	M	2	5	159
506	M	2	10	21

506	M	2	22	17
506	M	2	2	13
506	S	2	12	1
511	S	1	587	18821
511	M	1	587	32
520	M	1	584	14183
520	W	1	584	4999
520	S	1	584	2
535	S	1	579	11147
535	M	1	579	8
583	M	1	563	17822
583	W	1	563	5
595	S	1	559	18810
595	M	1	559	554
679	M	1	531	19371
679	S	1	531	3
679	W	1	531	2
689	M	2	6	14317
689	M	2	28	8
689	S	2	6	3
689	M	2	25	1
689	M	2	18	1
689	W	2	6	1
736	M	1	512	19373
827	M	2	10	16297
827	M	2	6	1513
827	M	2	13	505
827	M	2	9	156
827	M	2	7	2
827	S	2	10	1
851	W	2	2	16555
851	W	2	5	498
851	M	2	2	4
865	M	1	469	19374
865	S	1	469	2
865	W	1	469	1
868	M	1	468	19340
868	S	1	468	28
924	M	3	2	8328
924	S	3	7	1254
924	M	3	7	743
924	S	3	2	418
924	M	3	22	102

924	M	3	25	48
924	M	3	49	25
924	M	3	38	19
924	M	3	41	2
924	S	3	22	1
924	M	3	51	1
924	S	3	41	1
924	M	3	56	1
929	M	2	11	15757
929	M	2	9	1382
929	M	2	12	9
929	M	2	27	9
929	M	2	10	3
929	W	2	9	1
929	M	2	7	1
949	W	1	441	10936
949	M	1	441	7643
961	M	1	437	19341
961	S	1	437	27
961	W	1	437	4
993	M	3	2	5707
993	M	3	21	1230
993	S	3	28	1168
993	M	3	28	717
993	M	3	15	354
993	S	3	2	290
993	M	3	18	289
993	M	3	26	174
993	S	3	15	140
993	M	3	45	136
993	S	3	45	57
993	M	3	42	38
993	S	3	26	34
993	M	3	39	8
993	S	3	18	8
993	M	3	14	4
993	S	3	21	3
993	M	3	33	1
993	M	3	51	1
993	M	3	6	1
1004	M	2	2	8047
1004	M	2	7	1
1004	M	2	4	1

1004	W	2	2	1
1015	S	1	419	5152
1027	M	1	415	19368
1027	W	1	415	5
1042	S	1	410	19288
1042	M	1	410	5
1066	W	1	402	19373
1066	M	1	402	1
1084	M	1	396	19364
1084	W	1	396	1
1114	M	1	386	19114
1114	W	1	386	1
1145	W	2	10	9941
1145	W	2	7	4780
1145	W	2	14	1719
1145	W	2	2	1202
1145	W	2	21	103
1145	M	2	10	9
1145	W	2	5	5
1145	M	2	7	2
1145	M	2	14	2
1145	M	2	2	2
1145	W	2	15	2
1145	W	2	8	1
1145	W	2	25	1
1145	W	2	9	1
1193	M	2	5	17728
1193	W	2	5	9
1193	M	2	9	2
1193	S	2	5	2
1219	M	1	351	19370
1219	W	1	351	1
1222	M	1	350	19372
1222	W	1	350	1
1225	S	1	349	19372
1225	M	1	349	2
1231	M	1	347	19373
1240	M	1	344	19371
1240	W	1	344	1
1316	S	2	11	9995
1316	S	2	10	1589
1316	S	2	12	42
1316	S	2	24	2

1334	W	2	5	7788
1334	W	2	4	1937
1334	W	2	6	1294
1334	W	2	18	36
1334	W	2	27	6
1355	W	2	20	9164
1355	W	2	11	5806
1355	W	2	25	3254
1355	W	2	2	36
1355	W	2	23	27
1355	W	2	21	16
1355	W	2	5	5
1355	W	2	6	4
1355	W	2	7	2
1355	W	2	16	1
1355	W	2	32	1
1367	M	2	16	9440
1367	M	2	7	5888
1367	M	2	21	3698
1367	M	2	19	29
1367	S	2	7	18
1367	M	2	17	16
1367	S	2	16	11
1367	S	2	21	6
1367	M	2	2	4
1367	M	2	3	2
1367	M	2	28	1
1367	M	2	12	1
1367	W	2	7	1
1429	M	1	281	19369
1429	W	1	281	1
1496	S	2	3	8180
1496	S	2	18	7973
1496	S	2	24	2533
1496	S	2	8	5
1496	S	2	12	1
1496	S	2	10	1
1496	S	2	6	1
1496	M	2	3	1
1496	S	2	30	1
1519	S	1	251	19366
1519	M	1	251	1
1553	W	2	5	15657

1553	W	2	9	14
1553	W	2	11	3
1553	M	2	5	1
1567	M	1	235	19340
1567	S	1	235	14
1600	M	1	224	19322
1600	S	1	224	50
1600	W	1	224	2
1610	M	2	39	16157
1610	M	2	15	1002
1610	M	2	74	198
1610	M	2	2	57
1610	M	2	30	10
1610	M	2	32	6
1610	M	2	53	3
1610	M	2	50	2
1610	M	2	9	2
1610	M	2	31	2
1610	M	2	26	1
1610	S	2	39	1
1610	M	2	37	1
1636	M	1	212	19366
1636	W	1	212	5
1636	S	1	212	1
1680	W	3	4	2227
1680	W	3	2	1231
1680	W	3	47	1084
1680	W	3	14	287
1680	W	3	65	211
1680	W	3	57	165
1680	W	3	39	33
1680	W	3	10	31
1680	W	3	32	10
1680	W	3	97	10
1680	W	3	74	8
1680	W	3	36	7
1680	W	3	16	5
1680	W	3	41	2
1737	S	3	13	1182
1737	S	3	28	435
1737	S	3	46	277
1737	S	3	20	251
1737	S	3	38	245

1737	S	3	6	145
1737	S	3	32	49
1737	S	3	55	20
1737	S	3	43	12
1737	S	3	17	6
1737	S	3	78	4
1737	S	3	22	4
1737	S	3	41	3
1737	S	3	58	2
1737	M	3	13	2
1737	S	3	53	1
1778	M	2	18	18609
1778	M	2	22	53
1778	M	2	8	19
1778	M	2	3	1
1778	M	2	5	1
1836	S	3	5	9852
1836	S	3	13	2521
1836	S	3	45	355
1836	S	3	22	58
1836	M	3	22	47
1836	S	3	8	33
1836	S	3	25	29
1836	S	3	6	9
1836	S	3	29	7
1836	S	3	10	4
1836	S	3	16	1
1836	S	3	20	1
1836	M	3	13	1
1836	M	3	5	1
1836	S	3	56	1
1846	M	1	142	19373
1846	S	1	142	4
1850	W	2	11	14581
1850	W	2	23	75
1850	M	2	11	24
1850	W	2	27	5
1850	W	2	20	2
1850	W	2	28	1
1850	W	2	7	1
1853	M	2	10	4455
1853	S	2	10	4
1853	M	2	22	2

1853	M	2	4	1
1872	W	3	10	1310
1872	M	3	13	138
1872	M	3	8	74
1872	M	3	10	30
1872	W	3	13	3
1872	M	3	4	1
1872	W	3	8	1
1925	M	2	3	10918
1925	M	2	2	6548
1925	M	2	102	50
1925	M	2	18	2
1925	W	2	3	2
1925	W	2	2	1
1936	M	1	112	19267
1936	W	1	112	2
1946	M	2	95	13649
1946	M	2	26	3724
1946	M	2	66	622
1946	M	2	11	542
1946	M	2	69	100
1946	M	2	86	37
1946	M	2	6	32
1946	M	2	65	31
1946	M	2	79	26
1946	M	2	34	18
1946	M	2	60	14
1946	M	2	38	3
1946	M	2	107	2
1946	M	2	94	2
1946	M	2	87	2
1946	M	2	35	1
1946	M	2	48	1
1946	M	2	4	1
1946	M	2	74	1
1946	M	2	3	1
1963	S	1	103	19259
1963	M	1	103	5
1970	M	2	87	13959
1970	M	2	18	3602
1970	M	2	58	555
1970	M	2	3	538
1970	M	2	61	89

1970	M	2	78	37
1970	M	2	57	33
1970	M	2	71	26
1970	M	2	26	19
1970	M	2	52	14
1970	M	2	30	3
1970	M	2	99	2
1970	M	2	86	2
1970	M	2	79	2
1970	M	2	12	1
1970	M	2	27	1
1970	M	2	40	1
1970	S	2	87	1
1970	M	2	66	1
1973	M	2	86	13702
1973	M	2	17	2284
1973	M	2	57	631
1973	M	2	2	537
1973	M	2	60	93
1973	M	2	77	37
1973	M	2	56	33
1973	M	2	25	19
1973	M	2	51	14
1973	M	2	70	11
1973	M	2	29	3
1973	M	2	98	2
1973	M	2	78	2
1973	M	2	26	1
1973	M	2	39	1
1973	W	2	86	1
1973	M	2	65	1
1973	S	2	86	1
1973	M	2	85	1
1995	M	3	3	2827
1995	M	3	18	220
1995	M	3	20	47
1995	M	3	12	7
1995	S	3	3	1
1995	M	3	36	1
1995	M	3	10	1
2054	M	2	59	16134
2054	M	2	30	1884
2054	M	2	50	268

2054	M	2	33	110
2054	M	2	29	41
2054	M	2	43	25
2054	M	2	24	13
2054	M	2	2	3
2054	M	2	5	2
2054	M	2	58	2
2054	M	2	51	2
2054	S	2	59	2
2054	M	2	71	2
2054	W	2	30	1
2054	M	2	12	1
2054	M	2	38	1
2062	W	1	70	19332
2062	M	1	70	19
2073	M	3	10	717
2073	M	3	5	480
2073	M	3	2	231
2073	M	3	11	178
2073	M	3	29	41
2073	M	3	21	2
2073	M	3	33	1
2140	S	1	44	19325
2140	M	1	44	40
2152	S	1	40	19074
2152	M	1	40	292
2174	W	2	19	15810
2174	W	2	10	1788
2174	W	2	3	26
2174	W	2	31	2
2174	W	2	18	2
2174	W	2	11	1
2230	M	1	14	18862
2230	S	1	14	2
2230	W	1	14	1

Table S2. Length and count data for influenza A putative ORFs accompanying Fig. 1b (PA).

Position	Kozak	Frame	Length	Count
1	W	1	716	20404
21	M	3	25	6616
21	M	3	47	5055
21	M	3	23	4598
21	M	3	48	2225
21	M	3	42	264
21	M	3	2	203
21	M	3	26	196
21	M	3	7	129
21	W	3	47	90
21	M	3	59	75
21	M	3	21	66
21	M	3	51	48
21	M	3	10	21
21	M	3	69	6
21	M	3	61	2
21	M	3	11	2
21	S	3	47	2
21	W	3	23	1
21	M	3	14	1
21	M	3	58	1
21	M	3	4	1
21	M	3	17	1
21	M	3	18	1
21	W	3	42	1
21	S	3	48	1
34	W	1	705	20248
34	M	1	705	134
61	M	1	696	20388
61	W	1	696	10
61	S	1	696	1
71	S	2	37	10512
71	S	2	4	5099
71	S	2	18	3187
71	S	2	39	747
71	S	2	30	276
71	S	2	6	100
71	S	2	38	27
71	S	2	48	26

71	S	2	8	8
71	S	2	11	5
71	S	2	7	2
71	S	2	14	1
71	M	2	37	1
71	S	2	33	1
114	M	3	16	8060
114	M	3	17	6186
114	M	3	20	5116
114	M	3	11	538
114	M	3	28	306
114	W	3	16	58
114	M	3	4	33
114	W	3	17	31
114	M	3	27	8
114	M	3	38	7
114	M	3	30	2
114	W	3	20	1
114	M	3	13	1
132	M	3	11	3420
132	M	3	10	951
132	M	3	22	197
132	M	3	5	32
132	M	3	14	26
132	M	3	32	5
132	M	3	21	4
139	W	1	670	20401
164	W	2	6	12139
164	W	2	8	229
164	W	2	7	30
164	W	2	17	19
164	W	2	2	10
164	M	2	6	2
164	W	2	5	1
197	W	2	6	2394
197	M	2	6	1
197	W	2	12	1
206	W	2	3	19445
206	W	2	9	58
206	M	2	3	26
206	W	2	2	13
256	S	1	631	20081
256	M	1	631	9

261	S	3	13	1386
261	S	3	5	394
261	S	3	8	123
261	S	3	23	30
261	S	3	53	17
261	S	3	16	15
261	S	3	28	9
261	S	3	25	5
261	S	3	31	3
261	S	3	4	2
261	S	3	14	1
261	M	3	5	1
261	S	3	27	1
329	W	2	12	17094
329	W	2	10	120
329	W	2	22	84
329	W	2	8	64
329	W	2	28	7
329	W	2	68	3
329	M	2	12	2
463	S	1	562	20379
463	M	1	562	6
491	W	2	14	9084
491	W	2	7	924
491	W	2	89	67
491	W	2	69	7
491	M	2	7	1
491	M	2	14	1
544	S	1	535	20312
544	M	1	535	8
561	M	3	14	12270
561	M	3	7	150
561	S	3	14	146
561	S	3	7	26
561	M	3	10	23
561	M	3	18	21
561	M	3	39	5
561	M	3	5	5
561	S	3	18	1
561	W	3	14	1
561	M	3	11	1
561	M	3	4	1
631	M	1	506	20310

631	W	1	506	4
695	W	2	21	14797
695	W	2	29	18
695	W	2	10	7
695	W	2	6	3
695	W	2	17	2
695	W	2	16	2
695	W	2	4	1
695	W	2	18	1
695	M	2	21	1
701	M	2	19	20285
701	M	2	27	42
701	M	2	8	7
701	M	2	18	4
701	M	2	4	3
701	M	2	15	3
701	M	2	14	2
701	M	2	16	1
701	S	2	19	1
701	M	2	2	1
745	W	1	468	20383
745	M	1	468	6
761	W	2	7	9206
761	W	2	30	237
761	W	2	3	57
761	W	2	2	6
761	W	2	14	3
761	M	2	7	2
815	M	2	12	17329
815	M	2	18	41
815	M	2	16	40
815	M	2	11	12
815	M	2	39	10
815	S	2	12	4
815	W	2	12	2
815	M	2	13	1
815	M	2	8	1
853	M	1	432	20374
853	S	1	432	18
853	W	1	432	2
857	W	2	2	16268
857	W	2	4	2655
857	W	2	25	18

857	M	2	2	5
857	M	2	4	1
890	M	2	14	9852
890	M	2	11	109
890	W	2	14	12
890	M	2	25	6
890	M	2	33	2
890	S	2	14	1
914	W	2	6	16740
914	W	2	3	119
914	W	2	17	3
914	W	2	25	2
917	M	2	5	19878
917	M	2	2	124
917	M	2	16	10
917	W	2	5	6
917	M	2	24	2
927	W	3	11	7460
927	W	3	17	6025
927	W	3	14	567
927	W	3	39	231
927	M	3	11	39
927	M	3	17	38
927	M	3	39	19
927	M	3	14	13
927	W	3	41	8
927	W	3	2	6
927	W	3	23	2
927	W	3	21	2
927	M	3	18	1
927	W	3	6	1
927	M	3	23	1
927	W	3	13	1
931	W	1	406	17009
931	M	1	406	1
948	M	3	32	4423
948	M	3	10	794
948	M	3	7	447
948	M	3	16	187
948	M	3	4	91
948	M	3	30	31
948	M	3	11	7
948	M	3	34	5

948	M	3	27	3
948	M	3	14	1
948	M	3	6	1
977	W	2	4	14651
977	M	2	4	182
977	W	2	34	137
977	W	2	16	116
977	W	2	10	30
977	M	2	10	7
977	W	2	2	1
1006	M	1	381	4599
1006	W	1	381	71
1011	S	3	11	5123
1011	S	3	13	34
1011	S	3	9	33
1011	S	3	6	6
1011	M	3	11	5
1049	M	2	10	16833
1049	M	2	7	59
1049	S	2	10	40
1049	M	2	5	11
1049	W	2	10	3
1049	M	2	16	3
1049	M	2	2	2
1049	M	2	3	1
1049	M	2	6	1
1078	M	1	357	20286
1078	W	1	357	5
1078	S	1	357	3
1101	S	3	4	1475
1101	S	3	14	31
1101	S	3	20	2
1101	S	3	9	1
1101	S	3	3	1
1120	S	1	343	20335
1145	W	2	21	5882
1145	W	2	5	1134
1145	W	2	8	358
1145	W	2	35	91
1145	M	2	21	3
1145	M	2	8	1
1157	M	2	4	6259
1157	M	2	17	5936

1157	M	2	31	84
1157	W	2	4	1
1157	S	2	17	1
1178	M	2	10	11732
1178	M	2	7	5356
1178	M	2	24	2261
1178	S	2	10	52
1178	S	2	24	5
1178	M	2	30	2
1178	M	2	11	1
1178	S	2	7	1
1187	M	2	7	12154
1187	M	2	21	2300
1187	M	2	4	435
1187	S	2	4	11
1187	S	2	7	6
1187	W	2	21	3
1187	M	2	27	2
1187	S	2	21	1
1187	M	2	8	1
1226	M	2	8	11248
1226	M	2	14	10
1226	W	2	8	1
1226	M	2	2	1
1226	S	2	8	1
1242	M	3	9	1643
1242	M	3	12	824
1242	M	3	4	660
1242	M	3	11	650
1242	M	3	21	7
1277	W	2	2	13661
1277	W	2	6	3449
1277	W	2	15	2627
1277	W	2	9	142
1277	W	2	33	20
1277	W	2	4	10
1277	M	2	2	2
1292	M	2	10	12753
1292	M	2	4	150
1292	M	2	28	78
1292	M	2	7	46
1292	W	2	10	14
1321	M	1	276	20197

1321	W	1	276	3
1378	M	1	257	20296
1378	W	1	257	15
1412	W	2	11	18605
1412	W	2	14	53
1412	W	2	6	17
1412	W	2	4	4
1412	M	2	11	3
1412	W	2	5	1
1412	W	2	9	1
1429	S	1	240	20365
1429	M	1	240	34
1433	W	2	4	16352
1433	W	2	7	57
1433	M	2	4	5
1433	W	2	2	1
1453	W	1	232	20373
1453	M	1	232	8
1464	W	3	25	2476
1464	W	3	10	1332
1464	W	3	27	1094
1464	W	3	40	185
1464	W	3	17	161
1464	W	3	3	96
1464	W	3	6	32
1464	W	3	55	24
1464	W	3	8	9
1464	W	3	19	2
1464	S	3	25	1
1464	W	3	30	1
1502	M	2	4	17276
1502	M	2	3	889
1502	M	2	10	80
1502	M	2	2	1
1538	M	2	5	17537
1538	M	2	4	928
1538	M	2	8	122
1538	S	2	5	1
1538	M	2	19	1
1547	W	2	2	13483
1547	W	2	5	43
1547	W	2	16	1
1567	S	1	194	20398

1608	M	3	7	15900
1608	M	3	56	848
1608	M	3	29	194
1608	M	3	35	150
1608	M	3	37	142
1608	M	3	31	16
1608	M	3	60	13
1608	S	3	7	11
1608	M	3	2	4
1608	M	3	62	4
1608	M	3	4	4
1608	S	3	56	2
1608	M	3	16	1
1642	M	1	169	20080
1642	S	1	169	253
1642	W	1	169	1
1681	W	1	156	20224
1681	M	1	156	33
1691	W	2	11	1485
1691	W	2	9	15
1703	M	2	7	14961
1703	M	2	5	278
1703	M	2	13	1
1703	W	2	7	1
1723	M	1	142	20393
1723	W	1	142	1
1728	S	3	16	13953
1728	S	3	20	5557
1728	S	3	36	25
1728	S	3	22	11
1728	S	3	26	10
1728	S	3	32	9
1728	S	3	3	4
1728	M	3	16	2
1728	S	3	5	1
1735	S	1	138	20367
1735	M	1	138	4
1741	M	1	136	20096
1749	M	3	9	2489
1749	M	3	13	61
1749	M	3	15	3
1749	M	3	29	2
1749	W	3	9	1

1783	M	1	122	20247
1783	S	1	122	76
1819	M	1	110	20101
1819	S	1	110	1
1854	S	3	37	7531
1854	S	3	19	4082
1854	S	3	42	2864
1854	S	3	24	1264
1854	S	3	7	755
1854	S	3	29	592
1854	S	3	28	325
1854	S	3	4	194
1854	S	3	54	145
1854	S	3	10	54
1854	S	3	16	25
1854	S	3	44	4
1854	S	3	69	2
1854	M	3	42	1
1854	M	3	29	1
1854	S	3	56	1
1854	M	3	19	1
1908	M	3	19	2206
1908	M	3	6	1084
1908	M	3	11	384
1908	M	3	36	316
1908	M	3	24	239
1908	M	3	10	80
1908	M	3	26	5
1949	W	2	44	13854
1949	W	2	5	3126
1949	W	2	17	278
1949	W	2	26	48
1949	W	2	65	31
1949	W	2	18	13
1949	W	2	22	12
1949	W	2	38	10
1949	W	2	40	7
1949	W	2	33	3
1949	W	2	61	2
1949	W	2	15	2
1949	W	2	9	1
1949	W	2	12	1
2060	W	2	7	18615

2060	M	2	7	151
2060	W	2	28	63
2060	W	2	3	13
2060	W	2	24	5
2060	M	2	3	1
2087	W	2	19	12656
2087	W	2	15	5069
2087	W	2	6	39
2087	W	2	11	27
2087	W	2	5	2
2108	W	2	12	14400
2108	W	2	8	5112
2108	W	2	4	27
2138	W	2	2	19673
2138	M	2	2	85

Table S3. Length and count data for influenza A putative ORFs accompanying Figure S1 (PB2).

Position	Kozak	Frame	Length	Count
1	W	1	759	19320
31	W	1	749	19305
31	M	1	749	8
82	M	1	732	19283
82	W	1	732	15
82	S	1	732	2
139	M	1	713	19289
139	W	1	713	16
144	S	3	11	5239
144	S	3	20	3277
144	S	3	12	1422
144	S	3	23	467
144	S	3	16	218
144	S	3	34	6
144	S	3	31	2
144	M	3	12	1
148	W	1	710	19270
148	M	1	710	2
151	S	1	709	19312
151	M	1	709	5
157	M	1	707	19305
157	W	1	707	2
190	S	1	696	12629
190	M	1	696	79
196	M	1	694	14818
196	S	1	694	3875
196	W	1	694	1
212	M	2	18	13722
212	M	2	10	4704
212	M	2	25	55
212	M	2	23	32
212	S	2	10	2
212	M	2	9	2
212	M	2	19	1
212	M	2	6	1
212	M	2	15	1
212	W	2	18	1
231	M	3	2	3825
231	S	3	2	1253

231	M	3	23	153
231	M	3	5	153
231	M	3	38	119
231	S	3	5	13
231	M	3	35	11
231	S	3	38	4
231	M	3	10	3
231	S	3	23	3
231	S	3	42	2
231	S	3	45	1
231	M	3	25	1
241	M	1	679	4179
241	S	1	679	1
241	W	1	679	1
245	W	2	7	7299
245	W	2	14	31
245	W	2	12	14
245	W	2	8	2
245	W	2	4	1
245	M	2	7	1
248	M	2	6	18618
248	W	2	6	163
248	M	2	13	63
248	M	2	11	35
248	M	2	7	3
248	M	2	3	1
268	S	1	670	18312
268	M	1	670	897
291	S	3	3	14099
291	S	3	22	1276
291	S	3	18	118
291	M	3	18	101
291	M	3	3	29
291	S	3	9	28
291	S	3	15	25
291	S	3	25	4
291	S	3	38	2
291	M	3	15	1
291	M	3	5	1
291	M	3	22	1
305	S	2	23	14760
305	S	2	3	3851
305	S	2	20	59

305	S	2	6	17
305	S	2	21	11
305	S	2	2	8
305	S	2	5	4
305	S	2	4	2
305	M	2	23	2
305	S	2	10	2
305	S	2	18	1
305	S	2	39	1
305	S	2	17	1
305	S	2	75	1
380	S	2	14	6602
380	S	2	20	3793
380	S	2	18	3578
380	S	2	50	591
380	S	2	36	98
380	S	2	12	39
380	S	2	39	20
380	S	2	35	8
380	S	2	45	4
380	S	2	49	2
380	S	2	17	2
380	S	2	27	1
380	M	2	20	1
380	S	2	8	1
380	S	2	25	1
380	S	2	56	1
380	M	2	14	1
452	M	2	11	12093
452	M	2	26	2979
452	M	2	12	387
452	M	2	21	111
452	M	2	15	95
452	M	2	3	85
452	M	2	32	16
452	M	2	25	4
452	W	2	26	4
452	M	2	9	2
482	M	2	16	3163
482	M	2	2	389
482	M	2	11	182
482	M	2	5	95
482	M	2	22	18

482	M	2	15	2
482	W	2	16	1
482	S	2	5	1
490	S	1	596	19309
490	M	1	596	4
509	W	2	7	16552
509	W	2	2	1181
509	W	2	13	138
509	W	2	6	24
509	W	2	15	10
509	M	2	7	2
509	W	2	5	2
509	M	2	2	1
604	M	1	558	19193
604	W	1	558	79
604	S	1	558	2
616	W	1	554	19303
616	M	1	554	3
714	M	3	14	6046
714	M	3	15	659
714	M	3	12	88
714	W	3	15	85
714	M	3	10	26
714	W	3	14	14
714	M	3	17	5
714	M	3	81	2
727	W	1	517	18991
727	M	1	517	1
755	M	2	7	17284
755	M	2	3	555
755	M	2	14	25
755	M	2	40	22
755	W	2	7	17
755	M	2	29	16
755	M	2	25	3
755	M	2	15	2
755	M	2	43	1
755	W	2	14	1
755	M	2	56	1
755	M	2	41	1
755	S	2	15	1
758	M	2	6	12848
758	M	2	2	1128

758	M	2	13	18
758	M	2	28	16
758	M	2	24	3
758	M	2	14	2
758	M	2	39	1
761	M	2	5	16449
761	M	2	12	23
761	M	2	38	22
761	M	2	27	16
761	M	2	13	3
761	M	2	23	3
761	S	2	5	1
761	M	2	41	1
761	M	2	54	1
761	M	2	39	1
847	M	1	477	19297
847	W	1	477	8
880	S	1	466	18516
880	M	1	466	798
880	W	1	466	1
930	W	3	9	9581
930	W	3	20	4586
930	W	3	47	2199
930	W	3	13	2006
930	W	3	50	139
930	W	3	28	108
930	M	3	9	44
930	W	3	7	36
930	M	3	13	25
930	W	3	37	19
930	W	3	44	5
930	W	3	23	4
930	M	3	20	3
930	W	3	27	2
930	W	3	16	1
930	W	3	30	1
930	W	3	31	1
943	S	1	445	14609
943	M	1	445	8
1070	W	2	24	11192
1070	W	2	17	4641
1070	W	2	28	2195
1070	W	2	27	340

1070	W	2	9	179
1070	W	2	8	159
1070	W	2	6	24
1070	W	2	16	13
1070	W	2	20	5
1070	W	2	7	4
1070	W	2	11	4
1070	M	2	28	3
1070	W	2	22	3
1070	M	2	24	3
1070	W	2	13	1
1070	W	2	18	1
1070	W	2	14	1
1079	M	2	21	11048
1079	M	2	14	4760
1079	M	2	25	1656
1079	M	2	6	165
1079	M	2	5	160
1079	M	2	24	148
1079	M	2	3	23
1079	M	2	4	6
1079	M	2	17	4
1079	M	2	8	4
1079	M	2	19	3
1079	M	2	13	2
1079	M	2	11	1
1079	W	2	21	1
1079	M	2	10	1
1093	S	1	395	19034
1093	M	1	395	37
1169	M	2	8	1409
1169	M	2	10	646
1169	M	2	4	346
1169	M	2	9	116
1169	M	2	20	65
1169	M	2	7	19
1169	S	2	10	2
1204	S	1	358	19252
1204	M	1	358	18
1228	W	1	350	18448
1228	M	1	350	830
1228	S	1	350	2
1291	W	1	329	19195

1291	M	1	329	122
1322	M	2	16	14223
1322	M	2	19	1868
1322	M	2	6	1090
1322	M	2	13	342
1322	M	2	4	169
1322	M	2	26	51
1322	M	2	10	12
1322	M	2	18	1
1322	M	2	20	1
1367	M	2	4	3046
1367	M	2	11	57
1367	M	2	5	12
1367	S	2	3	1
1372	S	1	302	19316
1372	M	1	302	2
1378	M	1	300	16277
1378	S	1	300	3029
1399	M	1	293	18938
1399	S	1	293	58
1399	W	1	293	1
1417	M	1	287	18095
1417	W	1	287	2
1423	W	1	285	5432
1447	S	1	277	18861
1447	M	1	277	4
1457	W	2	9	6806
1457	W	2	8	4898
1457	W	2	16	3576
1457	W	2	10	3290
1457	M	2	10	5
1457	M	2	8	4
1457	W	2	5	3
1457	M	2	9	1
1457	W	2	41	1
1469	W	2	4	1153
1469	W	2	6	24
1529	M	2	17	8178
1529	M	2	8	451
1529	M	2	19	60
1529	M	2	14	38
1529	M	2	12	4
1529	M	2	3	1

1603	W	1	225	18920
1603	M	1	225	5
1606	M	1	224	18920
1606	W	1	224	393
1619	M	2	6	6612
1619	M	2	20	3699
1619	M	2	14	714
1619	M	2	13	710
1619	M	2	43	585
1619	M	2	7	284
1619	M	2	56	68
1619	M	2	35	7
1619	M	2	48	2
1619	M	2	19	2
1619	S	2	13	1
1619	M	2	15	1
1619	W	2	43	1
1653	M	3	26	8379
1653	M	3	22	1725
1653	M	3	3	1561
1653	M	3	9	374
1653	M	3	21	268
1653	M	3	34	99
1653	M	3	24	3
1653	M	3	15	1
1653	W	3	26	1
1689	M	3	14	8962
1689	M	3	10	3864
1689	M	3	9	3544
1689	M	3	22	223
1689	S	3	10	88
1689	M	3	3	8
1689	M	3	2	4
1689	S	3	14	4
1689	M	3	12	3
1689	S	3	22	3
1689	W	3	9	1
1708	M	1	190	18672
1708	W	1	190	117
1723	S	1	185	18786
1723	M	1	185	1
1807	W	1	157	19315
1814	M	2	10	15074

1814	M	2	2	175
1814	M	2	11	58
1814	M	2	3	2
1891	M	1	129	19256
1891	W	1	129	41
1933	M	1	115	14156
2012	M	2	4	16543
2012	M	2	20	437
2012	M	2	15	335
2012	M	2	5	11
2012	S	2	4	3
2012	M	2	24	2
2012	W	2	4	2
2012	M	2	12	2
2012	M	2	13	1
2012	M	2	8	1
2039	W	2	11	15206
2039	W	2	6	3088
2039	W	2	3	25
2039	W	2	28	17
2039	W	2	15	6
2039	M	2	11	5
2039	M	2	6	4
2039	W	2	4	2
2039	W	2	17	2
2039	W	2	10	1
2111	S	2	4	15284
2111	S	2	9	27
2111	M	2	2	3
2111	M	2	4	3
2132	W	2	2	17677
2132	W	2	14	119
2132	M	2	2	1
2132	W	2	15	1
2168	W	2	2	18621
2168	W	2	3	130
2168	M	2	2	6
2168	W	2	8	1
2203	M	1	25	19315
2266	M	1	4	19216
2266	W	1	4	3
2266	S	1	4	2

Table S4. Length and count data for influenza A putative ORFs accompanying Figure S2 (NP).

Position	Kozak	Frame	Length	Count
1	W	1	498	20219
29	W	2	22	10273
29	W	2	46	7764
29	W	2	51	225
29	W	2	39	155
29	W	2	37	83
29	W	2	53	82
29	W	2	26	81
29	W	2	19	53
29	W	2	23	44
29	W	2	15	34
29	W	2	95	4
29	M	2	46	3
29	W	2	2	2
29	W	2	12	1
29	W	2	33	1
29	W	2	13	1
37	M	1	486	20199
37	S	1	486	15
47	M	2	16	5276
47	M	2	33	106
47	M	2	13	34
47	M	2	40	30
47	M	2	45	1
47	M	2	27	1
62	M	2	11	11394
62	M	2	35	7771
62	M	2	40	222
62	M	2	28	155
62	M	2	42	86
62	M	2	15	80
62	M	2	26	77
62	M	2	8	68
62	M	2	12	43
62	M	2	4	33
62	W	2	11	16
62	W	2	35	9
62	M	2	84	4
62	M	2	22	1

62	W	2	40	1
62	M	2	2	1
94	M	1	467	11598
94	S	1	467	8617
94	W	1	467	2
101	M	2	22	4010
101	M	2	27	44
101	M	2	15	11
101	M	2	13	2
127	W	1	456	20200
149	W	2	6	1345
149	W	2	11	69
149	W	2	13	39
155	M	2	4	17333
155	M	2	9	492
155	M	2	11	90
155	M	2	53	4
155	S	2	4	1
158	S	2	3	4561
158	M	2	3	1
158	S	2	8	1
196	S	1	433	20128
196	M	1	433	88
215	W	2	33	8248
215	W	2	7	6542
215	W	2	28	3879
215	W	2	36	245
215	W	2	64	9
215	W	2	30	3
215	W	2	32	3
215	M	2	7	2
215	W	2	27	1
215	M	2	28	1
215	W	2	44	1
302	S	2	4	5423
302	M	2	4	5254
302	S	2	7	199
302	M	2	15	30
302	S	2	35	15
302	S	2	3	3
302	S	2	15	1
309	S	3	8	9986
309	S	3	41	959

309	M	3	8	865
309	S	3	21	193
309	S	3	20	139
309	S	3	2	73
309	S	3	38	26
309	S	3	11	17
309	S	3	13	17
309	S	3	9	8
309	S	3	98	6
309	S	3	116	5
309	M	3	20	4
309	S	3	23	3
309	S	3	24	1
309	S	3	376	1
309	S	3	244	1
313	W	1	394	15159
332	W	2	5	11406
332	W	2	25	6128
332	W	2	22	888
332	W	2	6	160
332	W	2	26	13
332	W	2	11	2
332	M	2	25	1
332	W	2	32	1
332	W	2	18	1
374	S	2	11	7408
374	S	2	12	5894
374	S	2	8	5891
374	M	2	11	4
374	S	2	18	2
374	S	2	4	1
374	S	2	5	1
380	M	2	6	2008
380	M	2	9	17
380	M	2	10	17
383	M	2	9	5774
383	M	2	5	5400
383	M	2	8	1770
383	W	2	5	1
383	M	2	15	1
383	M	2	2	1
406	W	1	363	2956
409	M	1	362	12199

409	W	1	362	8014
409	S	1	362	3
414	S	3	6	1689
414	S	3	3	23
414	S	3	81	6
414	S	3	5	3
414	S	3	63	1
414	S	3	341	1
414	S	3	209	1
431	W	2	22	19275
431	W	2	10	468
431	W	2	39	179
431	W	2	2	7
431	W	2	6	4
431	W	2	11	3
431	M	2	22	3
431	W	2	19	1
431	W	2	46	1
434	M	2	21	16037
434	M	2	9	426
434	M	2	38	148
434	M	2	5	4
434	M	2	10	3
434	M	2	18	1
475	S	1	340	20164
475	M	1	340	1
487	M	1	336	19920
487	W	1	336	287
487	S	1	336	1
499	W	1	332	20206
499	M	1	332	2
565	S	1	310	14286
565	M	1	310	25
571	S	1	308	18927
571	M	1	308	4
586	M	1	303	9006
586	W	1	303	8548
586	S	1	303	2660
605	W	2	36	13790
605	W	2	24	339
605	W	2	40	74
605	W	2	15	40
605	W	2	64	28

605	W	2	30	11
605	W	2	57	6
605	W	2	35	4
605	W	2	29	1
605	W	2	54	1
605	W	2	96	1
605	M	2	36	1
605	W	2	11	1
605	W	2	14	1
605	W	2	12	1
605	W	2	25	1
632	S	2	27	15994
632	S	2	31	3017
632	S	2	15	399
632	S	2	48	176
632	S	2	55	67
632	S	2	6	36
632	S	2	42	28
632	S	2	21	14
632	S	2	28	5
632	M	2	27	4
632	S	2	26	3
632	S	2	87	2
632	S	2	5	2
632	S	2	41	1
632	S	2	45	1
632	S	2	3	1
632	S	2	53	1
632	S	2	2	1
632	S	2	16	1
632	S	2	90	1
632	S	2	20	1
656	W	2	19	12816
656	W	2	23	2971
656	W	2	7	409
656	W	2	40	176
656	W	2	47	64
656	W	2	13	9
656	W	2	20	5
656	W	2	18	4
656	W	2	34	3
656	W	2	37	2
656	W	2	79	2

656	W	2	82	1
656	W	2	12	1
656	W	2	45	1
656	M	2	19	1
656	W	2	8	1
664	M	1	277	20200
664	W	1	277	5
712	M	1	261	16882
712	S	1	261	3327
712	W	1	261	2
715	S	1	260	16875
715	M	1	260	5
749	M	2	16	9750
749	M	2	14	2470
749	M	2	9	569
749	M	2	3	360
749	M	2	6	89
749	M	2	51	20
749	M	2	35	16
749	M	2	48	9
749	M	2	2	3
749	M	2	20	2
749	W	2	16	1
842	M	2	4	8702
842	M	2	20	4177
842	M	2	17	3586
842	M	2	60	398
842	M	2	47	72
842	M	2	2	51
842	M	2	18	24
842	M	2	34	15
842	M	2	3	3
842	W	2	17	1
842	S	2	4	1
866	M	2	12	8087
866	M	2	9	1905
866	M	2	10	770
866	M	2	26	208
866	M	2	52	195
866	M	2	39	44
866	M	2	24	8
866	M	2	3	3
866	W	2	12	2

866	M	2	27	1
946	W	1	183	4743
946	M	1	183	3
956	W	2	22	8660
956	W	2	9	2714
956	W	2	62	51
956	W	2	28	16
956	W	2	25	15
956	W	2	12	12
956	W	2	33	7
956	M	2	9	4
956	M	2	22	2
956	W	2	3	1
956	W	2	10	1
987	S	3	9	5997
987	S	3	39	111
987	S	3	31	20
987	M	3	9	20
987	S	3	18	17
987	S	3	12	2
987	S	3	47	1
987	S	3	15	1
987	S	3	50	1
987	M	3	25	1
991	M	1	168	20167
991	W	1	168	7
991	S	1	168	3
996	M	3	6	19129
996	M	3	36	344
996	M	3	28	58
996	M	3	9	40
996	M	3	44	37
996	M	3	15	18
996	M	3	47	13
996	W	3	6	5
996	M	3	147	2
996	M	3	12	1
996	M	3	22	1
1103	W	2	13	18108
1103	W	2	11	853
1103	W	2	6	508
1103	W	2	3	233
1103	W	2	5	161

1103	W	2	20	72
1103	W	2	9	16
1103	M	2	13	4
1103	M	2	3	1
1103	M	2	6	1
1109	M	2	11	4939
1109	M	2	9	791
1109	M	2	4	24
1109	M	2	18	2
1111	S	1	128	13839
1111	M	1	128	2
1115	W	2	9	1182
1115	W	2	2	16
1115	W	2	7	8
1115	W	2	16	1
1115	M	2	9	1
1120	S	1	125	18404
1120	M	1	125	164
1276	S	1	73	19726
1276	M	1	73	4
1298	M	2	12	5721
1298	M	2	7	143
1298	M	2	11	7
1298	M	2	14	4
1298	W	2	12	4
1298	M	2	8	1
1298	W	2	7	1
1298	S	2	12	1
1318	M	1	59	20204
1318	W	1	59	3
1318	S	1	59	2
1339	M	1	52	20087
1339	W	1	52	37
1339	S	1	52	1
1342	S	1	51	20187
1342	M	1	51	27
1349	M	2	31	5304
1349	M	2	25	5
1349	M	2	29	3
1364	M	2	26	8812
1364	M	2	20	7
1364	M	2	24	4
1364	M	2	17	2

1441	M	1	18	20106
1441	S	1	18	5
1441	W	1	18	2

Table S5. Length and count data for influenza A putative ORFs accompanying Figure S3 (M1).

Position	Kozak	Frame	Length	Count
1	W	1	252	28216
29	W	2	36	5914
29	W	2	14	1457
29	W	2	4	44
29	W	2	29	12
29	W	2	27	8
29	W	2	32	6
29	W	2	18	1
29	M	2	36	1
89	M	2	16	19316
89	M	2	9	126
89	M	2	12	124
89	M	2	7	8
89	M	2	25	6
89	M	2	3	5
89	M	2	13	4
89	M	2	54	1
89	M	2	2	1
127	M	1	210	28171
127	S	1	210	27
127	W	1	210	1
132	S	3	12	23198
132	S	3	26	314
132	S	3	4	2
132	M	3	12	2
132	S	3	2	1
245	M	2	2	12808
245	M	2	25	10779
245	M	2	21	2718
245	M	2	33	517
245	M	2	15	383
245	M	2	56	60
245	M	2	11	9
245	M	2	35	1
245	W	2	2	1
254	M	2	22	18539
254	M	2	18	8053
254	M	2	30	522
254	M	2	12	330

254	M	2	53	60
254	M	2	8	40
254	M	2	32	6
254	M	2	10	1
254	M	2	11	1
254	W	2	18	1
254	W	2	22	1
260	S	2	20	18347
260	S	2	16	8064
260	S	2	28	520
260	S	2	10	332
260	S	2	51	58
260	S	2	6	40
260	M	2	16	8
260	S	2	30	6
260	M	2	20	1
260	S	2	9	1
277	S	1	160	28161
277	M	1	160	15
329	M	2	28	9516
329	M	2	5	9341
329	M	2	7	7893
329	M	2	14	313
329	M	2	20	255
329	M	2	56	84
329	M	2	36	83
329	S	2	5	9
329	M	2	55	5
329	W	2	5	4
329	S	2	28	4
329	M	2	62	4
329	S	2	56	2
329	W	2	20	2
329	W	2	28	2
329	M	2	18	2
329	M	2	4	1
329	S	2	7	1
329	M	2	25	1
329	M	2	3	1
329	M	2	2	1
382	M	1	125	28200
382	W	1	125	3
382	S	1	125	1

403	S	1	118	27364
403	M	1	118	850
441	M	3	4	8787
441	W	3	4	1657
441	M	3	8	72
441	W	3	12	1
441	M	3	39	1
493	M	1	88	28101
493	W	1	88	84
493	S	1	88	2
524	M	2	41	21108
524	M	2	6	2951
524	M	2	5	133
524	M	2	28	47
524	M	2	17	22
524	M	2	30	16
524	M	2	37	11
524	W	2	41	3
524	M	2	36	3
524	M	2	20	2
524	M	2	25	2
524	M	2	54	2
524	S	2	41	1
524	M	2	14	1
524	M	2	10	1
524	M	2	38	1
524	M	2	24	1
535	S	1	74	28183
535	M	1	74	31
565	S	1	64	28209
565	M	1	64	1
574	M	1	61	27551
574	W	1	61	3
574	S	1	61	1
607	S	1	50	28137
607	M	1	50	14
634	M	1	41	27900
634	W	1	41	279
634	S	1	41	33
641	W	2	2	7966
646	M	1	37	28212
646	W	1	37	1
730	M	1	9	27884

730	S	1	9	320
742	W	1	5	27111
742	M	1	5	1

Table S6. Length and count data for influenza A putative ORFs accompanying Figure S4 (M2).

Position	Kozak	Frame	Length	Count
1	W	1	97	24218
42	M	3	9	23922
42	S	3	9	287
42	M	3	13	1
54	W	3	5	23265
54	W	3	9	1
54	M	3	5	1
147	W	3	6	17366
147	W	3	10	3323
147	W	3	20	1436
147	W	3	37	41
147	W	3	4	16
147	M	3	6	10
147	M	3	10	8
147	W	3	2	2
147	W	3	3	1
214	W	1	26	24197
214	M	1	26	1
245	M	2	12	7950
245	M	2	2	56
245	W	2	12	3
245	M	2	9	2
245	M	2	10	2
245	S	2	12	1
254	W	2	9	23960
254	M	2	9	27
254	W	2	11	19
254	W	2	6	17
254	W	2	7	4
263	S	2	6	17459
263	M	2	6	1102
263	S	2	8	18
263	S	2	3	17
263	S	2	4	3

Table S7. Length and count data for influenza A putative ORFs accompanying Figure S5 (M42).

Position	Kozak	Frame	Length	Count
1	W	1	99	18211
1	W	1	9	124
1	W	1	7	7
1	W	1	3	5
1	W	1	2	1
1	W	1	98	1
48	M	3	9	18095
48	S	3	9	245
60	W	3	5	17564
153	W	3	6	10785
153	W	3	10	4064
153	W	3	20	1420
153	W	3	37	17
153	M	3	6	9
153	M	3	10	8
153	W	3	4	5
153	W	3	2	1
220	W	1	26	18333
220	M	1	26	1
220	W	1	25	1
251	M	2	12	8743
251	M	2	2	67
251	W	2	12	3
251	M	2	9	2
251	M	2	10	2
251	S	2	12	1
260	W	2	9	18090
260	M	2	9	27
260	W	2	6	18
260	W	2	11	11
260	W	2	7	4
269	S	2	6	10853
269	M	2	6	1106
269	S	2	3	18
269	S	2	8	10
269	S	2	4	3

Table S8. Length and count data for influenza A putative ORFs accompanying Figure S6 (NS1).

Position	Kozak	Frame	Length	Count
1	W	1	230	13338
1	W	1	219	6419
1	W	1	225	2080
1	W	1	217	1288
16	M	1	214	5097
16	M	1	225	134
16	M	1	212	13
16	M	1	220	3
16	W	1	214	2
45	M	3	13	2198
45	M	3	7	3
45	M	3	18	3
45	M	3	10	1
45	M	3	17	1
45	M	3	3	1
50	M	2	26	8026
50	M	2	10	1332
50	M	2	16	917
50	M	2	6	61
50	M	2	47	27
50	W	2	26	4
50	M	2	5	2
50	M	2	35	2
50	M	2	52	1
50	W	2	16	1
50	M	2	8	1
70	M	1	207	2292
70	M	1	194	2
74	S	2	18	6305
74	S	2	2	27
74	S	2	44	8
74	S	2	8	7
74	M	2	18	6
74	S	2	13	1
79	M	1	204	2384
79	S	1	204	545
79	S	1	191	158
79	S	1	199	45
79	S	1	193	7

79	M	1	193	5
79	M	1	191	2
86	M	2	14	22031
86	M	2	4	983
86	M	2	35	27
86	M	2	40	8
86	W	2	14	6
86	M	2	7	4
86	M	2	23	4
86	M	2	9	1
98	W	2	10	2300
98	W	2	3	4
175	S	1	172	2225
175	M	1	172	4
175	S	1	159	2
176	W	2	6	3670
176	W	2	5	745
176	W	2	10	6
176	W	2	20	3
176	W	2	4	2
176	W	2	26	1
198	S	3	11	5087
198	S	3	25	52
198	S	3	8	14
198	M	3	11	7
198	S	3	5	7
198	M	3	7	3
198	S	3	7	2
221	W	2	5	9155
221	W	2	19	1793
221	W	2	11	1521
221	M	2	5	287
221	W	2	3	192
221	W	2	16	146
221	M	2	19	59
221	M	2	11	6
221	M	2	16	2
221	W	2	7	2
221	M	2	4	1
221	W	2	6	1
221	W	2	37	1
221	W	2	21	1
221	W	2	14	1

235	M	1	152	10786
235	M	1	141	6346
235	M	1	147	2007
235	M	1	139	1120
235	S	1	152	57
235	S	1	141	38
235	S	1	139	33
235	W	1	152	9
235	W	1	141	1
241	S	1	150	5106
241	S	1	137	14
241	M	1	150	4
277	M	1	138	15402
277	M	1	127	6157
277	M	1	125	1287
277	S	1	127	15
277	S	1	138	5
277	S	1	125	1
292	M	1	133	9107
292	M	1	122	6405
292	M	1	120	1272
292	S	1	133	3
292	S	1	122	1
303	S	3	37	1734
303	S	3	6	370
303	S	3	24	218
303	S	3	51	61
303	S	3	57	51
303	S	3	16	39
303	S	3	29	12
303	S	3	8	3
303	S	3	31	2
303	M	3	37	1
303	S	3	14	1
303	S	3	46	1
303	S	3	23	1
310	W	1	127	15393
310	W	1	116	6396
310	W	1	114	1284
310	M	1	127	4
310	M	1	116	3
310	M	1	114	3
316	W	1	125	15157

316	W	1	114	6357
316	W	1	112	440
316	M	1	125	19
316	M	1	114	3
346	M	1	115	2195
346	W	1	115	6
346	M	1	102	1
355	S	1	112	15295
355	S	1	99	1222
355	M	1	101	1045
355	S	1	101	103
355	M	1	112	34
355	M	1	99	11
370	S	1	107	14818
370	S	1	96	6171
370	S	1	94	764
370	M	1	107	334
370	M	1	94	3
370	M	1	96	2
385	M	1	102	3749
385	M	1	91	12
385	M	1	89	8
385	W	1	102	1
458	S	2	4	1879
458	S	2	3	9
458	M	2	4	7
458	S	2	6	4
496	W	1	65	2263
496	W	1	52	1
558	M	3	2	16506
558	M	3	3	6392
558	S	3	2	7
558	S	3	3	7
664	S	1	9	12803
664	M	1	9	2349

Table S9. Length and count data for influenza A putative ORFs accompanying Figure S7 (NS2).

Position	Kozak	Frame	Length	Count
1	W	1	121	21536
16	M	1	116	4850
16	W	1	116	1
40	W	1	108	12887
40	M	1	108	39
46	M	1	106	21504
55	M	1	103	21452
55	S	1	103	1
86	M	2	2	15425
86	M	2	3	5915
86	S	2	2	7
86	S	2	3	5
91	M	1	91	15428
91	S	1	91	5934
148	M	1	72	20849
148	W	1	72	570
154	S	1	70	19118
154	M	1	70	27
185	M	2	14	5816
185	S	2	7	415
185	M	2	7	114
185	S	2	14	44
185	M	2	17	10
185	W	2	14	1
185	M	2	11	1
192	S	3	9	12036
192	M	3	9	2244
192	S	3	16	1058
192	S	3	7	41
192	M	3	16	32
192	M	3	3	21
192	S	3	2	4
192	S	3	21	1
192	M	3	17	1
231	S	3	3	8593
231	M	3	13	1561
231	M	3	4	274
231	M	3	6	132
231	S	3	8	102

231	M	3	11	15
231	M	3	3	13
231	M	3	30	9
231	S	3	13	7
231	S	3	30	2
231	M	3	8	2
231	S	3	15	2
231	M	3	32	1
231	S	3	4	1
231	M	3	17	1
246	M	3	8	871
246	W	3	8	203
246	W	3	12	11
246	W	3	6	10
246	M	3	6	10
246	M	3	25	6
246	W	3	10	4
246	M	3	27	1
247	M	1	39	4838
298	W	1	22	19232

Table S10. Length and count data for influenza A putative ORFs accompanying Figure S8 (NS3).

Position	Kozak	Frame	Length	Count
1	W	1	194	512
50	M	2	6	263
50	M	2	10	184
50	M	2	26	10
50	M	2	16	1
86	M	2	14	509
86	M	2	4	1
176	W	2	5	204
176	W	2	6	9
221	W	2	5	496
221	W	2	16	13
221	M	2	5	1
221	W	2	3	1
235	M	1	116	508
235	W	1	116	2
241	S	1	114	486
241	M	1	114	12
277	M	1	102	511
277	S	1	102	1
292	M	1	97	207
310	W	1	91	512
316	W	1	89	512
355	S	1	76	308
370	S	1	71	493
370	M	1	71	18
429	M	3	2	511
535	S	1	16	511
574	S	1	3	508
574	M	1	3	1

Table S11. Length and count data for influenza A putative ORFs accompanying Figure S9 (H1).

Position	Kozak	Frame	Length	Count
1	W	1	566	11238
47	W	2	6	10317
47	M	2	6	73
47	W	2	21	42
47	W	2	17	9
47	M	2	21	1
47	W	2	4	1
47	W	2	25	1
60	M	3	27	9114
60	M	3	10	533
60	M	3	7	488
60	M	3	13	233
60	M	3	34	87
60	M	3	6	7
60	W	3	7	3
60	W	3	27	2
60	W	3	10	1
60	M	3	12	1
60	W	3	13	1
60	W	3	30	1
60	M	3	17	1
74	M	2	8	9699
74	M	2	16	916
74	M	2	12	573
74	M	2	11	6
74	M	2	25	5
74	M	2	18	3
74	W	2	8	2
74	S	2	8	2
74	S	2	12	1
164	S	2	6	1238
164	S	2	9	4
164	M	2	6	4
174	M	3	12	9383
174	M	3	31	94
174	M	3	14	60
174	M	3	26	14
174	M	3	57	2
174	M	3	55	1

185	M	2	2	372
185	M	2	12	205
185	M	2	34	5
185	M	2	7	3
228	S	3	8	609
228	M	3	8	504
228	M	3	13	216
228	M	3	39	23
228	M	3	26	20
228	M	3	24	16
228	M	3	27	10
228	S	3	37	7
228	M	3	14	4
228	M	3	21	3
228	S	3	4	3
228	S	3	34	2
228	M	3	6	2
228	S	3	6	1
228	M	3	52	1
228	S	3	39	1
276	M	3	8	9830
276	M	3	23	404
276	M	3	18	375
276	S	3	10	155
276	S	3	23	98
276	M	3	21	73
276	S	3	8	23
276	M	3	11	14
276	M	3	5	13
276	M	3	10	10
276	S	3	5	5
276	M	3	36	5
276	S	3	21	3
276	S	3	18	3
276	S	3	36	1
276	M	3	12	1
311	S	2	14	10718
311	S	2	18	59
311	S	2	9	56
311	M	2	14	3
311	M	2	9	1
311	S	2	8	1
318	M	3	9	833

318	M	3	4	381
318	M	3	7	276
318	M	3	22	7
318	M	3	6	3
318	M	3	25	2
318	W	3	9	2
318	W	3	4	1
344	M	2	3	11158
344	M	2	7	59
344	S	2	3	5
344	W	2	3	1
417	M	3	4	10663
417	M	3	16	168
417	M	3	2	36
417	S	3	16	32
417	M	3	30	14
417	S	3	61	13
417	M	3	61	12
417	M	3	44	9
417	S	3	46	7
417	M	3	46	5
417	M	3	7	4
417	M	3	37	4
417	M	3	20	4
417	S	3	4	3
417	M	3	40	2
417	M	3	120	2
417	M	3	15	1
417	M	3	11	1
417	S	3	2	1
417	M	3	233	1
417	M	3	84	1
417	M	3	23	1
428	M	2	6	10081
428	M	2	25	685
428	M	2	22	122
428	M	2	2	30
428	M	2	8	4
428	W	2	6	4
428	M	2	26	3
428	M	2	4	2
428	M	2	12	1
428	S	2	6	1

456	M	3	17	9109
456	M	3	31	710
456	M	3	3	250
456	W	3	24	234
456	M	3	33	96
456	W	3	17	94
456	M	3	7	78
456	W	3	6	59
456	M	3	27	44
456	M	3	10	25
456	M	3	48	25
456	W	3	33	24
456	W	3	51	17
456	M	3	26	14
456	M	3	4	11
456	W	3	7	8
456	M	3	2	6
456	M	3	24	5
456	W	3	49	3
456	W	3	60	3
456	W	3	3	3
456	M	3	20	2
456	M	3	107	2
456	W	3	31	1
456	M	3	6	1
456	M	3	13	1
456	W	3	10	1
456	M	3	71	1
456	M	3	220	1
464	W	2	10	10014
464	W	2	13	42
464	W	2	3	22
464	W	2	14	12
464	M	2	10	5
464	M	2	13	5
464	W	2	28	1
464	W	2	76	1
498	S	3	3	9222
498	S	3	17	814
498	S	3	10	256
498	S	3	34	192
498	S	3	19	104
498	S	3	13	59

498	S	3	12	13
498	S	3	44	5
498	S	3	21	4
498	M	3	3	4
498	S	3	37	3
498	S	3	6	3
498	S	3	57	3
498	S	3	35	3
498	S	3	16	2
498	S	3	93	2
498	M	3	17	1
498	M	3	206	1
551	W	2	47	8978
551	W	2	7	76
551	W	2	34	19
551	W	2	49	19
551	W	2	28	15
551	W	2	8	7
551	W	2	43	2
551	M	2	47	1
551	W	2	35	1
551	W	2	18	1
551	W	2	5	1
579	S	3	8	7808
579	S	3	10	1377
579	S	3	7	282
579	S	3	99	259
579	S	3	42	82
579	S	3	9	50
579	S	3	30	27
579	S	3	34	17
579	S	3	87	10
579	M	3	10	9
579	S	3	150	8
579	S	3	17	7
579	S	3	18	7
579	M	3	9	6
579	S	3	66	5
579	M	3	7	3
579	S	3	97	3
579	S	3	81	2
579	S	3	31	2
579	S	3	85	2

579	M	3	8	2
579	S	3	92	2
579	S	3	179	1
579	S	3	13	1
579	S	3	93	1
579	S	3	36	1
579	S	3	62	1
579	S	3	28	1
632	M	2	20	10024
632	M	2	7	215
632	M	2	22	19
632	M	2	26	13
632	M	2	8	11
632	M	2	16	4
632	M	2	33	2
632	M	2	14	2
632	W	2	20	1
632	S	2	20	1
632	M	2	10	1
638	W	2	18	9434
638	M	2	5	183
638	W	2	5	174
638	M	2	18	66
638	W	2	20	9
638	W	2	24	9
638	M	2	24	4
638	W	2	6	2
638	W	2	14	2
638	W	2	31	2
638	W	2	8	1
638	W	2	12	1
644	W	2	16	10395
644	W	2	3	419
644	W	2	18	19
644	W	2	22	13
644	W	2	4	11
644	W	2	12	4
644	W	2	29	2
644	W	2	10	2
644	M	2	16	1
644	W	2	2	1
644	W	2	6	1
730	M	1	323	10795

730	S	1	323	136
804	S	3	7	672
804	S	3	10	219
804	S	3	37	17
804	S	3	36	9
804	S	3	53	5
804	M	3	7	4
804	S	3	56	3
804	S	3	24	2
804	S	3	22	2
809	M	2	33	9128
809	M	2	4	1598
809	M	2	5	45
809	M	2	13	32
809	M	2	3	12
809	M	2	18	3
809	W	2	4	3
809	W	2	33	3
809	M	2	15	1
809	M	2	30	1
809	M	2	54	1
809	M	2	19	1
809	M	2	2	1
809	M	2	6	1
820	S	1	293	9281
820	M	1	293	484
820	W	1	293	3
830	M	2	26	9183
830	M	2	6	11
830	M	2	11	3
830	W	2	26	2
830	S	2	26	2
830	M	2	47	1
830	M	2	12	1
857	W	2	17	963
857	W	2	43	43
857	W	2	38	11
857	W	2	11	1
857	M	2	17	1
899	M	2	3	842
899	M	2	29	36
899	M	2	24	14
932	M	2	18	613

932	M	2	6	183
932	M	2	13	119
932	M	2	4	8
957	M	3	24	9576
957	M	3	35	520
957	M	3	8	385
957	M	3	5	257
957	M	3	2	133
957	W	3	35	16
957	M	3	53	12
957	W	3	24	9
957	M	3	7	5
957	W	3	5	1
957	W	3	8	1
957	W	3	53	1
968	M	2	6	1202
968	M	2	12	4
991	S	1	236	1976
991	M	1	236	5
1010	M	2	25	8847
1010	M	2	24	430
1010	M	2	45	102
1010	M	2	17	7
1010	W	2	25	2
1010	M	2	59	1
1010	M	2	10	1
1071	S	3	15	1051
1071	S	3	32	128
1071	S	3	5	56
1071	S	3	33	18
1071	S	3	23	16
1071	M	3	15	4
1071	S	3	13	1
1071	S	3	36	1
1081	S	1	206	9342
1081	M	1	206	1885
1088	M	2	19	9725
1088	M	2	33	987
1088	M	2	29	444
1088	M	2	26	22
1088	M	2	36	7
1088	M	2	40	3
1088	M	2	8	1

1092	M	3	8	10470
1092	M	3	25	195
1092	M	3	16	28
1092	M	3	6	6
1092	S	3	8	5
1092	M	3	26	3
1092	M	3	29	1
1097	S	2	30	634
1097	S	2	26	431
1097	S	2	16	68
1097	S	2	23	19
1097	S	2	33	7
1097	S	2	5	1
1097	S	2	37	1
1115	M	2	10	9710
1115	M	2	24	843
1115	M	2	20	439
1115	S	2	24	155
1115	M	2	17	24
1115	S	2	20	14
1115	S	2	10	11
1115	M	2	27	7
1115	W	2	24	2
1115	S	2	31	2
1115	S	2	17	1
1115	M	2	31	1
1133	M	2	4	9721
1133	M	2	18	802
1133	M	2	14	417
1133	M	2	11	23
1133	M	2	25	3
1133	W	2	4	1
1133	M	2	21	1
1160	M	2	9	10454
1160	M	2	5	417
1160	M	2	2	25
1160	M	2	4	22
1160	M	2	16	8
1160	M	2	12	1
1207	M	1	164	11232
1286	W	2	30	10071
1286	W	2	4	545
1286	W	2	23	285

1286	W	2	14	145
1286	W	2	17	88
1286	W	2	37	22
1286	W	2	28	4
1286	M	2	30	1
1286	W	2	41	1
1289	S	2	29	10097
1289	S	2	3	549
1289	S	2	22	296
1289	S	2	13	144
1289	S	2	16	88
1289	S	2	36	22
1289	S	2	27	4
1289	M	2	29	2
1289	S	2	40	1
1301	W	2	25	680
1301	W	2	18	261
1301	W	2	9	42
1301	W	2	12	39
1301	W	2	32	8
1301	W	2	36	1
1316	M	2	20	10510
1316	M	2	4	167
1316	M	2	13	153
1316	M	2	7	142
1316	M	2	27	24
1316	M	2	18	4
1316	M	2	31	1
1343	M	2	11	10800
1343	M	2	4	232
1343	M	2	18	24
1343	M	2	9	12
1343	S	2	11	6
1343	M	2	22	1
1364	W	2	4	990
1364	M	2	4	249
1364	W	2	2	12
1364	M	2	11	5
1364	W	2	11	4
1364	W	2	15	1
1388	W	2	3	10497
1388	W	2	7	670
1388	W	2	33	1

1388	M	2	3	1
1418	M	2	39	10133
1418	M	2	23	582
1418	M	2	4	219
1418	W	2	39	26
1418	M	2	20	19
1418	M	2	34	10
1418	M	2	31	4
1418	M	2	44	3
1418	W	2	23	2
1418	M	2	37	2
1418	M	2	36	1
1436	S	2	33	1717
1436	S	2	17	686
1436	S	2	14	9
1436	S	2	28	9
1436	S	2	25	3
1436	S	2	38	3
1436	S	2	31	1
1461	W	3	2	9171
1461	W	3	14	39
1461	M	3	2	1
1461	W	3	11	1
1466	M	2	23	895
1466	M	2	7	473
1466	M	2	18	7
1466	M	2	15	3
1466	M	2	28	3
1466	M	2	4	3
1466	M	2	21	1
1469	M	2	6	913
1469	M	2	22	31
1469	M	2	3	3
1473	W	3	10	1016
1473	W	3	5	10
1473	W	3	22	7
1473	W	3	13	6
1473	W	3	3	3
1473	W	3	65	2
1473	W	3	7	1
1473	W	3	19	1
1477	M	1	74	11206
1477	W	1	74	5

1477	S	1	74	1
1493	S	2	14	10731
1493	M	2	14	24
1493	S	2	9	11
1493	S	2	19	6
1493	S	2	6	4
1493	S	2	12	2
1493	S	2	11	1
1502	W	2	11	10912
1502	W	2	6	12
1502	W	2	16	5
1502	M	2	11	4
1502	W	2	3	4
1502	W	2	9	2
1502	W	2	8	1
1553	M	2	2	10643
1553	M	2	4	18
1553	M	2	26	7
1553	M	2	13	1
1573	M	1	42	928
1573	W	1	42	10
1573	S	1	42	2
1660	W	1	13	11236
1660	M	1	13	1

Table S12. Length and count data for influenza A putative ORFs accompanying Figure S10 (H2).

Position	Kozak	Frame	Length	Count
1	W	1	562	468
54	M	3	13	180
54	M	3	30	169
54	M	3	7	97
54	M	3	6	11
54	M	3	26	4
54	M	3	10	3
54	M	3	47	2
68	M	2	18	224
68	M	2	12	91
113	M	2	3	167
128	W	2	16	315
158	S	2	6	367
168	M	3	9	231
168	M	3	26	30
179	M	2	26	82
179	M	2	7	46
179	M	2	12	1
222	M	3	8	212
222	S	3	8	20
270	M	3	21	225
270	S	3	21	218
270	M	3	23	5
270	S	3	18	4
270	S	3	23	4
270	S	3	11	4
270	S	3	12	1
270	M	3	20	1
270	M	3	11	1
283	S	1	468	208
305	M	2	14	220
305	S	2	14	129
305	M	2	21	1
312	W	3	7	61
312	W	3	6	1
332	W	2	5	451
332	M	2	5	5
332	W	2	12	1
338	M	2	3	463

338	M	2	10	1
338	W	2	3	1
404	M	2	29	23
404	S	2	29	18
408	M	3	38	132
408	M	3	60	100
408	M	3	17	93
408	M	3	22	17
408	M	3	4	8
408	M	3	42	4
408	M	3	16	4
408	M	3	43	2
408	S	3	60	2
455	M	2	12	30
455	M	2	35	27
455	S	2	12	1
481	S	1	402	463
481	M	1	402	4
556	W	1	377	445
587	W	2	23	223
587	W	2	18	198
587	W	2	12	6
587	W	2	31	5
590	M	2	17	188
590	M	2	22	48
590	M	2	11	14
590	M	2	30	5
590	W	2	17	5
590	W	2	5	3
590	W	2	22	3
590	M	2	5	1
593	M	2	21	156
593	M	2	10	17
593	M	2	16	11
596	M	2	15	23
620	M	2	7	194
620	M	2	12	155
620	M	2	20	1
632	W	2	8	234
632	W	2	3	220
632	W	2	16	5
701	M	2	19	195
701	M	2	13	134

701	M	2	2	56
701	M	2	27	1
718	S	1	323	468
729	M	3	16	31
729	M	3	12	14
743	W	2	5	33
745	M	1	314	143
747	S	3	6	176
747	M	3	6	64
747	M	3	10	27
747	S	3	10	5
747	M	3	25	4
752	M	2	2	205
752	M	2	10	28
797	S	2	13	362
797	S	2	12	4
797	S	2	4	1
797	S	2	30	1
835	M	1	284	458
835	W	1	284	1
873	W	3	23	320
873	W	3	15	110
873	W	3	32	4
873	M	3	23	3
873	W	3	46	1
920	M	2	4	104
920	M	2	6	1
945	W	3	22	50
945	W	3	8	45
945	W	3	2	3
956	M	2	12	45
956	M	2	6	12
956	M	2	27	2
956	M	2	8	1
998	M	2	13	377
998	M	2	17	1
1059	M	3	5	242
1059	S	3	5	220
1069	S	1	206	389
1069	M	1	206	79
1076	M	2	33	230
1076	M	2	29	227
1076	M	2	36	4

1076	M	2	28	3
1085	S	2	30	228
1085	S	2	26	224
1085	S	2	33	4
1085	S	2	25	2
1103	M	2	20	227
1103	M	2	24	215
1103	M	2	27	4
1103	M	2	19	3
1121	M	2	18	234
1121	M	2	14	213
1121	M	2	21	4
1121	M	2	13	3
1157	M	2	6	208
1157	M	2	2	148
1157	M	2	9	4
1157	W	2	6	2
1195	M	1	164	461
1270	S	1	139	463
1277	S	2	13	199
1277	S	2	3	49
1277	S	2	15	5
1289	W	2	9	393
1289	W	2	11	69
1293	M	3	3	126
1293	M	3	13	90
1293	M	3	17	5
1293	M	3	5	4
1304	M	2	4	394
1304	M	2	6	71
1324	M	1	121	468
1331	M	2	11	248
1331	M	2	22	208
1331	M	2	2	3
1352	W	2	4	227
1352	W	2	15	208
1352	M	2	4	27
1361	W	2	12	210
1376	W	2	7	253
1390	M	1	99	466
1406	M	2	4	374
1406	M	2	20	12
1424	S	2	14	238

1424	S	2	17	11
1424	S	2	33	1
1428	W	3	2	387
1428	W	3	6	9
1454	M	2	4	417
1454	M	2	23	12
1457	M	2	3	442
1457	M	2	22	12
1457	M	2	6	11
1461	W	3	3	282
1461	W	3	10	168
1461	W	3	5	2
1465	W	1	74	444
1465	M	1	74	23
1481	S	2	14	379
1481	S	2	22	1
1490	W	2	11	452
1490	W	2	19	1
1505	M	2	6	467
1505	M	2	14	1
1532	M	2	5	206
1532	M	2	7	14
1561	S	1	42	468
1589	W	2	3	178
1589	W	2	12	50
1624	M	1	21	459
1624	S	1	21	7
1627	S	1	20	96
1644	M	3	4	80
1648	W	1	13	468

Table S13. Length and count data for influenza A putative ORFs accompanying Figure S11 (H3).

Position	Kozak	Frame	Length	Count
1	W	1	566	8025
33	W	3	11	6137
33	W	3	9	107
33	W	3	36	19
33	W	3	5	14
33	M	3	11	7
33	W	3	10	3
33	W	3	13	3
65	M	2	19	5912
65	W	2	19	143
65	M	2	20	46
65	M	2	9	34
65	S	2	9	13
65	S	2	19	9
65	M	2	4	6
65	S	2	20	5
65	M	2	15	2
65	M	2	30	2
65	M	2	14	1
65	M	2	13	1
76	S	1	541	718
76	M	1	541	14
101	M	2	7	7840
101	M	2	8	77
101	M	2	2	4
101	M	2	3	2
101	M	2	18	2
113	M	2	3	619
113	S	2	3	30
113	M	2	4	10
113	S	2	4	5
140	W	2	35	3769
140	W	2	5	3468
140	W	2	17	506
140	W	2	31	82
140	W	2	3	24
140	W	2	11	17
140	W	2	26	3
140	W	2	19	2

140	M	2	17	1
161	W	2	28	6573
161	W	2	4	629
161	W	2	10	520
161	W	2	24	173
161	W	2	29	16
161	W	2	73	16
161	W	2	19	5
161	W	2	12	4
161	W	2	21	3
201	M	3	8	6636
201	M	3	5	737
201	M	3	25	384
201	M	3	2	39
201	M	3	30	4
201	W	3	8	2
201	M	3	16	1
201	M	3	4	1
201	M	3	13	1
227	M	2	6	7712
227	M	2	2	230
227	M	2	7	34
227	M	2	51	12
227	W	2	6	1
251	W	2	43	7075
251	W	2	3	467
251	W	2	20	123
251	W	2	72	11
251	W	2	2	8
251	W	2	40	6
251	W	2	83	5
251	W	2	34	2
251	W	2	50	2
251	W	2	62	1
278	S	2	34	5857
278	M	2	34	1430
278	M	2	11	581
278	S	2	63	11
278	S	2	31	5
278	M	2	74	3
278	M	2	5	3
278	S	2	11	2
278	S	2	74	2

278	M	2	41	2
278	M	2	53	1
278	S	2	25	1
278	M	2	25	1
278	M	2	31	1
290	M	2	30	935
290	M	2	7	585
290	W	2	30	47
290	M	2	49	3
290	S	2	30	3
290	M	2	37	2
290	M	2	27	1
290	S	2	7	1
297	S	3	5	6282
297	S	3	17	412
297	S	3	39	97
297	S	3	11	43
297	S	3	8	10
297	M	3	17	9
297	S	3	25	4
297	S	3	61	1
297	M	3	5	1
297	S	3	12	1
297	S	3	7	1
308	W	2	24	525
308	W	2	21	1
308	W	2	43	1
323	M	2	19	588
323	M	2	26	3
323	M	2	38	3
323	M	2	16	1
347	W	2	11	6893
347	W	2	30	12
347	W	2	40	11
347	W	2	8	7
347	W	2	18	4
347	W	2	51	2
347	W	2	2	1
350	M	2	10	7417
350	M	2	29	12
350	M	2	39	11
350	M	2	7	7
350	M	2	50	5

350	W	2	10	2
350	M	2	17	2
362	M	2	6	7843
362	M	2	25	11
362	M	2	35	11
362	M	2	3	7
362	M	2	46	5
362	M	2	13	3
362	W	2	6	1
413	M	2	18	2996
413	M	2	29	2194
413	W	2	29	585
413	W	2	8	19
413	M	2	8	6
413	M	2	22	2
413	W	2	18	1
426	M	3	18	573
426	M	3	29	68
426	M	3	10	44
426	M	3	13	37
426	M	3	23	5
426	M	3	17	1
426	M	3	45	1
446	S	2	18	4320
446	S	2	7	66
446	S	2	11	2
446	M	2	18	1
446	S	2	21	1
458	M	2	14	1955
458	M	2	3	27
458	M	2	7	1
482	M	2	6	458
482	S	2	6	2
482	M	2	9	1
550	M	1	383	7972
550	W	1	383	2
560	M	2	42	6105
560	M	2	31	624
560	S	2	42	364
560	M	2	32	350
560	M	2	52	292
560	M	2	30	34
560	M	2	28	12

560	M	2	37	10
560	M	2	3	2
560	M	2	59	2
560	S	2	32	2
560	S	2	37	2
560	M	2	26	2
560	S	2	28	1
560	S	2	31	1
581	W	2	24	605
581	W	2	35	62
614	M	2	24	1683
614	M	2	19	8
614	M	2	12	7
614	M	2	14	5
614	M	2	34	2
614	M	2	10	1
632	W	2	18	6386
632	W	2	7	443
632	W	2	8	350
632	W	2	28	298
632	W	2	6	27
632	W	2	4	12
632	W	2	13	12
632	W	2	27	5
632	W	2	2	2
632	W	2	35	2
632	M	2	4	1
632	W	2	16	1
722	M	2	5	589
770	M	2	2	505
770	M	2	3	202
791	S	2	3	1516
791	M	2	3	1
826	M	1	291	1515
826	W	1	291	1
850	M	1	283	7985
850	S	1	283	3
850	W	1	283	1
860	W	2	26	7835
860	W	2	11	34
860	W	2	43	20
860	W	2	3	16
860	W	2	2	13

860	W	2	8	3
860	W	2	17	2
860	M	2	26	1
876	W	3	3	5730
876	W	3	14	612
876	W	3	8	60
876	M	3	3	52
876	W	3	10	2
876	W	3	13	1
888	W	3	10	6816
888	W	3	4	101
888	W	3	29	98
888	W	3	9	24
888	M	3	4	9
888	M	3	10	8
888	M	3	9	7
888	W	3	6	4
888	W	3	18	1
902	M	2	12	7829
902	M	2	29	22
902	S	2	12	7
902	M	2	3	2
917	W	2	7	7839
917	M	2	7	24
917	W	2	24	23
953	M	2	12	4173
953	M	2	7	38
953	M	2	37	4
953	M	2	33	2
960	M	3	20	577
960	M	3	2	568
960	M	3	5	534
960	M	3	24	29
960	M	3	63	24
960	M	3	70	10
960	M	3	54	4
960	M	3	17	3
960	W	3	2	2
960	W	3	5	1
971	M	2	6	7718
971	M	2	31	4
971	W	2	6	2
971	M	2	27	1

1006	M	1	231	8022
1006	W	1	231	1
1013	M	2	17	4530
1013	M	2	13	2673
1013	M	2	59	4
1013	M	2	24	2
1013	M	2	3	1
1013	W	2	13	1
1013	S	2	13	1
1070	S	2	40	3937
1070	S	2	5	1665
1070	S	2	6	1132
1070	S	2	20	1017
1070	S	2	33	18
1070	S	2	23	10
1070	S	2	15	8
1070	S	2	36	4
1070	S	2	43	3
1070	S	2	26	3
1070	S	2	27	1
1070	S	2	32	1
1074	M	3	25	530
1074	M	3	16	454
1074	M	3	32	15
1074	M	3	23	10
1074	M	3	11	7
1074	M	3	13	1
1074	M	3	47	1
1084	S	1	205	6142
1084	M	1	205	1883
1087	S	1	204	478
1091	M	2	33	3237
1091	M	2	13	1024
1091	M	2	26	18
1091	M	2	19	10
1091	M	2	29	3
1091	M	2	8	2
1091	M	2	36	2
1091	M	2	25	1
1100	S	2	30	717
1100	S	2	16	688
1100	S	2	26	6
1100	S	2	10	6

1100	S	2	33	5
1100	S	2	23	1
1181	M	2	3	5912
1181	M	2	6	8
1181	W	2	3	6
1214	W	2	13	923
1214	W	2	29	395
1214	W	2	24	27
1214	W	2	20	3
1214	W	2	11	1
1214	W	2	50	1
1214	W	2	6	1
1283	M	2	6	6639
1283	M	2	27	7
1308	M	3	17	849
1308	M	3	20	168
1308	M	3	3	13
1308	M	3	5	3
1308	M	3	2	1
1319	M	2	15	1518
1319	M	2	7	247
1319	M	2	5	106
1319	M	2	20	8
1319	M	2	4	8
1319	M	2	12	2
1378	M	1	107	8007
1378	S	1	107	10
1378	W	1	107	7
1421	M	2	20	7901
1421	M	2	38	72
1421	M	2	4	19
1421	M	2	9	13
1421	M	2	24	3
1421	M	2	10	2
1421	S	2	20	1
1421	M	2	3	1
1421	M	2	49	1
1432	S	1	89	7978
1432	M	1	89	2
1439	S	2	14	7435
1439	S	2	32	74
1439	S	2	3	14
1439	S	2	18	3

1439	S	2	43	1
1439	M	2	14	1
1439	S	2	4	1
1439	M	2	4	1
1472	M	2	3	7167
1472	M	2	21	39
1472	M	2	7	3
1472	W	2	21	1
1472	M	2	32	1
1476	W	3	10	698
1476	W	3	12	6
1476	W	3	5	6
1496	S	2	13	7108
1496	M	2	13	43
1496	S	2	24	2
1505	W	2	10	7453
1505	M	2	10	6
1505	W	2	21	3
1511	M	2	8	4866
1511	S	2	8	5
1511	M	2	19	3
1514	M	2	7	6307
1514	W	2	7	4
1526	M	2	3	5525
1526	M	2	14	1
1596	M	3	28	7438
1596	S	3	28	2
1596	M	3	26	1
1617	M	3	21	7927
1617	W	3	21	24
1617	M	3	19	2
1654	M	1	15	7978

Table S14. Length and count data for influenza A putative ORFs accompanying Figure S12 (H4).

Position	Kozak	Frame	Length	Count
1	W	1	564	909
75	M	3	8	423
75	M	3	17	242
75	M	3	18	215
75	M	3	19	3
75	W	3	17	2
75	M	3	43	1
79	M	1	538	151
89	M	2	8	886
89	M	2	2	12
89	M	2	7	4
101	M	2	4	864
101	M	2	3	5
109	S	1	528	902
128	W	2	3	823
128	W	2	25	33
128	W	2	12	23
128	W	2	11	5
128	W	2	5	2
189	M	3	8	61
189	M	3	13	47
189	M	3	2	11
189	M	3	49	1
189	M	3	5	1
215	M	2	19	602
215	M	2	11	44
215	M	2	7	5
215	M	2	6	3
215	M	2	28	1
215	W	2	19	1
239	M	2	11	812
239	M	2	3	54
239	M	2	20	10
239	S	2	11	1
239	W	2	11	1
275	M	2	8	879
275	W	2	8	13
275	M	2	28	1
285	S	3	17	620

285	M	3	17	41
285	S	3	62	32
285	S	3	35	15
285	M	3	39	5
285	M	3	11	4
285	S	3	31	2
285	S	3	30	2
285	S	3	20	1
285	S	3	25	1
285	S	3	8	1
290	M	2	3	790
311	W	2	16	85
311	W	2	20	21
311	W	2	2	19
316	S	1	459	45
338	W	2	11	744
338	W	2	7	132
338	W	2	43	21
338	W	2	29	1
338	M	2	11	1
338	W	2	50	1
380	S	2	29	698
380	S	2	15	136
380	S	2	6	10
380	S	2	36	7
380	M	2	29	1
414	M	3	13	439
414	M	3	19	370
414	M	3	8	29
414	M	3	4	12
414	M	3	22	11
414	M	3	31	3
414	M	3	17	2
414	M	3	10	1
414	M	3	18	1
434	S	2	11	881
434	S	2	18	10
450	M	3	7	329
450	M	3	19	3
450	M	3	5	2
450	M	3	6	1
470	W	2	6	858
470	M	2	6	4

470	W	2	9	4
509	M	2	9	693
509	W	2	9	1
515	M	2	7	900
551	S	2	31	627
551	S	2	52	25
551	S	2	42	23
551	S	2	18	1
551	S	2	25	1
560	M	2	28	527
560	M	2	39	110
560	M	2	49	27
560	M	2	15	1
560	M	2	22	1
576	M	3	8	618
576	M	3	16	154
576	M	3	13	5
576	M	3	18	1
576	M	3	40	1
702	S	3	7	508
702	S	3	58	141
702	S	3	37	56
702	S	3	9	40
702	S	3	40	5
702	S	3	24	1
870	W	3	3	43
870	W	3	2	32
870	W	3	15	31
870	W	3	8	11
870	M	3	15	7
870	M	3	2	2
870	W	3	48	2
870	W	3	33	2
882	W	3	11	261
882	W	3	3	155
882	W	3	44	42
882	W	3	4	17
882	W	3	29	5
882	W	3	26	1
965	M	2	6	891
965	M	2	12	2
1000	M	1	231	908
1000	S	1	231	1

1064	S	2	5	897
1064	S	2	40	1
1068	M	3	5	52
1068	M	3	25	9
1068	M	3	23	2
1085	M	2	33	838
1085	M	2	19	46
1085	M	2	29	10
1085	M	2	80	7
1085	M	2	61	2
1094	S	2	30	816
1094	S	2	16	27
1094	S	2	26	13
1094	S	2	77	7
1094	S	2	58	1
1112	M	2	24	820
1112	M	2	10	41
1112	M	2	20	8
1112	M	2	71	7
1112	M	2	52	2
1175	M	2	3	834
1175	M	2	50	52
1175	M	2	31	2
1208	W	2	13	699
1208	W	2	39	54
1208	W	2	42	31
1208	W	2	20	24
1208	W	2	48	23
1208	W	2	40	2
1277	M	2	19	547
1277	M	2	17	157
1277	M	2	16	137
1277	M	2	25	53
1277	M	2	6	9
1302	M	3	5	314
1302	M	3	3	122
1302	M	3	20	5
1302	M	3	28	3
1302	M	3	22	1
1313	M	2	7	554
1313	M	2	5	158
1313	M	2	4	142
1313	M	2	13	53

1372	M	1	107	906
1372	S	1	107	3
1415	M	2	23	771
1415	M	2	49	123
1415	M	2	47	8
1415	M	2	38	1
1415	M	2	70	1
1415	M	2	74	1
1433	S	2	17	739
1433	S	2	43	105
1433	S	2	41	8
1433	S	2	32	1
1433	S	2	64	1
1433	S	2	68	1
1437	W	3	2	602
1437	W	3	8	177
1490	S	2	24	675
1490	S	2	22	8
1490	S	2	13	3
1490	S	2	49	2
1490	S	2	45	1
1490	S	2	19	1
1499	W	2	21	868
1499	W	2	19	10
1499	W	2	10	5
1499	W	2	46	2
1499	W	2	16	1
1499	M	2	21	1
1499	W	2	42	1
1505	M	2	19	831
1505	M	2	17	7
1505	M	2	8	5
1505	S	2	19	5
1505	M	2	44	2
1505	M	2	14	1
1505	M	2	40	1
1508	M	2	18	43
1508	M	2	16	3
1520	M	2	14	185
1520	M	2	12	8
1520	M	2	3	1
1520	M	2	35	1
1520	M	2	39	1

1520	S	2	14	1

Table S15. Length and count data for influenza A putative ORFs accompanying Figure S13 (H5).

Position	Kozak	Frame	Length	Count
1	W	1	568	1501
71	M	2	11	1319
71	M	2	36	171
71	W	2	11	2
71	M	2	4	1
71	S	2	11	1
71	M	2	12	1
106	S	1	533	1497
106	M	1	533	1
131	W	2	16	762
131	W	2	5	53
131	W	2	19	13
182	M	2	2	1254
182	M	2	5	78
225	M	3	8	1434
225	S	3	8	46
225	M	3	28	12
225	M	3	4	1
244	W	1	487	1488
263	W	2	7	1474
263	M	2	7	3
263	W	2	29	1
273	S	3	12	1394
273	S	3	21	29
273	S	3	23	25
273	S	3	11	12
273	S	3	8	8
273	M	3	12	8
273	S	3	20	5
273	M	3	23	1
308	W	2	14	1428
308	M	2	14	50
308	W	2	8	2
308	W	2	18	1
308	M	2	5	1
308	W	2	12	1
335	W	2	5	367
335	M	2	5	1
341	M	2	3	1491

341	M	2	7	1
341	W	2	3	1
422	M	2	6	1154
422	M	2	4	340
422	M	2	35	2
422	M	2	34	1
422	S	2	6	1
450	M	3	10	946
450	M	3	31	477
450	M	3	20	36
450	M	3	5	8
450	M	3	17	7
450	M	3	30	6
450	W	3	10	4
450	M	3	48	2
450	M	3	47	2
450	W	3	3	2
450	M	3	28	1
450	M	3	27	1
485	M	2	14	1423
485	M	2	13	33
485	S	2	14	1
485	W	2	14	1
492	S	3	17	1364
492	S	3	6	40
492	S	3	33	12
492	S	3	3	9
492	S	3	34	7
492	S	3	16	7
492	M	3	17	3
492	S	3	7	1
492	S	3	14	1
492	S	3	13	1
512	M	2	5	616
512	M	2	4	20
571	M	1	378	131
593	W	2	23	1366
593	W	2	6	60
593	W	2	31	2
593	W	2	25	2
593	W	2	12	2
593	W	2	13	1
593	M	2	23	1

596	M	2	22	1404
596	M	2	5	79
596	M	2	11	3
596	M	2	30	2
596	M	2	24	2
596	M	2	6	1
596	M	2	12	1
596	S	2	22	1
724	S	1	327	1266
724	M	1	327	5
755	W	2	29	783
755	W	2	3	668
755	W	2	21	15
755	W	2	2	11
755	W	2	12	8
755	W	2	20	2
755	W	2	28	2
755	W	2	26	1
758	M	2	28	733
758	M	2	2	583
758	M	2	20	15
758	M	2	11	8
758	M	2	19	2
758	M	2	27	1
779	S	2	21	1467
779	S	2	13	15
779	S	2	4	10
779	S	2	20	3
779	S	2	12	2
779	S	2	18	1
779	M	2	21	1
803	M	2	13	1473
803	M	2	5	15
803	M	2	12	3
803	M	2	4	2
803	M	2	10	1
841	M	1	288	1500
860	S	2	14	776
860	S	2	11	701
860	M	2	11	1
860	S	2	35	1
860	S	2	8	1
892	M	1	271	783

913	M	1	264	1496
951	M	3	18	1308
951	M	3	55	120
951	M	3	8	18
951	M	3	16	11
951	M	3	2	5
951	M	3	23	2
951	M	3	7	1
951	M	3	5	1
951	M	3	57	1
951	M	3	21	1
1077	S	3	13	1415
1077	S	3	15	34
1077	M	3	13	27
1077	S	3	37	20
1077	S	3	5	2
1077	S	3	14	1
1087	S	1	206	1495
1087	M	1	206	5
1094	M	2	26	1475
1094	M	2	40	4
1103	S	2	23	1445
1103	S	2	37	7
1121	M	2	17	783
1121	S	2	17	19
1121	W	2	17	1
1121	M	2	31	1
1175	M	2	13	1460
1175	M	2	9	22
1175	M	2	6	15
1175	M	2	2	2
1213	M	1	164	1501
1288	S	1	139	1494
1288	M	1	139	3
1307	W	2	18	1422
1307	W	2	62	41
1307	W	2	11	22
1307	W	2	9	12
1307	W	2	25	2
1307	W	2	12	1
1322	M	2	13	1395
1322	M	2	57	41
1322	M	2	6	22

1322	M	2	4	10
1322	M	2	20	2
1322	M	2	7	1
1342	M	1	121	1498
1342	W	1	121	1
1349	M	2	4	1447
1349	M	2	48	44
1349	M	2	11	2
1370	W	2	41	1390
1370	W	2	4	84
1370	W	2	38	9
1370	W	2	57	9
1370	W	2	22	1
1370	W	2	15	1
1370	M	2	4	1
1379	W	2	38	1390
1379	W	2	35	9
1379	W	2	54	9
1379	M	2	38	1
1379	W	2	19	1
1379	W	2	12	1
1424	M	2	23	1472
1424	M	2	20	11
1424	M	2	39	9
1424	M	2	4	1
1442	S	2	17	123
1467	W	3	2	78
1475	M	2	6	1436
1475	S	2	6	19
1475	M	2	3	11
1475	M	2	22	9
1479	W	3	10	1329
1479	W	3	5	2
1479	W	3	2	1
1479	W	3	71	1
1483	M	1	74	1489
1508	W	2	11	1218
1508	W	2	9	3
1508	W	2	8	3
1508	W	2	16	2
1508	W	2	6	1
1508	W	2	7	1
1579	M	1	42	126

1579	S	1	42	1
1642	S	1	21	1183
1642	M	1	21	301
1645	S	1	20	148
1645	M	1	20	4
1666	W	1	13	1499

Table S16. Length and count data for influenza A putative ORFs accompanying Figure S14 (H6).

Position	Kozak	Frame	Length	Count
1	W	1	566	1085
25	M	1	558	93
57	M	3	27	165
57	M	3	17	159
57	M	3	6	109
57	M	3	69	2
57	M	3	7	1
71	M	2	8	410
71	M	2	16	279
71	M	2	25	210
71	M	2	12	146
71	M	2	37	31
71	M	2	18	6
71	M	2	11	1
71	W	2	16	1
116	M	2	10	217
116	M	2	22	31
116	M	2	3	30
116	M	2	29	5
205	M	1	498	127
210	M	3	18	366
210	M	3	13	89
210	M	3	3	6
210	M	3	29	6
210	M	3	60	1
246	W	3	6	668
246	W	3	17	56
246	M	3	6	22
299	W	2	11	474
299	W	2	14	1
308	S	2	8	1049
308	S	2	11	3
308	S	2	7	3
308	M	2	8	2
315	M	3	20	123
315	M	3	7	28
315	M	3	25	1
335	W	2	2	407
335	W	2	5	282

335	M	2	5	1
394	M	1	435	1083
394	W	1	435	1
411	M	3	17	392
411	M	3	16	223
411	M	3	7	105
411	M	3	6	82
411	M	3	14	53
411	M	3	62	11
411	M	3	32	7
411	M	3	40	7
411	S	3	6	2
411	M	3	23	1
411	M	3	34	1
458	W	2	13	74
458	M	2	13	1
461	S	2	12	114
461	M	2	12	1
461	W	2	12	1
464	S	2	11	224
464	M	2	11	1
492	M	3	13	729
492	M	3	35	61
492	S	3	5	43
492	S	3	13	41
492	M	3	3	4
492	M	3	5	2
492	M	3	20	1
492	S	3	7	1
599	W	2	22	130
605	W	2	20	530
605	M	2	20	6
614	M	2	17	340
614	M	2	31	2
623	M	2	14	1010
623	M	2	8	2
623	M	2	28	2
641	M	2	8	351
649	S	1	350	1040
664	M	1	345	1082
664	S	1	345	1
710	M	2	13	707
710	M	2	19	37

710	M	2	6	2
710	M	2	2	1
770	W	2	7	563
770	W	2	46	12
782	M	2	3	768
782	M	2	42	12
782	S	2	3	1
806	M	2	34	517
806	M	2	33	303
806	M	2	30	138
806	M	2	20	49
806	M	2	5	10
806	M	2	7	5
806	M	2	18	4
806	M	2	4	2
806	M	2	31	1
806	M	2	11	1
806	M	2	43	1
878	M	2	10	569
878	M	2	9	356
878	M	2	6	134
878	M	2	7	1
878	M	2	19	1
885	W	3	11	415
885	W	3	4	336
885	W	3	10	23
885	W	3	31	7
885	W	3	46	6
885	M	3	11	6
885	W	3	3	5
885	W	3	17	3
885	M	3	4	1
932	M	2	18	85
932	M	2	6	6
932	M	2	13	1
957	M	3	7	797
957	M	3	5	117
957	M	3	22	60
957	M	3	24	16
957	M	3	16	4
968	M	2	6	136
1010	M	2	24	492
1010	M	2	4	411

1010	M	2	13	142
1010	M	2	25	18
1010	M	2	17	8
1071	S	3	13	623
1071	S	3	5	359
1071	S	3	32	4
1071	S	3	37	3
1071	S	3	64	1
1081	M	1	206	936
1081	S	1	206	142
1088	M	2	26	929
1088	M	2	40	15
1088	M	2	37	5
1092	M	3	6	285
1092	M	3	25	4
1092	M	3	30	4
1092	M	3	57	1
1097	S	2	23	913
1097	S	2	37	6
1097	S	2	34	1
1109	M	2	19	1058
1109	M	2	33	12
1109	M	2	30	5
1133	M	2	11	1061
1133	M	2	25	12
1133	M	2	22	5
1169	M	2	13	92
1169	M	2	10	16
1169	M	2	6	8
1169	M	2	9	2
1207	M	1	164	1080
1207	W	1	164	1
1207	S	1	164	1
1235	M	2	12	424
1235	M	2	9	111
1235	M	2	5	87
1235	M	2	47	1
1282	S	1	139	1073
1282	M	1	139	8
1289	S	2	22	648
1289	S	2	3	301
1289	S	2	29	54
1289	S	2	13	36

1289	S	2	82	2
1289	S	2	40	1
1301	W	2	18	702
1301	W	2	25	162
1301	W	2	9	51
1301	W	2	78	2
1301	M	2	36	1
1316	M	2	13	537
1316	M	2	20	281
1316	M	2	4	32
1316	M	2	7	2
1316	M	2	73	2
1343	M	2	11	164
1343	M	2	4	47
1343	S	2	11	6
1343	M	2	64	2
1360	M	1	113	251
1364	W	2	4	1066
1364	W	2	15	4
1364	W	2	57	2
1364	M	2	4	2
1367	M	2	3	919
1367	M	2	14	4
1388	W	2	7	763
1388	W	2	3	148
1388	W	2	14	104
1388	W	2	49	40
1388	W	2	30	22
1388	M	2	3	1
1418	M	2	4	827
1418	M	2	44	137
1418	M	2	39	52
1418	M	2	20	37
1418	M	2	2	3
1424	W	2	2	787
1424	W	2	18	33
1424	W	2	37	22
1436	S	2	14	369
1436	S	2	33	156
1436	S	2	38	140
1436	S	2	17	5
1440	W	3	2	689
1461	W	3	3	91

1466	M	2	23	40
1466	M	2	28	10
1466	M	2	7	4
1466	M	2	4	1
1469	M	2	22	516
1469	M	2	3	397
1469	M	2	27	140
1469	M	2	6	5
1473	W	3	10	546
1473	W	3	5	133
1473	W	3	17	76
1473	W	3	2	4
1473	W	3	4	1
1473	W	3	7	1
1477	M	1	74	422
1493	S	2	14	924
1493	S	2	19	144
1493	M	2	14	1
1502	W	2	11	607
1502	W	2	16	134
1502	M	2	11	1
1523	M	2	4	243
1523	M	2	9	1
1561	M	1	46	130
1642	S	1	19	270
1648	S	1	17	1024
1648	M	1	17	3
1660	W	1	13	1085
1678	W	1	7	1083
1678	M	1	7	1

Table S17. Length and count data for influenza A putative ORFs accompanying Figure S15 (H7).

Position	Kozak	Frame	Length	Count
1	W	1	560	750
34	W	1	549	284
50	W	2	17	387
50	W	2	12	2
63	M	3	21	298
63	M	3	35	24
63	M	3	11	20
63	M	3	69	4
63	M	3	17	1
77	M	2	8	732
77	M	2	2	9
77	M	2	11	4
77	M	2	3	3
77	M	2	18	1
89	M	2	4	351
89	M	2	7	4
89	S	2	4	2
89	M	2	14	1
137	W	2	42	284
137	W	2	29	164
137	W	2	8	110
137	W	2	5	17
137	W	2	23	15
137	W	2	19	11
137	W	2	32	6
137	W	2	10	3
137	W	2	31	2
137	W	2	21	1
164	W	2	33	37
164	W	2	20	6
164	W	2	23	5
164	W	2	39	1
177	M	3	31	278
177	M	3	25	27
177	M	3	8	9
177	M	3	10	1
177	M	3	29	1
213	W	3	13	404
213	M	3	19	275

213	M	3	13	38
213	W	3	19	8
213	M	3	17	1
249	W	3	7	273
249	W	3	9	4
249	W	3	5	1
249	M	3	7	1
272	W	2	3	82
272	W	2	4	4
305	M	2	9	36
305	M	2	14	13
305	M	2	36	2
308	M	2	8	409
308	W	2	13	284
308	M	2	13	43
308	M	2	35	2
335	W	2	4	692
335	M	2	4	23
335	W	2	26	5
391	S	1	430	416
391	M	1	430	329
422	M	2	17	694
422	M	2	2	45
422	M	2	9	6
422	M	2	7	1
464	W	2	3	745
472	M	1	403	750
477	S	3	7	206
477	S	3	15	179
477	S	3	90	11
477	S	3	6	10
477	S	3	18	8
477	S	3	5	6
477	S	3	54	4
477	S	3	39	2
477	S	3	57	2
477	S	3	8	2
477	S	3	80	1
477	S	3	9	1
500	M	2	6	649
500	M	2	19	90
500	M	2	5	1
500	S	2	6	1

517	W	1	388	646
564	S	3	28	311
564	S	3	25	49
564	S	3	51	22
564	S	3	17	6
564	S	3	13	4
564	S	3	20	2
564	S	3	10	2
564	S	3	36	1
593	W	2	12	80
611	M	2	6	529
611	M	2	7	132
698	M	2	11	409
698	M	2	19	324
698	M	2	13	1
733	W	1	316	346
733	M	1	316	7
746	W	2	12	416
746	W	2	3	310
746	W	2	17	2
746	M	2	12	1
770	M	2	4	739
770	M	2	17	3
770	M	2	9	2
820	M	1	287	396
836	M	2	18	309
836	M	2	3	26
836	M	2	19	4
836	M	2	50	2
848	W	2	14	397
848	W	2	15	6
878	S	2	4	118
939	M	3	5	365
939	M	3	44	275
939	M	3	62	40
939	M	3	20	24
939	M	3	36	19
939	M	3	2	6
939	M	3	25	5
939	M	3	82	1
939	W	3	20	1
939	M	3	23	1
950	M	2	12	406

950	M	2	8	231
950	M	2	6	92
950	M	2	4	1
970	W	1	237	92
985	M	1	232	750
992	M	2	14	386
992	M	2	18	60
992	M	2	25	9
1052	S	2	5	384
1052	S	2	33	326
1052	S	2	36	19
1052	S	2	40	1
1056	M	3	5	653
1056	M	3	23	47
1056	M	3	32	25
1056	M	3	43	1
1073	M	2	29	328
1073	M	2	26	316
1073	M	2	33	1
1077	M	3	16	290
1077	M	3	25	43
1077	M	3	18	9
1077	M	3	36	1
1077	M	3	2	1
1082	S	2	26	416
1082	S	2	23	324
1082	M	2	23	1
1082	S	2	30	1
1100	M	2	20	419
1100	M	2	17	301
1100	M	2	24	1
1159	W	1	174	46
1210	M	1	157	70
1220	M	2	5	422
1220	M	2	12	297
1229	W	2	2	114
1229	W	2	9	4
1253	M	2	9	319
1253	M	2	2	2
1253	M	2	29	1
1253	M	2	21	1
1279	W	1	134	573
1279	M	1	134	6

1290	S	3	5	175
1290	S	3	3	72
1301	M	2	5	372
1301	M	2	4	195
1301	M	2	13	60
1301	M	2	7	1
1301	M	2	12	1
1301	W	2	13	1
1321	S	1	120	744
1321	M	1	120	1
1360	M	1	107	668
1360	S	1	107	79
1373	W	2	7	57
1373	W	2	3	36
1373	W	2	33	2
1403	M	2	49	370
1403	M	2	23	342
1403	M	2	42	5
1403	M	2	56	1
1403	W	2	23	1
1403	M	2	47	1
1403	M	2	41	1
1415	S	2	45	390
1415	S	2	19	333
1415	S	2	38	10
1415	S	2	43	2
1415	S	2	52	1
1415	S	2	37	1
1425	W	3	2	319
1451	M	2	33	393
1451	M	2	7	338
1451	M	2	26	4
1451	M	2	31	2
1451	M	2	40	1
1451	M	2	25	1
1454	M	2	32	202
1454	M	2	6	4
1454	M	2	24	1
1454	M	2	39	1
1462	M	1	73	750
1487	W	2	21	404
1487	W	2	16	323
1487	W	2	14	7

1487	W	2	19	2
1487	M	2	21	1
1487	W	2	28	1
1487	W	2	13	1
1516	M	1	55	418
1578	M	3	2	314
1624	S	1	19	743
1624	M	1	19	2
1639	W	1	14	39
1660	M	1	7	749

Table S18. Length and count data for influenza A putative ORFs accompanying Figure S16 (H8).

Position	Kozak	Frame	Length	Count
1	W	1	566	92
19	S	1	560	18
19	M	1	560	8
25	M	1	558	92
44	W	2	18	90
44	W	2	22	1
50	W	2	16	21
60	M	3	39	53
60	M	3	41	18
60	M	3	7	11
60	M	3	10	9
60	M	3	50	1
119	M	2	23	72
119	M	2	9	9
119	M	2	12	4
119	M	2	37	2
119	M	2	27	2
119	M	2	10	1
139	S	1	520	92
236	M	2	9	41
316	M	1	461	92
371	W	2	7	73
414	M	3	22	13
414	M	3	29	8
414	M	3	39	1
414	M	3	4	1
419	M	2	26	8
419	M	2	13	1
447	M	3	11	83
447	M	3	18	8
447	M	3	28	1
452	M	2	15	75
452	M	2	2	5
521	W	2	40	66
521	W	2	15	2
530	W	2	37	83
530	W	2	12	8
542	M	2	33	63
542	W	2	33	10

551	M	2	30	78
551	M	2	5	7
557	S	2	28	84
557	S	2	3	7
576	M	3	7	71
576	M	3	16	10
576	M	3	23	8
599	W	2	14	20
629	M	2	4	92
727	S	1	324	83
727	M	1	324	9
782	M	2	3	87
833	S	2	3	85
833	S	2	46	1
851	M	2	40	84
851	M	2	5	1
851	M	2	77	1
885	M	3	9	73
885	M	3	8	14
896	W	2	25	82
896	W	2	62	1
932	M	2	13	90
932	M	2	50	1
949	M	1	250	92
957	M	3	16	77
957	M	3	8	1
1071	S	3	5	47
1081	M	1	206	92
1088	M	2	36	88
1088	M	2	29	1
1088	M	2	33	1
1092	M	3	7	45
1092	M	3	16	21
1092	M	3	6	10
1097	S	2	33	89
1097	S	2	26	1
1097	S	2	30	1
1132	S	1	189	91
1207	M	1	164	92
1235	M	2	9	90
1235	M	2	11	1
1267	M	1	144	88
1267	S	1	144	2

1274	W	2	3	70
1274	W	2	21	8
1274	W	2	18	1
1286	W	2	17	82
1286	W	2	14	1
1316	M	2	7	54
1316	M	2	4	1
1358	W	2	13	8
1364	M	2	11	87
1373	W	2	8	92
1391	W	2	2	86
1418	W	2	2	90
1418	W	2	23	2
1427	W	2	20	85
1436	S	2	17	33
1461	W	3	3	34
1461	W	3	2	15
1469	M	2	6	82
1469	S	2	6	1
1473	W	3	12	36
1477	M	1	74	92
1493	S	2	14	72
1493	S	2	22	8
1493	S	2	19	1
1517	M	2	6	83
1517	M	2	14	8
1517	M	2	11	1
1553	M	2	2	84
1663	M	1	12	92
1687	W	1	4	92

Table S19. Length and count data for influenza A putative ORFs accompanying Figure S17 (H9).

Position	Kozak	Frame	Length	Count
1	W	1	560	1180
19	W	1	554	144
19	M	1	554	10
25	M	1	552	144
34	M	1	549	54
34	S	1	549	4
34	W	1	549	3
50	M	2	17	591
50	M	2	20	470
50	M	2	6	51
50	W	2	17	19
50	W	2	20	9
50	M	2	27	2
50	M	2	21	2
50	W	2	6	1
63	M	3	19	32
63	M	3	54	16
63	M	3	32	10
63	M	3	34	8
63	M	3	26	2
63	M	3	17	1
122	M	2	3	828
122	W	2	3	287
122	M	2	29	17
122	W	2	29	1
137	W	2	24	854
137	W	2	18	64
137	W	2	32	21
137	W	2	67	21
137	W	2	22	10
137	W	2	4	2
167	S	2	14	1014
167	S	2	8	76
167	S	2	22	31
167	S	2	57	21
167	S	2	12	17
167	M	2	14	5
167	S	2	48	3
167	M	2	8	1

167	S	2	19	1
172	M	1	503	1170
177	M	3	16	189
177	M	3	26	39
177	M	3	14	22
177	M	3	22	11
177	M	3	11	2
216	W	3	13	107
216	W	3	3	91
216	W	3	9	55
216	W	3	51	1
216	M	3	3	1
239	M	2	33	700
239	M	2	24	243
239	M	2	9	55
239	M	2	18	34
239	M	2	36	18
239	M	2	17	16
239	M	2	39	1
239	M	2	19	1
279	S	3	11	562
279	S	3	30	213
279	S	3	5	209
279	S	3	33	118
279	S	3	86	27
279	S	3	26	16
279	S	3	84	5
279	S	3	74	2
279	S	3	145	1
279	M	3	11	1
279	S	3	85	1
314	M	2	8	882
314	M	2	11	80
314	M	2	37	3
314	M	2	14	1
319	M	1	454	283
319	W	1	454	1
321	M	3	16	71
321	M	3	19	8
321	M	3	12	7
321	M	3	70	1
335	M	2	4	79
335	M	2	30	3

335	M	2	7	2
422	M	2	18	685
422	M	2	6	7
422	M	2	11	1
422	M	2	8	1
422	W	2	18	1
434	S	2	14	84
434	S	2	2	3
434	M	2	14	1
450	M	3	28	811
450	M	3	29	85
450	M	3	68	65
450	M	3	6	56
450	M	3	45	54
450	M	3	16	38
450	M	3	2	19
450	M	3	27	11
450	M	3	13	8
450	M	3	14	4
450	W	3	28	3
450	M	3	17	2
450	M	3	88	1
450	M	3	50	1
450	W	3	27	1
450	M	3	15	1
450	W	3	15	1
450	M	3	57	1
475	M	1	402	1180
480	S	3	18	683
480	S	3	58	94
480	S	3	19	90
480	S	3	35	65
480	S	3	6	43
480	S	3	17	12
480	S	3	3	7
480	S	3	46	6
480	S	3	4	4
480	S	3	5	4
480	S	3	40	2
480	S	3	47	1
480	S	3	78	1
500	M	2	23	63
500	M	2	45	7

500	M	2	50	3
500	M	2	20	1
518	M	2	39	45
518	M	2	17	42
518	M	2	31	9
518	M	2	36	5
518	M	2	44	3
536	M	2	33	134
536	S	2	11	4
536	M	2	38	2
536	S	2	25	2
536	M	2	30	1
559	W	1	374	902
695	M	2	13	776
695	M	2	2	179
695	W	2	13	6
695	M	2	12	2
695	M	2	6	2
695	M	2	4	1
695	M	2	3	1
703	M	1	326	94
767	M	2	3	1045
767	M	2	21	3
767	W	2	3	2
786	M	3	17	855
786	M	3	22	146
786	M	3	30	60
786	M	3	20	32
786	M	3	12	22
786	S	3	17	6
786	M	3	15	6
786	M	3	11	4
786	M	3	45	2
786	M	3	8	1
786	M	3	21	1
786	S	3	12	1
786	M	3	2	1
786	S	3	15	1
786	M	3	72	1
791	S	2	13	988
791	S	2	5	78
791	S	2	17	7
791	M	2	13	6

791	S	2	7	3
818	S	2	4	515
867	M	3	18	557
867	M	3	3	423
867	M	3	45	97
867	W	3	3	24
867	M	3	48	1
867	M	3	34	1
867	M	3	17	1
867	W	3	18	1
914	M	2	15	258
914	M	2	24	20
914	M	2	13	1
914	M	2	22	1
914	M	2	39	1
926	M	2	11	739
926	M	2	20	214
926	M	2	16	35
926	M	2	9	17
926	M	2	18	8
926	M	2	35	6
926	W	2	11	3
926	M	2	14	1
950	M	2	3	404
950	M	2	12	29
950	M	2	10	2
950	M	2	8	2
950	M	2	27	2
992	M	2	13	927
992	M	2	17	16
992	M	2	5	13
1079	S	2	26	977
1079	S	2	12	58
1079	S	2	10	17
1079	S	2	30	4
1079	S	2	23	3
1079	M	2	26	2
1079	S	2	13	1
1079	S	2	18	1
1097	W	2	20	1066
1097	W	2	6	66
1097	W	2	4	17
1097	W	2	17	5

1097	W	2	24	4
1097	W	2	7	1
1097	M	2	20	1
1097	W	2	12	1
1114	S	1	189	1098
1189	M	1	164	1174
1189	S	1	164	1
1189	W	1	164	1
1202	M	2	16	804
1202	M	2	2	253
1202	M	2	10	37
1202	M	2	14	32
1202	M	2	3	11
1202	M	2	36	7
1202	S	2	16	3
1202	S	2	52	1
1202	S	2	10	1
1217	M	2	11	1077
1217	M	2	9	46
1217	M	2	5	39
1217	M	2	31	7
1217	S	2	11	1
1217	M	2	47	1
1226	W	2	8	60
1226	W	2	6	15
1249	M	1	144	1168
1249	S	1	144	2
1256	W	2	18	97
1256	W	2	34	3
1268	W	2	14	1052
1268	W	2	30	44
1268	W	2	23	15
1268	W	2	4	5
1268	W	2	11	3
1268	W	2	13	1
1268	W	2	3	1
1268	M	2	14	1
1287	M	3	3	912
1287	M	3	11	65
1287	M	3	5	4
1287	M	3	18	2
1298	M	2	4	772
1298	M	2	20	30

1298	M	2	13	15
1298	S	2	4	1
1340	W	2	6	1128
1340	W	2	13	22
1340	M	2	6	1
1346	M	2	4	1113
1346	M	2	11	21
1346	W	2	4	1
1346	S	2	4	1
1349	M	2	3	823
1349	M	2	10	21
1400	W	2	39	963
1400	W	2	44	88
1400	W	2	2	83
1400	W	2	37	10
1400	W	2	30	5
1400	W	2	47	3
1400	M	2	39	3
1400	W	2	31	1
1400	W	2	21	1
1400	W	2	22	1
1405	S	1	92	412
1412	S	2	35	990
1412	S	2	40	87
1412	S	2	27	19
1412	S	2	26	11
1412	S	2	33	6
1412	M	2	35	3
1412	S	2	43	3
1412	M	2	40	1
1412	S	2	18	1
1412	S	2	17	1
1418	S	2	33	137
1418	S	2	38	4
1418	S	2	41	3
1422	M	3	8	364
1422	M	3	6	277
1422	W	3	8	57
1422	W	3	6	36
1422	M	3	2	35
1422	W	3	2	33
1422	W	3	9	1
1422	M	3	9	1

1422	S	3	8	1
1443	W	3	2	116
1443	W	3	15	1
1443	M	3	2	1
1443	W	3	14	1
1443	M	3	14	1
1448	M	2	23	848
1448	M	2	28	87
1448	M	2	15	19
1448	M	2	14	12
1448	M	2	21	8
1448	M	2	31	3
1448	W	2	21	2
1448	M	2	5	1
1448	M	2	6	1
1455	W	3	10	201
1455	W	3	5	174
1455	W	3	33	53
1455	W	3	11	10
1455	W	3	29	1
1455	W	3	13	1
1459	M	1	74	1177
1475	S	2	14	120
1475	S	2	19	6
1484	W	2	11	129
1484	W	2	3	19
1484	W	2	2	7
1484	W	2	16	4
1615	S	1	22	1093
1615	M	1	22	1
1645	M	1	12	1176
1645	W	1	12	1

Table S20. Length and count data for influenza A putative ORFs accompanying Figure S18 (H10).

Position	Kozak	Frame	Length	Count
1	W	1	561	509
44	M	2	18	420
60	M	3	27	402
60	M	3	18	35
60	M	3	49	2
74	M	2	8	479
74	M	2	2	20
74	M	2	18	1
86	M	2	4	502
86	M	2	14	1
113	W	2	5	59
134	W	2	15	410
134	W	2	10	70
134	W	2	5	14
174	M	3	7	48
174	M	3	25	12
174	M	3	11	1
178	W	1	502	509
226	S	1	486	255
226	M	1	486	247
270	S	3	21	380
270	S	3	8	45
270	S	3	5	43
270	M	3	21	6
270	S	3	35	5
270	S	3	7	2
296	M	2	11	65
296	M	2	9	3
296	M	2	16	1
332	W	2	4	502
358	S	1	442	508
358	M	1	442	1
401	M	2	14	454
401	M	2	4	35
401	W	2	14	8
401	M	2	11	2
401	M	2	25	1
401	W	2	4	1
438	M	3	56	254

438	M	3	7	230
438	M	3	50	8
438	M	3	94	5
438	M	3	32	3
438	M	3	20	2
438	M	3	6	1
438	M	3	75	1
438	M	3	13	1
438	M	3	4	1
442	W	1	414	507
449	S	2	9	385
449	S	2	12	39
449	S	2	38	30
449	S	2	2	3
449	S	2	96	1
449	S	2	25	1
467	W	2	3	429
467	W	2	6	39
467	W	2	32	25
467	M	2	3	2
467	W	2	90	1
467	W	2	19	1
480	M	3	6	17
480	S	3	42	9
480	M	3	42	7
480	S	3	36	6
480	M	3	24	2
480	M	3	4	1
565	M	1	373	36
567	S	3	13	18
567	S	3	7	9
605	M	2	12	290
605	M	2	31	115
605	M	2	44	64
605	M	2	24	13
605	M	2	25	4
605	M	2	59	3
605	M	2	11	1
605	M	2	38	1
614	M	2	9	307
614	M	2	28	115
614	M	2	41	56
614	M	2	21	13

614	M	2	22	4
614	M	2	56	3
614	M	2	8	1
614	M	2	35	1
701	M	2	27	403
701	M	2	12	76
701	M	2	6	15
701	M	2	13	2
736	S	1	316	320
736	M	1	316	2
773	S	2	3	391
773	S	2	4	2
854	M	2	34	401
854	M	2	13	12
854	S	2	13	9
854	W	2	34	7
854	S	2	34	3
854	M	2	24	1
870	M	3	88	91
870	M	3	103	82
870	M	3	118	76
870	M	3	8	56
870	W	3	8	52
870	M	3	10	52
870	W	3	26	44
870	M	3	113	12
870	W	3	30	9
870	M	3	95	6
870	W	3	86	6
870	W	3	103	6
870	M	3	30	4
870	W	3	44	3
870	M	3	19	2
870	M	3	100	2
870	W	3	74	1
870	W	3	19	1
942	W	3	94	216
942	W	3	64	96
942	W	3	79	59
942	W	3	2	43
942	W	3	6	15
942	W	3	89	14
942	W	3	97	8

942	W	3	71	6
942	W	3	62	6
942	W	3	20	3
942	W	3	76	2
942	W	3	50	1
973	W	1	237	61
988	M	1	232	509
995	M	2	14	431
995	M	2	4	22
995	M	2	18	8
1009	M	1	225	46
1009	W	1	225	1
1055	S	2	6	474
1055	S	2	40	10
1055	S	2	33	8
1055	S	2	22	1
1055	S	2	5	1
1059	M	3	55	222
1059	M	3	25	96
1059	M	3	40	93
1059	M	3	23	30
1059	M	3	32	19
1059	M	3	50	14
1059	M	3	58	8
1059	M	3	43	4
1059	M	3	37	2
1059	M	3	11	1
1069	S	1	205	504
1069	M	1	205	1
1076	M	2	26	429
1076	M	2	33	53
1076	M	2	29	7
1076	M	2	47	3
1076	M	2	15	1
1085	S	2	23	430
1085	S	2	30	51
1085	S	2	26	7
1085	S	2	44	3
1085	S	2	10	3
1085	S	2	12	1
1103	M	2	17	429
1103	M	2	24	34
1103	M	2	20	7

1103	M	2	38	3
1103	M	2	4	3
1103	M	2	6	1
1324	S	1	120	509
1348	S	1	112	459
1348	M	1	112	48
1363	M	1	107	509
1376	W	2	3	486
1406	M	2	39	441
1406	M	2	23	67
1418	S	2	35	427
1418	S	2	19	9
1428	M	3	2	62
1445	M	2	10	46
1445	M	2	26	1
1449	W	3	14	210
1449	W	3	2	203
1449	W	3	3	15
1449	W	3	19	2
1449	W	3	11	1
1454	M	2	23	121
1454	M	2	7	52
1461	W	3	10	51
1461	W	3	5	3
1461	W	3	35	1
1465	M	1	73	509
1490	W	2	11	497
1571	M	2	26	417
1571	M	2	18	2
1571	M	2	15	2
1571	M	2	17	1
1581	S	3	2	42
1627	S	1	19	508
1663	M	1	7	508

Table S21. Length and count data for influenza A putative ORFs accompanying Figure S19 (H11).

Position	Kozak	Frame	Length	Count
1	W	1	565	382
50	W	2	7	141
50	W	2	4	1
116	M	2	27	225
116	M	2	3	67
116	M	2	9	21
116	M	2	21	3
152	M	2	15	272
152	S	2	15	7
152	M	2	9	3
182	M	2	5	224
225	M	3	8	131
225	S	3	8	5
244	W	1	484	365
251	M	2	2	365
251	S	2	2	5
273	S	3	22	160
273	M	3	22	84
273	S	3	18	49
273	S	3	11	46
273	S	3	5	18
273	M	3	8	13
273	S	3	30	2
273	M	3	21	1
308	M	2	8	252
308	M	2	14	13
315	M	3	8	201
315	W	3	8	5
315	M	3	16	2
315	M	3	4	1
338	M	2	4	342
407	M	2	5	292
407	W	2	5	2
407	M	2	30	1
411	M	3	19	262
411	M	3	94	39
411	M	3	14	21
411	M	3	43	11
411	M	3	7	8

411	M	3	90	3
411	M	3	44	3
411	M	3	10	3
411	M	3	18	1
411	M	3	6	1
411	M	3	4	1
411	M	3	84	1
411	M	3	46	1
428	S	2	23	45
450	W	3	81	39
450	W	3	30	11
450	W	3	6	8
450	W	3	31	3
450	W	3	71	1
450	W	3	33	1
458	S	2	13	103
487	S	1	403	245
487	M	1	403	120
487	W	1	403	16
492	S	3	63	117
492	S	3	17	106
492	S	3	67	49
492	S	3	57	37
492	S	3	16	23
492	S	3	4	15
492	S	3	43	6
492	S	3	37	4
492	S	3	46	2
492	S	3	58	2
492	S	3	69	1
492	S	3	54	1
492	S	3	19	1
560	M	2	2	320
560	M	2	13	34
560	W	2	2	16
560	M	2	27	4
560	S	2	2	1
560	M	2	55	1
568	M	1	376	263
568	W	1	376	1
573	S	3	36	228
573	S	3	30	19
573	S	3	31	4

573	S	3	10	2
573	S	3	19	2
573	M	3	10	2
573	S	3	16	1
573	S	3	42	1
573	S	3	27	1
707	M	2	6	364
707	W	2	6	1
724	M	1	324	296
724	W	1	324	86
779	M	2	12	193
779	M	2	25	142
779	M	2	4	18
779	M	2	15	5
779	M	2	41	2
803	M	2	17	179
803	M	2	4	178
803	M	2	33	2
830	S	2	8	259
867	M	3	12	70
867	M	3	22	23
867	M	3	3	2
867	M	3	79	1
879	W	3	10	237
879	W	3	8	68
879	W	3	3	42
879	W	3	18	23
879	M	3	8	2
879	W	3	11	2
879	W	3	75	1
926	M	2	6	241
926	M	2	13	38
968	W	2	4	373
968	W	2	25	2
1004	M	2	4	193
1004	M	2	13	136
1065	M	3	15	68
1065	M	3	13	26
1065	S	3	5	16
1065	M	3	5	8
1065	S	3	40	7
1065	S	3	15	1
1082	M	2	26	290

1082	M	2	15	80
1086	M	3	8	45
1086	M	3	6	28
1086	M	3	17	14
1086	M	3	33	7
1091	S	2	23	293
1091	S	2	12	22
1109	M	2	17	283
1109	M	2	6	75
1201	W	1	165	285
1201	M	1	165	97
1229	M	2	9	275
1229	M	2	5	17
1229	S	2	9	3
1229	M	2	40	1
1229	M	2	16	1
1274	M	2	25	180
1274	W	2	25	78
1274	M	2	5	16
1274	M	2	19	15
1274	M	2	43	3
1274	M	2	6	1
1280	W	2	23	258
1280	W	2	41	54
1280	W	2	17	16
1280	W	2	14	14
1280	W	2	3	8
1280	W	2	4	6
1280	W	2	30	1
1299	M	3	13	279
1299	M	3	3	6
1299	M	3	20	1
1310	M	2	31	52
1310	M	2	13	40
1310	M	2	4	32
1310	M	2	7	8
1310	M	2	20	1
1337	M	2	4	286
1337	M	2	22	80
1337	M	2	11	15
1358	W	2	15	282
1358	W	2	57	18
1358	W	2	4	16

1358	W	2	11	1
1367	W	2	12	344
1367	W	2	54	18
1367	W	2	8	1
1382	W	2	7	360
1382	W	2	49	18
1382	W	2	3	1
1399	W	1	99	284
1399	M	1	99	96
1412	M	2	39	275
1412	M	2	47	85
1412	M	2	23	1
1412	M	2	49	1
1421	M	2	36	282
1421	M	2	44	48
1421	M	2	20	1
1421	M	2	46	1
1430	S	2	33	283
1430	S	2	41	13
1430	S	2	43	1
1434	W	3	6	84
1434	W	3	8	9
1463	M	2	22	250
1463	M	2	30	44
1463	S	2	22	2
1463	M	2	6	1
1467	W	3	2	200
1467	W	3	5	47
1467	W	3	10	5
1487	S	2	14	283
1487	S	2	22	19
1496	W	2	11	290
1496	W	2	19	76
1496	W	2	21	1
1574	M	2	17	13
1574	M	2	22	8
1574	M	2	21	3
1639	S	1	19	371
1653	M	3	3	9
1653	M	3	8	7
1653	M	3	4	3

Table S22. Length and count data for influenza A putative ORFs accompanying Figure S20 (H12).

Position	Kozak	Frame	Length	Count
1	W	1	564	124
50	W	2	16	120
119	M	2	3	21
119	M	2	27	12
119	M	2	6	3
119	M	2	23	2
152	M	2	12	98
152	M	2	16	16
152	M	2	3	7
174	W	3	11	61
174	W	3	12	8
174	W	3	32	6
174	W	3	22	5
174	W	3	3	2
174	W	3	93	1
174	M	3	22	1
209	W	2	4	98
209	W	2	9	6
209	W	2	7	2
249	W	3	7	15
249	W	3	42	2
249	W	3	68	1
266	M	2	7	97
266	M	2	23	13
266	M	2	8	4
266	M	2	14	1
276	S	3	20	95
276	S	3	36	3
276	S	3	42	1
276	S	3	11	1
276	S	3	8	1
307	S	1	462	121
383	M	2	3	89
397	M	1	432	124
414	M	3	13	71
414	M	3	9	9
414	M	3	22	6
414	S	3	13	1
414	M	3	8	1

419	M	2	23	119
419	M	2	28	2
447	M	3	2	12
452	M	2	12	43
487	M	1	402	121
492	S	3	14	113
492	S	3	13	2
492	M	3	14	2
492	S	3	36	1
533	W	2	26	92
533	W	2	51	30
533	W	2	57	1
573	M	3	9	31
602	W	2	3	75
602	W	2	28	28
602	W	2	34	1
656	W	2	10	79
656	W	2	16	42
724	S	1	323	124
779	M	2	11	105
779	M	2	3	17
803	S	2	3	124
830	M	2	10	98
830	M	2	14	9
830	M	2	19	1
830	M	2	2	1
867	M	3	3	54
867	W	3	8	16
879	W	3	4	38
879	W	3	17	5
879	W	3	26	2
890	W	2	3	70
901	M	1	264	123
951	M	3	88	34
951	M	3	81	33
951	M	3	78	29
951	M	3	2	15
951	M	3	85	4
951	M	3	82	3
951	M	3	54	2
951	M	3	93	2
1004	M	2	17	117
1004	M	2	13	1

1065	M	3	50	34
1065	M	3	40	30
1065	M	3	43	23
1065	S	3	43	6
1065	M	3	47	4
1065	M	3	44	3
1065	S	3	40	2
1065	M	3	55	2
1065	S	3	16	2
1091	S	2	12	79
1091	S	2	23	37
1091	S	2	30	3
1109	M	2	6	83
1109	M	2	17	21
1109	M	2	4	1
1126	S	1	189	22
1168	M	1	175	124
1187	M	2	5	114
1187	M	2	12	1
1201	M	1	164	124
1229	M	2	9	102
1229	M	2	5	3
1261	M	1	144	121
1280	W	2	3	105
1299	M	3	5	63
1299	M	3	18	43
1299	M	3	3	17
1299	M	3	28	1
1310	M	2	13	88
1310	M	2	20	15
1310	M	2	4	3
1310	M	2	3	1
1352	W	2	6	89
1352	W	2	17	12
1358	M	2	4	94
1358	M	2	15	13
1361	M	2	3	33
1361	M	2	14	18
1367	W	2	12	30
1382	W	2	7	99
1412	M	2	47	70
1412	M	2	58	34
1412	M	2	2	18

1412	M	2	20	1
1412	M	2	44	1
1430	S	2	41	74
1430	S	2	52	33
1430	S	2	33	5
1430	S	2	14	2
1430	S	2	38	1
1434	W	3	2	11
1460	M	2	31	18
1460	M	2	23	5
1460	M	2	4	1
1463	S	2	30	7
1463	S	2	22	5
1463	M	2	30	2
1463	S	2	3	1
1467	W	3	21	7
1467	W	3	19	6
1467	W	3	5	3
1467	W	3	15	2
1471	M	1	74	122
1471	W	1	74	2
1487	S	2	33	34
1511	M	2	14	73
1511	M	2	25	34
1511	M	2	11	1
1547	M	2	2	85
1547	M	2	13	34
1630	W	1	21	124

Table S23. Length and count data for influenza A putative ORFs accompanying Figure S21 (H13).

Position	Kozak	Frame	Length	Count
1	W	1	566	38
11	W	2	5	21
11	W	2	8	1
42	W	3	24	8
42	W	3	34	1
42	W	3	20	1
50	W	2	9	5
63	M	3	17	21
63	M	3	27	1
119	S	2	10	17
119	M	2	10	12
119	S	2	4	6
122	M	2	9	15
188	M	2	16	15
188	M	2	7	13
188	M	2	17	9
188	M	2	34	1
231	S	3	21	21
231	S	3	14	1
252	M	3	14	9
252	M	3	7	1
252	W	3	14	1
279	S	3	5	7
314	M	2	8	22
314	M	2	14	16
341	W	2	5	7
347	S	2	18	22
347	S	2	3	14
431	W	2	20	14
431	W	2	24	1
434	S	2	19	14
434	M	2	19	1
434	S	2	23	1
454	M	1	415	3
458	M	2	11	17
495	S	3	6	9
563	M	2	2	37
563	M	2	55	1
574	M	1	375	3

576	M	3	18	17
576	M	3	10	5
605	W	2	26	5
623	W	2	8	24
623	W	2	20	11
623	W	2	35	2
663	S	3	2	10
663	S	3	5	2
663	M	3	2	1
710	S	2	6	38
727	W	1	324	38
748	W	1	317	16
763	M	1	312	22
782	S	2	11	18
782	S	2	4	10
806	S	2	3	37
827	S	2	10	17
827	S	2	26	13
827	S	2	23	8
862	M	1	279	16
870	W	3	38	7
870	W	3	44	1
870	W	3	30	1
882	W	3	26	8
882	W	3	34	7
882	W	3	40	1
941	M	2	9	21
941	M	2	2	17
1007	M	2	17	37
1007	M	2	24	1
1085	M	2	15	23
1085	M	2	29	15
1094	S	2	26	8
1112	M	2	20	8
1112	M	2	6	7
1204	M	1	165	38
1208	M	2	17	23
1208	M	2	13	2
1217	M	2	14	23
1217	M	2	10	8
1217	M	2	3	7
1264	M	1	145	38
1283	W	2	3	30

1286	M	2	2	31
1298	W	2	18	15
1298	W	2	12	1
1313	M	2	13	24
1313	M	2	7	14
1340	M	2	4	37
1357	M	1	114	37
1361	W	2	4	22
1361	W	2	15	16
1364	M	2	3	22
1364	M	2	14	10
1370	W	2	12	16
1385	W	2	7	38
1415	M	2	2	13
1415	M	2	23	9
1415	W	2	2	8
1415	W	2	23	8
1424	W	2	20	16
1433	S	2	17	37
1437	W	3	2	2
1458	W	3	2	22
1463	M	2	7	37
1474	M	1	75	38
1490	S	2	14	30
1499	W	2	11	11
1505	M	2	9	14
1514	M	2	6	38
1550	M	2	2	13
1550	M	2	26	9
1577	M	2	17	23
1577	M	2	18	1
1654	M	1	15	38

Table S24. Length and count data for influenza A putative ORFs accompanying Figure S22 (H14).

Position	Kozak	Frame	Length	Count
1	W	1	568	11
62	M	2	24	11
87	W	3	13	11
101	M	2	11	11
113	S	2	7	5
122	W	2	4	1
194	W	2	7	11
201	M	3	25	7
227	M	2	80	7
251	M	2	26	4
302	M	2	55	7
302	M	2	9	4
328	S	1	459	7
336	W	3	36	4
350	W	2	39	7
431	S	2	12	11
482	S	2	6	6
524	M	2	44	4
533	S	2	41	11
545	W	2	37	7
572	W	2	28	5
617	M	2	13	4
882	M	3	8	7
882	M	3	7	4
941	M	2	6	11
997	W	1	236	11
1012	M	1	231	11
1076	S	2	5	11
1097	M	2	19	11
1106	S	2	16	11
1124	M	2	10	11
1187	M	2	3	11
1220	W	2	42	5
1220	W	2	6	4
1220	W	2	13	2
1325	M	2	7	5
1325	M	2	4	4
1325	M	2	13	2
1367	W	2	6	11

1384	M	1	107	11
1427	M	2	20	11
1449	W	3	8	5
1502	S	2	19	7
1511	W	2	16	11
1532	M	2	9	11
1579	S	1	42	11
1618	W	1	29	11
1623	M	3	16	7

Table S25. Length and count data for influenza A putative ORFs accompanying Figure S23 (H15).

Position	Kozak	Frame	Length	Count
1	W	1	570	10
43	M	1	556	9
77	M	2	2	9
77	M	2	11	1
89	M	2	7	10
137	W	2	10	10
312	W	3	7	1
335	W	2	4	9
391	M	1	440	9
391	S	1	440	1
422	M	2	2	1
472	M	1	413	10
477	S	3	7	10
493	M	1	406	5
564	M	3	20	1
593	W	2	12	9
611	M	2	6	10
698	M	2	28	9
698	M	2	13	1
730	W	1	327	10
770	M	2	4	10
815	W	2	35	9
815	W	2	56	1
839	S	2	27	9
839	S	2	48	1
866	M	2	18	9
878	W	2	14	9
878	W	2	35	1
897	M	3	18	9
897	M	3	8	1
969	M	3	44	1
1015	M	1	232	10
1022	M	2	56	9
1022	M	2	14	1
1082	S	2	36	10
1086	M	3	5	10
1103	M	2	29	10
1107	M	3	4	10
1112	S	2	26	9

1130	M	2	20	10
1250	M	2	9	10
1283	M	2	2	1
1331	M	2	4	9
1331	M	2	5	1
1351	S	1	120	10
1390	M	1	107	10
1403	W	2	3	10
1433	M	2	42	10
1445	S	2	38	10
1455	W	3	2	10
1488	W	3	7	9
1488	W	3	13	1
1492	M	1	73	10
1561	M	1	50	10
1608	S	3	2	10
1636	M	1	25	9
1636	W	1	25	1
1654	S	1	19	10
1669	W	1	14	10

Table S26. Length and count data for influenza A putative ORFs accompanying Figure S24 (H16).

Position	Kozak	Frame	Length	Count
1	W	1	565	31
4	S	1	564	9
4	M	1	564	6
66	M	3	7	30
66	M	3	11	1
122	S	2	4	21
191	M	2	17	21
191	M	2	34	3
234	S	3	21	11
234	S	3	24	11
234	S	3	27	1
282	S	3	5	2
282	S	3	8	1
308	W	2	24	22
308	W	2	11	2
350	S	2	10	31
420	M	3	13	15
420	M	3	14	7
420	M	3	31	5
428	M	2	5	18
428	M	2	25	3
428	M	2	24	2
437	M	2	2	26
437	M	2	22	3
437	M	2	21	2
453	W	3	3	6
453	W	3	20	5
512	M	2	6	15
512	S	2	6	4
512	S	2	5	2
563	M	2	2	17
563	M	2	28	8
563	M	2	3	3
563	M	2	46	3
623	W	2	26	15
623	W	2	8	14
663	S	3	5	20
677	M	2	8	31
707	S	2	6	9

724	W	1	324	31
745	W	1	317	31
766	M	1	310	11
779	S	2	4	29
779	S	2	25	2
803	S	2	17	4
824	S	2	10	4
859	S	1	279	29
859	M	1	279	1
867	W	3	50	13
867	W	3	51	10
867	W	3	14	2
879	M	3	47	10
879	W	3	46	1
879	M	3	46	1
892	M	1	268	15
938	M	2	9	31
951	M	3	22	3
1004	M	2	17	31
1019	W	2	12	11
1065	S	3	5	8
1065	S	3	33	8
1082	M	2	26	16
1086	M	3	17	19
1086	M	3	26	9
1086	M	3	8	3
1091	S	2	23	27
1109	M	2	17	3
1163	W	2	2	31
1201	M	1	165	31
1214	M	2	3	22
1214	M	2	10	1
1261	M	1	145	31
1280	W	2	3	31
1283	M	2	2	31
1295	W	2	18	9
1299	M	3	5	2
1310	M	2	6	18
1310	M	2	13	11
1310	M	2	31	1
1337	M	2	22	13
1337	S	2	4	11
1358	W	2	15	21

1358	W	2	11	1
1361	M	2	14	23
1361	M	2	10	1
1367	W	2	12	30
1367	W	2	8	1
1382	W	2	7	30
1382	W	2	3	1
1412	M	2	39	31
1421	W	2	36	21
1430	S	2	33	27
1455	W	3	2	2
1460	M	2	23	28
1467	W	3	5	16
1467	W	3	10	9
1467	W	3	12	6
1471	M	1	75	31
1487	S	2	14	31
1502	M	2	9	31
1574	M	2	16	21
1574	M	2	18	9
1624	S	1	24	22
1651	M	1	15	31
1659	M	3	2	3
1659	M	3	4	3
1659	M	3	8	2

Table S27. Length and count data for influenza A putative ORFs accompanying Figure S25 (H17).

Position	Kozak	Frame	Length	Count
1	W	1	564	3
63	M	3	9	3
122	M	2	10	3
167	S	2	6	2
177	M	3	26	3
188	M	2	34	2
188	M	2	12	1
216	M	3	13	3
279	S	3	7	3
299	W	2	21	3
305	M	2	19	1
367	M	1	442	3
418	M	1	425	3
440	M	2	30	3
593	M	2	56	3
617	M	2	48	3
623	M	2	46	3
695	M	2	22	3
800	S	2	13	3
841	W	1	284	3
879	W	3	23	3
982	W	1	237	3
1015	M	1	226	3
1075	M	1	206	3
1082	M	2	29	3
1091	S	2	26	3
1163	W	2	2	3
1201	M	1	164	3
1280	W	2	17	3
1283	M	2	16	3
1337	M	2	11	3
1358	W	2	4	3
1361	M	2	3	3
1412	M	2	2	3
1421	W	2	20	3
1430	S	2	17	3
1471	M	1	74	3
1487	S	2	22	3
1504	M	1	63	3

1547	M	2	2	3
1585	M	1	36	3

Table S28. Length and count data for influenza A putative ORFs accompanying Figure S26 (H18).

Position	Kozak	Frame	Length	Count
1	W	1	561	1
51	M	3	7	1
110	W	2	3	1
155	S	2	6	1
219	M	3	8	1
226	M	1	486	1
267	M	3	10	1
302	S	2	11	1
397	M	1	429	1
441	W	3	3	1
449	W	2	11	1
458	M	2	8	1
584	W	2	28	1
608	S	2	20	1
929	M	2	9	1
1056	S	3	5	1
1073	M	2	29	1
1082	S	2	26	1
1118	M	2	14	1
1154	W	2	2	1
1192	M	1	164	1
1240	M	1	148	1
1271	W	2	37	1
1274	S	2	36	1
1286	W	2	32	1
1301	M	2	27	1
1328	M	2	18	1
1349	W	2	11	1
1352	M	2	10	1
1358	W	2	8	1
1403	M	2	47	1
1414	S	1	90	1
1446	W	3	7	1
1458	W	3	3	1
1462	M	1	74	1
1466	W	2	26	1
1496	M	2	16	1
1576	M	1	36	1
1641	M	3	4	1

Table S29. Length and count data for influenza A putative ORFs accompanying Figure S27 (N1).

Position	Kozak	Frame	Length	Count
1	W	1	469	9918
43	W	1	455	8274
43	M	1	455	923
55	S	1	451	7649
55	M	1	451	22
96	S	3	2	9308
96	S	3	14	452
96	M	3	2	46
96	S	3	8	43
96	M	3	8	10
96	S	3	24	9
96	M	3	17	6
96	M	3	14	4
96	S	3	6	2
96	S	3	17	2
96	S	3	26	1
96	S	3	12	1
144	M	3	8	8318
144	M	3	19	457
144	M	3	22	381
144	M	3	28	21
144	M	3	24	21
144	M	3	55	20
144	M	3	10	20
144	M	3	2	13
144	M	3	6	10
144	M	3	39	7
144	M	3	21	7
144	W	3	8	3
144	M	3	46	1
167	W	2	6	8364
167	W	2	11	32
167	W	2	24	14
167	M	2	6	14
167	W	2	27	3
167	M	2	11	1
180	M	3	9	842
180	M	3	15	36
180	M	3	7	24

180	M	3	44	19
180	S	3	9	6
180	M	3	2	4
180	M	3	27	4
180	M	3	16	2
180	M	3	81	1
180	S	3	15	1
180	M	3	41	1
180	M	3	12	1
180	S	3	5	1
180	W	3	2	1
197	W	2	17	7712
197	W	2	14	455
197	W	2	19	72
197	W	2	3	11
197	W	2	13	3
197	W	2	8	2
197	W	2	40	1
197	M	2	19	1
288	M	3	5	8014
288	M	3	7	453
288	M	3	8	310
288	M	3	45	132
288	M	3	14	38
288	M	3	4	23
288	M	3	33	12
288	S	3	45	1
288	S	3	8	1
288	S	3	5	1
311	S	2	2	526
311	M	2	2	1
311	S	2	4	1
338	M	2	4	8249
338	M	2	21	519
338	M	2	3	29
338	M	2	14	4
338	M	2	27	2
338	M	2	2	1
369	M	3	18	7658
369	W	3	18	1911
369	W	3	6	43
369	W	3	22	24
369	W	3	2	17

369	W	3	4	17
369	M	3	4	4
369	M	3	17	4
369	M	3	6	4
369	M	3	26	3
369	W	3	17	1
369	W	3	19	1
384	M	3	13	9453
384	M	3	17	23
384	W	3	13	19
384	M	3	21	3
384	W	3	12	3
384	M	3	12	3
384	S	3	13	2
384	M	3	14	1
384	S	3	12	1
422	W	2	17	9858
422	W	2	8	22
422	W	2	18	1
437	M	2	12	9600
437	M	2	3	22
437	W	2	12	5
437	M	2	13	1
475	W	1	311	9914
475	M	1	311	2
549	W	3	2	935
549	W	3	16	29
549	W	3	60	2
554	M	2	6	9612
554	M	2	3	69
554	M	2	21	11
554	M	2	18	3
557	S	2	5	9770
557	S	2	2	85
557	S	2	20	11
557	S	2	17	3
562	S	1	282	879
562	M	1	282	10
567	S	3	10	879
567	S	3	20	2
567	S	3	9	2
567	S	3	32	2
567	M	3	10	1

567	S	3	24	1
567	S	3	54	1
599	S	2	6	8942
599	S	2	3	562
599	M	2	6	14
599	S	2	11	13
599	S	2	5	1
626	S	2	2	1877
626	M	2	2	41
626	M	2	3	1
690	W	3	17	7925
690	M	3	13	748
690	W	3	4	625
690	W	3	13	470
690	W	3	16	27
690	W	3	23	15
690	W	3	34	11
690	M	3	17	10
690	W	3	42	8
690	M	3	34	4
690	M	3	4	3
690	M	3	16	2
690	W	3	26	1
690	M	3	26	1
690	W	3	14	1
690	M	3	37	1
690	M	3	5	1
696	W	3	15	7893
696	W	3	11	199
696	W	3	2	139
696	W	3	14	30
696	W	3	21	15
696	W	3	40	8
696	W	3	32	2
696	W	3	12	1
696	W	3	35	1
700	W	1	236	517
700	M	1	236	9
704	M	2	6	8891
704	M	2	7	39
724	M	1	228	9309
724	W	1	228	101
724	S	1	228	2

731	M	2	14	8986
731	M	2	19	508
731	M	2	70	129
731	M	2	25	18
731	M	2	2	7
731	M	2	102	7
731	M	2	44	6
731	M	2	15	5
731	M	2	12	3
731	M	2	23	2
731	M	2	20	2
731	W	2	14	1
743	S	2	10	8955
743	S	2	15	514
743	S	2	66	241
743	S	2	21	34
743	M	2	10	24
743	S	2	40	22
743	S	2	19	9
743	S	2	98	7
743	S	2	11	5
743	S	2	16	4
743	S	2	8	3
743	M	2	40	1
743	M	2	66	1
743	M	2	15	1
805	M	1	201	7658
805	S	1	201	13
809	W	2	44	8405
809	W	2	18	1217
809	W	2	76	134
809	W	2	40	31
809	W	2	34	8
809	W	2	17	7
809	W	2	38	1
809	W	2	68	1
827	M	2	38	8249
827	M	2	12	1140
827	M	2	70	132
827	M	2	34	25
827	M	2	28	8
827	M	2	11	6
827	M	2	62	1

834	M	3	5	9075
834	M	3	7	14
834	M	3	32	3
834	M	3	14	2
834	M	3	8	2
834	M	3	16	2
851	W	2	4	711
851	W	2	30	219
851	W	2	26	8
851	W	2	62	4
851	M	2	3	1
865	M	1	181	1205
865	W	1	181	1
867	W	3	5	7782
867	W	3	3	265
867	W	3	39	65
867	M	3	5	29
867	W	3	40	5
867	W	3	11	4
867	W	3	17	3
867	M	3	3	3
867	W	3	35	3
867	M	3	21	2
867	M	3	17	1
867	W	3	21	1
867	M	3	39	1
867	W	3	10	1
873	W	3	3	339
873	W	3	38	309
873	W	3	33	135
873	W	3	21	79
873	W	3	37	25
873	W	3	9	11
873	W	3	19	9
873	W	3	49	2
873	W	3	15	1
873	W	3	32	1
873	W	3	6	1
890	S	2	17	8125
890	M	2	17	892
890	S	2	13	664
890	S	2	49	126
890	M	2	49	36

890	S	2	7	8
890	S	2	11	1
890	S	2	41	1
906	S	3	8	633
906	S	3	27	464
906	S	3	22	145
906	S	3	10	102
906	S	3	26	84
906	S	3	21	49
906	S	3	4	45
906	S	3	6	4
906	S	3	38	3
906	S	3	5	2
906	S	3	16	2
951	W	3	11	7612
951	W	3	6	909
951	W	3	12	775
951	W	3	7	282
951	W	3	23	7
986	W	2	17	9775
986	W	2	9	26
986	W	2	3	5
986	W	2	30	2
986	M	2	17	1
989	S	2	16	1720
989	S	2	8	16
989	M	2	16	8
989	S	2	2	3
989	S	2	29	1
1022	M	2	5	9673
1022	W	2	5	2
1022	M	2	18	2
1022	S	2	5	2
1031	M	2	2	9774
1031	S	2	2	6
1031	M	2	15	2
1058	S	2	6	1033
1058	S	2	20	9
1058	M	2	6	3
1064	S	2	4	9457
1064	S	2	18	200
1064	S	2	34	2
1064	M	2	4	1

1117	M	1	97	9776
1117	S	1	97	48
1133	M	2	11	2044
1133	M	2	16	161
1133	M	2	18	8
1133	M	2	3	6
1133	M	2	10	4
1133	M	2	15	3
1133	M	2	4	1
1137	M	3	6	7933
1137	M	3	4	421
1137	M	3	18	196
1137	M	3	2	140
1137	M	3	5	99
1137	M	3	10	1
1137	M	3	39	1
1165	W	1	81	728
1190	W	2	15	7861
1190	M	2	15	4
1190	W	2	21	1
1243	M	1	55	904
1243	S	1	55	85
1252	W	1	52	1058
1368	S	3	5	918
1368	S	3	11	6

Table S30. Length and count data for influenza A putative ORFs accompanying Figure S28 (N2).

Position	Kozak	Frame	Length	Count
1	W	1	469	9651
60	M	3	20	8616
60	M	3	17	356
60	M	3	33	222
60	M	3	19	43
60	W	3	20	24
60	M	3	22	16
60	M	3	28	6
60	M	3	86	4
60	M	3	27	4
60	M	3	11	3
60	M	3	26	3
60	M	3	49	3
60	S	3	20	1
60	M	3	74	1
60	M	3	64	1
70	W	1	446	9305
70	M	1	446	78
119	M	2	10	7851
119	M	2	17	1050
119	M	2	6	78
119	S	2	10	51
119	M	2	16	33
119	W	2	10	8
119	S	2	17	6
119	S	2	6	4
119	M	2	11	3
119	M	2	18	3
119	W	2	6	2
119	M	2	2	1
119	W	2	17	1
119	M	2	20	1
119	S	2	16	1
123	W	3	12	2701
123	W	3	6	140
123	W	3	28	34
123	W	3	22	8
123	W	3	5	5
123	W	3	51	3

123	M	3	12	2
123	M	3	5	2
123	W	3	7	2
123	W	3	38	1
123	M	3	6	1
123	W	3	43	1
123	W	3	65	1
123	W	3	4	1
151	M	1	419	7840
151	W	1	419	6
151	S	1	419	1
231	M	3	15	6547
231	M	3	7	726
231	M	3	24	475
231	M	3	2	44
231	M	3	5	35
231	M	3	49	29
231	M	3	69	9
231	M	3	29	7
231	M	3	40	3
231	M	3	73	2
231	M	3	64	2
231	M	3	11	2
231	S	3	15	1
231	M	3	47	1
273	M	3	10	598
273	M	3	33	380
273	M	3	36	180
273	M	3	15	160
273	W	3	15	139
273	M	3	35	45
273	W	3	55	40
273	M	3	12	17
273	M	3	59	15
273	W	3	35	9
273	W	3	10	7
273	W	3	51	6
273	M	3	26	6
273	M	3	55	5
273	W	3	3	4
273	M	3	51	4
273	W	3	50	3
273	W	3	36	3

273	W	3	59	2
273	W	3	12	2
273	M	3	21	1
273	W	3	33	1
273	M	3	50	1
362	W	2	19	8642
362	W	2	38	838
362	W	2	13	86
362	M	2	19	8
362	W	2	5	3
362	M	2	38	1
362	W	2	27	1
362	W	2	28	1
369	M	3	3	6776
369	M	3	23	1027
369	M	3	4	738
369	M	3	27	82
369	M	3	19	52
369	W	3	3	25
369	M	3	18	17
369	W	3	4	7
369	M	3	28	2
369	W	3	18	1
384	W	3	22	1156
384	W	3	18	260
384	W	3	23	126
384	W	3	14	106
384	W	3	13	72
384	W	3	12	8
384	W	3	27	6
384	M	3	23	2
384	W	3	51	2
384	M	3	22	1
384	M	3	13	1
384	W	3	15	1
384	W	3	17	1
437	W	2	13	4575
437	M	2	13	1285
437	W	2	2	16
437	W	2	12	2
437	M	2	14	1
437	W	2	14	1
437	W	2	10	1

437	W	2	3	1
449	W	2	9	6351
449	M	2	9	33
449	W	2	10	2
449	W	2	8	1
449	W	2	6	1
478	W	1	310	9647
478	M	1	310	3
482	W	2	15	4029
482	W	2	2	3795
482	W	2	8	975
482	W	2	33	70
482	W	2	51	18
482	W	2	78	4
482	W	2	9	3
482	W	2	50	1
526	M	1	294	730
531	S	3	20	7535
531	S	3	7	1360
531	S	3	21	257
531	S	3	2	157
531	M	3	7	28
531	S	3	4	19
531	S	3	19	16
531	M	3	20	8
531	S	3	26	2
531	S	3	22	2
531	S	3	9	2
531	S	3	8	1
531	M	3	21	1
551	M	2	55	1344
551	M	2	10	138
551	M	2	56	87
551	M	2	28	37
551	M	2	39	31
551	M	2	27	26
551	M	2	26	10
551	M	2	15	5
551	M	2	49	2
551	M	2	31	2
551	M	2	57	2
551	M	2	3	1
554	S	2	9	7341

554	S	2	54	1558
554	S	2	27	456
554	S	2	55	89
554	S	2	38	31
554	S	2	26	27
554	S	2	25	10
554	S	2	14	5
554	S	2	30	2
554	S	2	48	2
554	M	2	9	2
554	S	2	56	2
554	S	2	35	1
554	S	2	2	1
564	S	3	9	9022
564	S	3	10	296
564	S	3	8	20
564	M	3	9	7
564	S	3	15	3
564	S	3	11	2
564	S	3	19	1
572	W	2	3	7321
572	W	2	48	1093
572	W	2	21	419
572	W	2	49	42
572	W	2	32	25
572	W	2	20	22
572	W	2	19	10
572	W	2	8	5
572	W	2	42	2
572	W	2	29	1
590	M	2	15	7106
590	M	2	42	1814
590	M	2	14	219
590	M	2	43	89
590	M	2	26	32
590	M	2	2	11
590	M	2	13	10
590	M	2	8	9
590	M	2	18	2
590	M	2	36	2
590	M	2	44	2
590	M	2	23	1
593	M	2	14	1735

593	M	2	41	100
593	S	2	14	25
593	S	2	41	10
593	M	2	13	8
593	M	2	7	2
593	S	2	42	2
593	M	2	42	1
599	M	2	12	7385
599	M	2	39	1826
599	M	2	11	219
599	M	2	40	91
599	M	2	23	32
599	W	2	12	28
599	M	2	10	10
599	M	2	5	9
599	W	2	39	8
599	W	2	11	2
599	M	2	41	2
599	M	2	15	2
599	M	2	33	2
599	M	2	20	1
620	W	2	32	1322
620	W	2	33	84
620	M	2	32	52
620	W	2	16	32
620	W	2	4	25
620	W	2	5	18
620	W	2	3	10
620	W	2	26	2
620	W	2	8	2
620	W	2	34	2
620	M	2	33	1
623	S	2	4	7311
623	S	2	31	1461
623	S	2	3	221
623	S	2	32	82
623	S	2	15	33
623	M	2	4	3
623	S	2	33	2
623	S	2	25	2
623	S	2	7	2
623	M	2	31	1
623	S	2	12	1

628	M	1	260	1048
628	S	1	260	2
651	M	3	25	8401
651	M	3	2	308
651	M	3	16	248
651	M	3	6	60
651	M	3	33	42
651	M	3	11	35
651	S	3	2	31
651	M	3	17	23
651	M	3	3	19
651	S	3	25	13
651	M	3	31	12
651	M	3	40	7
651	M	3	9	6
651	S	3	6	2
651	S	3	17	1
651	W	3	25	1
651	S	3	33	1
687	M	3	13	8492
687	M	3	4	275
687	M	3	21	157
687	W	3	21	111
687	W	3	13	44
687	M	3	5	27
687	M	3	19	12
687	M	3	28	7
687	W	3	5	4
687	M	3	23	1
687	W	3	23	1
687	W	3	4	1
701	M	2	5	8982
701	M	2	6	339
701	W	2	5	14
701	M	2	4	6
701	M	2	7	3
701	W	2	6	1
721	M	1	229	9629
721	W	1	229	4
728	M	2	60	6480
728	M	2	32	1731
728	M	2	59	568
728	M	2	62	309

728	M	2	26	170
728	M	2	22	123
728	M	2	19	122
728	M	2	44	42
728	M	2	25	35
728	M	2	11	19
728	M	2	14	10
728	M	2	6	8
728	M	2	4	4
728	M	2	106	2
728	M	2	20	1
728	M	2	49	1
728	M	2	12	1
821	M	2	29	6716
821	M	2	28	579
821	M	2	31	343
821	M	2	13	40
821	M	2	75	2
821	M	2	18	1
831	M	3	10	763
831	M	3	21	440
831	M	3	22	256
831	M	3	33	76
831	M	3	14	32
831	M	3	8	28
831	M	3	16	20
831	M	3	5	12
831	W	3	10	1
857	W	2	17	514
857	W	2	16	5
857	W	2	19	3
864	W	3	11	7505
864	W	3	10	1042
864	W	3	22	337
864	W	3	5	285
864	W	3	3	149
864	M	3	11	42
864	M	3	10	12
864	M	3	3	6
864	W	3	20	1
864	M	3	5	1
864	W	3	17	1
919	S	1	163	1612

919	M	1	163	192
947	M	2	33	6840
947	M	2	22	2416
947	M	2	16	12
947	M	2	5	5
947	M	2	6	4
947	M	2	12	4
947	W	2	22	3
947	M	2	28	3
947	M	2	76	2
947	M	2	56	2
947	W	2	33	1
947	M	2	81	1
947	M	2	31	1
951	W	3	17	997
951	W	3	6	187
951	W	3	12	168
951	W	3	9	65
951	W	3	11	23
951	W	3	13	22
951	W	3	15	16
951	W	3	23	6
951	W	3	16	5
986	M	2	20	1421
986	M	2	9	144
986	W	2	20	2
986	M	2	3	1
986	M	2	15	1
986	M	2	68	1
1025	M	2	7	9318
1025	M	2	30	3
1025	M	2	50	3
1025	M	2	2	2
1025	W	2	7	1
1025	M	2	5	1
1040	S	2	2	7651
1040	S	2	25	3
1040	S	2	45	3
1040	M	2	2	1
1064	W	2	17	3789
1064	W	2	37	3091
1064	W	2	42	476
1064	W	2	11	152

1064	W	2	25	12
1064	W	2	24	4
1064	W	2	71	3
1064	W	2	13	2
1064	W	2	41	1
1064	W	2	7	1
1067	S	2	36	3836
1067	S	2	16	3815
1067	S	2	41	1357
1067	S	2	10	172
1067	M	2	41	54
1067	S	2	29	24
1067	S	2	24	20
1067	M	2	16	16
1067	S	2	23	14
1067	M	2	36	8
1067	S	2	40	5
1067	S	2	70	4
1067	M	2	29	4
1067	S	2	12	3
1067	S	2	21	1
1067	S	2	6	1
1073	M	2	34	3816
1073	M	2	14	3780
1073	M	2	39	1513
1073	M	2	8	169
1073	M	2	22	22
1073	M	2	27	17
1073	M	2	21	12
1073	M	2	68	9
1073	M	2	38	8
1073	M	2	10	3
1073	M	2	4	1
1073	M	2	19	1
1076	M	2	38	885
1076	M	2	33	769
1076	M	2	13	109
1076	M	2	26	28
1076	M	2	7	23
1076	M	2	21	21
1076	W	2	33	11
1076	M	2	20	10
1076	M	2	37	7

1076	M	2	67	7
1076	W	2	13	1
1076	M	2	9	1
1076	M	2	18	1
1076	W	2	26	1
1084	M	1	108	9649
1121	M	2	18	6901
1121	M	2	23	2514
1121	M	2	5	29
1121	M	2	11	26
1121	M	2	6	22
1121	M	2	22	14
1121	M	2	52	12
1121	M	2	3	1
1268	W	2	3	9094
1268	W	2	4	12
1268	M	2	3	1
1308	S	3	23	1058
1308	S	3	5	492
1308	S	3	4	6
1308	M	3	23	1
1371	M	3	2	9578
1371	W	3	2	3
1399	W	1	3	9502
1399	M	1	3	137

Table S31. Length and count data for influenza A putative ORFs accompanying Figure S29 (N3).

Position	Kozak	Frame	Length	Count
1	W	1	469	699
107	W	2	3	637
107	M	2	3	27
107	M	2	27	14
107	W	2	26	7
107	W	2	9	5
107	M	2	26	3
107	W	2	8	1
107	W	2	14	1
273	M	3	18	20
273	M	3	10	19
273	M	3	31	4
273	W	3	18	2
311	M	2	11	440
311	M	2	10	178
311	M	2	2	45
311	M	2	4	22
338	M	2	2	58
338	M	2	3	22
362	W	2	19	470
362	W	2	37	116
362	W	2	13	16
362	W	2	28	1
377	M	2	14	482
377	M	2	32	99
377	M	2	8	28
377	M	2	23	4
377	W	2	32	1
380	M	2	31	82
380	M	2	13	30
380	S	2	31	4
380	S	2	7	2
437	S	2	12	685
437	S	2	3	10
525	M	3	10	430
525	M	3	6	197
525	M	3	25	16
525	M	3	7	11
525	M	3	23	7

525	M	3	5	5
525	M	3	24	4
557	M	2	9	236
557	M	2	20	23
567	S	3	11	144
567	S	3	9	53
567	S	3	10	28
571	W	1	279	680
575	W	2	3	616
575	W	2	14	37
579	W	3	6	80
579	W	3	5	19
579	W	3	7	7
583	W	1	275	657
583	M	1	275	2
593	M	2	8	141
599	M	2	6	683
599	M	2	12	3
599	S	2	6	2
602	M	2	5	672
602	M	2	11	3
619	M	1	263	50
623	W	2	4	432
623	M	2	4	225
623	W	2	8	24
623	W	2	16	5
634	M	1	258	687
634	S	1	258	2
634	W	1	258	1
654	S	3	11	524
654	S	3	30	63
654	S	3	16	60
654	S	3	31	34
654	S	3	29	5
654	S	3	25	3
690	W	3	4	531
690	W	3	18	74
690	M	3	19	23
690	W	3	19	17
690	M	3	17	5
690	W	3	13	4
690	M	3	4	1
696	W	3	2	547

696	W	3	16	67
696	W	3	17	25
696	W	3	15	4
696	W	3	11	4
704	M	2	28	76
704	M	2	29	5
704	M	2	5	3
704	M	2	23	1
731	M	2	19	646
731	M	2	14	32
731	M	2	20	18
749	M	2	13	35
790	M	1	206	113
797	M	2	11	475
797	M	2	38	129
797	M	2	8	30
797	M	2	18	18
797	M	2	40	9
797	M	2	6	4
797	M	2	109	2
803	M	2	9	429
803	M	2	36	21
803	M	2	4	4
837	M	3	7	397
837	M	3	13	13
848	M	2	21	450
860	W	2	17	631
860	M	2	17	6
873	W	3	8	39
873	W	3	17	32
873	W	3	28	16
873	W	3	38	12
873	W	3	15	9
910	W	1	166	676
923	M	2	4	187
923	M	2	67	25
923	M	2	3	7
923	M	2	40	5
923	M	2	34	2
923	M	2	53	1
947	M	2	32	418
947	M	2	80	136
947	M	2	110	29

947	M	2	127	27
947	M	2	17	15
947	M	2	45	8
947	M	2	59	4
947	M	2	26	3
947	M	2	62	1
947	M	2	50	1
947	M	2	60	1
951	W	3	12	103
951	W	3	2	52
951	W	3	19	30
951	W	3	11	9
1000	M	1	136	297
1022	M	2	7	400
1022	M	2	55	50
1022	M	2	85	26
1022	M	2	102	4
1022	M	2	25	1
1022	M	2	35	1
1022	M	2	20	1
1028	M	2	5	432
1028	M	2	53	152
1028	M	2	83	29
1028	M	2	100	28
1028	M	2	32	25
1028	M	2	18	8
1028	M	2	23	1
1028	M	2	35	1
1028	M	2	33	1
1070	M	2	4	257
1070	M	2	39	231
1070	M	2	86	46
1070	M	2	18	28
1070	M	2	69	26
1070	M	2	66	12
1070	M	2	21	1
1070	M	2	9	1
1073	M	2	3	295
1073	M	2	38	239
1073	M	2	85	79
1073	M	2	68	29
1073	M	2	17	28
1073	M	2	65	12

1073	M	2	21	1
1073	M	2	18	1
1073	M	2	8	1
1077	M	3	3	389
1077	M	3	8	164
1077	M	3	14	74
1077	M	3	10	2
1143	S	3	19	37
1143	S	3	8	18
1143	S	3	2	16
1143	S	3	5	13
1143	S	3	30	2
1143	S	3	17	2
1163	M	2	8	384
1163	M	2	55	216
1163	M	2	38	32
1163	M	2	35	12
1163	M	2	4	2
1163	M	2	61	2
1163	M	2	22	1
1199	M	2	43	560
1199	M	2	26	39
1199	M	2	23	12
1199	M	2	49	3
1199	M	2	25	1
1199	M	2	11	1
1199	M	2	10	1
1241	S	2	29	165
1241	S	2	12	30
1241	M	2	29	6
1241	W	2	29	3
1241	S	2	35	2
1241	S	2	11	1
1245	W	3	8	39
1265	W	2	21	469
1265	W	2	4	6
1265	W	2	27	6
1265	W	2	3	1
1298	M	2	10	590
1298	M	2	16	10
1301	M	2	9	606
1301	M	2	15	8
1301	W	2	9	2

1304	M	2	8	318
1304	M	2	14	4
1399	W	1	3	694

Table S32. Length and count data for influenza A putative ORFs accompanying Figure S30 (N4).

Position	Kozak	Frame	Length	Count
1	W	1	470	188
87	W	3	5	134
87	W	3	15	1
96	S	3	2	144
96	S	3	5	29
96	S	3	25	12
96	S	3	12	2
118	W	1	431	9
131	W	2	29	31
131	M	2	29	1
131	W	2	17	1
141	M	3	10	24
141	M	3	9	1
170	M	2	6	107
170	M	2	16	44
170	M	2	4	28
170	M	2	36	9
185	W	2	11	69
185	W	2	31	9
194	W	2	8	114
194	W	2	28	6
239	W	2	13	83
241	S	1	390	6
241	W	1	390	4
270	W	3	15	12
270	W	3	35	12
270	W	3	21	7
270	W	3	19	6
270	W	3	12	4
270	W	3	3	2
285	S	3	10	60
285	S	3	5	18
285	S	3	30	13
285	S	3	16	7
285	S	3	7	3
308	S	2	30	175
308	S	2	13	5
366	M	3	4	97
366	M	3	3	46

366	M	3	18	5
377	W	2	7	67
381	W	3	13	131
381	W	3	41	26
381	W	3	12	15
419	W	2	8	161
419	W	2	17	1
434	M	2	3	142
434	M	2	12	1
472	W	1	313	188
531	S	3	6	135
531	S	3	7	9
546	W	3	15	30
546	W	3	2	9
546	W	3	52	5
546	M	3	2	2
554	S	2	5	43
593	W	2	4	103
593	W	2	7	31
593	W	2	6	1
623	S	2	2	144
623	S	2	7	35
623	S	2	32	8
667	M	1	248	188
687	W	3	5	55
687	W	3	46	1
693	W	3	3	112
693	W	3	21	1
701	M	2	6	183
701	M	2	21	2
701	M	2	7	1
701	S	2	6	1
721	W	1	230	25
728	M	2	12	81
740	W	2	8	118
740	W	2	10	9
740	M	2	8	1
787	M	1	208	11
787	S	1	208	6
793	M	1	206	122
793	S	1	206	2
793	W	1	206	2
800	M	2	36	143

800	M	2	4	1
806	W	2	34	141
806	W	2	78	23
806	W	2	59	4
806	W	2	2	1
831	M	3	10	51
831	M	3	21	6
831	M	3	6	2
857	W	2	17	38
857	M	2	61	22
857	M	2	17	14
857	M	2	42	5
864	W	3	3	34
864	W	3	19	8
903	S	3	6	105
903	S	3	8	8
944	M	2	23	110
944	M	2	32	52
944	M	2	16	9
944	M	2	13	4
982	M	1	143	11
986	M	2	9	109
986	M	2	18	67
986	M	2	2	9
999	M	3	6	6
999	W	3	6	4
999	M	3	19	4
999	W	3	19	2
1004	S	2	12	11
1019	S	2	7	158
1034	S	2	2	134
1061	S	2	6	188
1067	S	2	4	185
1120	S	1	97	181
1120	M	1	97	7
1130	M	2	18	157
1130	M	2	17	1
1136	M	2	16	176
1136	M	2	15	1
1140	M	3	16	104
1140	M	3	29	27
1140	M	3	17	18
1140	M	3	5	4

1140	M	3	18	2
1166	M	2	6	186
1166	M	2	5	1
1190	M	2	30	61
1263	M	3	4	100
1263	W	3	4	43
1263	M	3	20	1
1279	M	1	44	188
1311	M	3	4	123
1311	M	3	2	64
1311	M	3	5	1
1371	S	3	9	134
1371	S	3	2	38
1379	M	2	8	188

Table S33. Length and count data for influenza A putative ORFs accompanying Figure S31 (N5).

Position	Kozak	Frame	Length	Count
1	W	1	473	167
74	W	2	4	32
158	M	2	3	165
158	M	2	8	2
269	M	2	6	138
269	M	2	24	2
269	M	2	5	1
302	S	2	13	136
302	S	2	4	25
302	S	2	2	6
329	M	2	4	153
360	M	3	6	138
360	M	3	18	12
375	M	3	13	12
413	W	2	8	167
466	W	1	318	167
494	W	2	25	152
494	W	2	23	12
494	W	2	27	1
525	M	3	7	167
540	M	3	2	159
540	W	3	2	6
545	M	2	8	154
545	M	2	6	12
545	M	2	10	1
548	S	2	7	154
548	S	2	5	12
548	S	2	9	1
558	S	3	10	77
587	W	2	6	141
587	W	2	17	25
593	M	2	4	141
593	M	2	15	25
599	W	2	2	142
599	W	2	13	25
614	S	2	8	163
614	S	2	7	4
622	M	1	266	136
632	W	2	2	156

645	S	3	33	144
645	S	3	30	15
645	S	3	29	6
645	S	3	41	1
645	S	3	47	1
681	M	3	21	118
681	M	3	18	15
681	M	3	17	6
681	M	3	29	1
687	W	3	19	34
687	W	3	16	1
691	W	1	243	153
691	M	1	243	1
695	M	2	6	25
715	M	1	235	167
722	M	2	19	153
722	M	2	18	12
722	M	2	55	2
767	W	2	4	12
767	W	2	3	1
775	S	1	215	151
785	W	2	34	129
785	W	2	22	1
785	W	2	20	1
825	M	3	8	16
858	M	3	5	33
858	M	3	11	1
881	S	2	2	150
886	M	1	178	167
911	W	2	3	135
911	W	2	44	26
926	M	2	39	147
926	M	2	2	8
942	W	3	14	140
942	W	3	42	9
942	W	3	21	4
942	W	3	43	1
1007	W	2	12	157
1037	S	2	2	165
1093	M	1	109	10
1139	S	2	34	10
1139	S	2	9	1
1240	M	1	60	167

1254	M	3	24	123
1254	M	3	25	7
1266	W	3	20	157
1266	W	3	21	7
1282	M	1	46	167
1371	S	3	4	27

Table S34. Length and count data for influenza A putative ORFs accompanying Figure S32 (N6).

Position	Kozak	Frame	Length	Count
1	W	1	470	1182
24	M	3	40	765
24	M	3	29	354
24	M	3	43	36
24	M	3	69	9
24	M	3	32	9
24	M	3	52	4
24	M	3	84	1
43	M	1	456	1125
43	S	1	456	4
148	M	1	421	288
152	W	2	8	1011
152	M	2	8	24
152	S	2	8	4
152	W	2	20	4
223	M	1	396	101
247	M	1	388	748
288	M	3	5	405
288	M	3	10	337
288	M	3	13	207
288	M	3	54	70
288	M	3	31	69
288	M	3	28	64
288	M	3	44	12
288	M	3	7	7
288	M	3	33	7
311	M	2	4	812
311	M	2	2	327
311	M	2	54	1
332	M	2	29	691
332	M	2	4	219
332	M	2	47	81
332	M	2	23	13
369	W	3	4	595
369	W	3	27	27
380	W	2	13	513
380	W	2	31	80
380	W	2	7	5
391	M	1	340	1181

431	M	2	14	808
431	M	2	15	356
431	M	2	5	4
437	M	2	12	816
437	M	2	13	318
437	M	2	3	4
449	W	2	8	808
449	W	2	9	352
449	M	2	9	1
487	S	1	308	1179
487	M	1	308	2
512	M	2	3	365
512	M	2	6	313
512	M	2	53	12
512	M	2	34	1
512	M	2	42	1
525	M	3	10	803
525	W	3	24	182
525	W	3	10	65
525	W	3	35	30
525	W	3	25	11
525	W	3	49	6
525	W	3	23	3
525	M	3	5	3
525	M	3	24	2
525	W	3	59	1
525	M	3	23	1
534	S	3	7	446
534	M	3	7	429
534	S	3	21	182
534	S	3	32	31
534	S	3	46	23
534	M	3	22	11
534	S	3	56	5
534	S	3	20	3
534	M	3	2	2
534	M	3	21	2
534	S	3	2	2
534	M	3	20	1
549	W	3	2	817
549	M	3	16	182
549	M	3	2	48
549	M	3	27	38

549	M	3	41	24
549	M	3	17	11
549	M	3	51	4
549	M	3	15	3
549	W	3	16	2
549	W	3	15	1
554	M	2	39	754
554	M	2	28	130
554	M	2	20	26
554	M	2	31	2
554	M	2	55	1
557	S	2	27	298
557	S	2	38	21
557	S	2	19	19
557	S	2	9	4
557	S	2	10	1
557	S	2	54	1
557	S	2	3	1
571	M	1	280	1176
571	W	1	280	5
579	M	3	6	199
579	M	3	17	57
579	M	3	41	45
579	M	3	31	27
579	M	3	7	14
579	W	3	6	7
579	M	3	5	6
579	W	3	7	4
579	W	3	17	2
579	M	3	60	2
583	W	1	276	965
602	M	2	23	756
602	M	2	12	369
602	M	2	4	41
602	M	2	15	2
602	M	2	39	1
626	S	2	15	551
626	S	2	4	39
626	S	2	7	2
626	M	2	15	2
654	M	3	16	250
654	S	3	6	233
654	S	3	16	107

654	M	3	11	93
654	S	3	31	13
654	M	3	29	7
654	S	3	3	4
654	S	3	35	2
654	S	3	11	2
654	S	3	30	1
654	M	3	30	1
690	M	3	4	956
690	W	3	4	134
690	M	3	19	13
690	M	3	17	7
690	W	3	17	6
690	M	3	18	2
690	M	3	23	2
696	W	3	2	826
696	W	3	15	11
696	W	3	17	3
696	W	3	16	3
696	W	3	21	2
704	S	2	6	752
704	S	2	5	32
704	S	2	7	1
711	M	3	11	426
711	M	3	10	370
711	M	3	12	21
711	M	3	6	17
711	M	3	51	4
711	M	3	16	2
724	M	1	229	1179
731	M	2	11	1163
731	M	2	25	2
731	M	2	8	1
815	M	2	15	52
815	M	2	13	49
815	M	2	4	11
815	M	2	31	1
834	M	3	10	505
834	M	3	22	223
834	M	3	52	28
834	M	3	30	10
834	M	3	14	4
834	M	3	16	2

834	M	3	46	1
840	W	3	8	266
840	W	3	20	148
840	W	3	28	10
840	W	3	44	6
840	W	3	14	3
840	W	3	12	2
845	S	2	5	1030
845	S	2	21	75
845	S	2	3	49
859	M	1	184	458
859	S	1	184	12
867	W	3	11	800
867	W	3	5	125
867	W	3	3	112
867	W	3	41	97
867	W	3	19	11
867	W	3	35	5
873	W	3	9	227
873	W	3	39	102
873	W	3	17	11
873	W	3	33	6
873	W	3	3	2
873	W	3	38	1
928	M	1	161	336
931	M	1	160	1172
931	W	1	160	10
989	W	2	11	1077
989	W	2	3	3
989	W	2	20	1
1001	M	2	7	297
1001	M	2	22	1
1013	M	2	3	1137
1013	S	2	3	14
1013	M	2	12	1
1070	M	2	10	750
1070	M	2	21	125
1070	M	2	6	15
1070	W	2	10	7
1083	M	3	6	343
1083	M	3	14	8
1083	M	3	2	2
1083	M	3	5	1

1124	M	2	3	861
1124	M	2	2	2
1129	M	1	94	1136
1145	W	2	14	1173
1145	W	2	3	6
1145	W	2	15	1
1186	W	1	75	109
1254	M	3	18	577
1254	M	3	13	96
1254	M	3	2	20
1254	M	3	8	5
1254	M	3	15	3
1254	M	3	25	1
1274	W	2	3	1111
1274	W	2	20	1
1371	S	3	4	358
1371	S	3	7	10
1377	S	3	2	695
1377	S	3	5	7

Table S35. Length and count data for influenza A putative ORFs accompanying Figure S33 (N7).

Position	Kozak	Frame	Length	Count
1	W	1	470	471
83	W	2	3	464
83	W	2	9	5
95	W	2	5	453
122	M	2	11	155
144	M	3	12	200
144	M	3	24	196
144	M	3	17	11
144	M	3	7	7
144	M	3	23	7
144	M	3	18	1
191	W	2	19	470
191	W	2	14	1
250	W	1	387	450
267	W	3	3	399
267	W	3	10	1
282	S	3	13	313
282	S	3	5	154
282	S	3	7	2
305	M	2	2	437
305	M	2	10	10
356	W	2	6	438
356	W	2	37	32
385	M	1	342	470
389	W	2	26	61
431	M	2	12	30
443	W	2	8	85
528	S	3	7	125
528	S	3	4	39
528	M	3	4	1
548	M	2	6	470
548	M	2	3	1
565	W	1	282	467
565	M	1	282	4
590	M	2	17	433
590	M	2	6	1
596	M	2	15	32
617	M	2	8	467
617	S	2	8	2

648	S	3	6	465
648	S	3	11	2
684	M	3	13	123
684	W	3	69	120
684	M	3	39	83
684	M	3	70	72
684	W	3	18	33
684	W	3	70	13
684	M	3	29	8
684	W	3	74	7
684	W	3	13	6
684	W	3	62	1
684	W	3	54	1
690	W	3	11	129
690	W	3	67	119
690	W	3	68	87
690	W	3	37	83
690	W	3	16	35
690	W	3	27	8
690	W	3	72	7
690	W	3	52	1
690	W	3	60	1
698	S	2	4	460
698	S	2	5	8
705	M	3	6	129
705	M	3	62	119
705	M	3	63	87
705	M	3	32	83
705	M	3	11	11
705	M	3	22	8
705	M	3	67	7
705	M	3	47	1
705	M	3	55	1
718	M	1	231	471
725	M	2	18	456
725	M	2	19	12
828	M	3	21	144
828	M	3	22	89
828	M	3	6	9
828	M	3	26	9
828	M	3	59	6
828	M	3	14	1
834	W	3	20	269

834	W	3	19	112
834	W	3	24	7
834	W	3	4	6
834	W	3	12	2
839	S	2	6	465
839	S	2	21	3
867	W	3	9	294
867	W	3	8	148
867	W	3	13	10
867	W	3	46	2
867	W	3	3	1
916	M	1	165	464
916	S	1	165	7
920	W	2	58	275
920	W	2	25	160
920	W	2	47	27
920	W	2	2	2
920	W	2	24	1
920	W	2	54	1
925	S	1	162	468
925	M	1	162	2
948	W	3	6	420
948	W	3	19	18
994	M	1	139	219
994	S	1	139	34
1077	M	3	14	471
1123	M	1	96	470
1139	W	2	15	466
1139	W	2	3	2
1139	M	2	15	1
1139	W	2	9	1
1238	M	2	14	363
1247	M	2	11	42
1271	W	2	3	465
1310	M	2	8	462

Table S36. Length and count data for influenza A putative ORFs accompanying Figure S34 (N8).

Position	Kozak	Frame	Length	Count
1	W	1	470	1362
62	W	2	6	96
62	W	2	5	87
62	W	2	9	13
62	W	2	8	1
74	W	2	2	89
74	W	2	5	22
74	W	2	6	3
116	S	2	18	704
116	S	2	23	277
116	S	2	4	74
116	M	2	4	11
116	S	2	10	8
116	M	2	18	5
116	S	2	11	4
116	M	2	9	2
116	M	2	10	1
116	M	2	23	1
116	S	2	8	1
116	S	2	9	1
125	S	2	15	625
125	S	2	20	272
125	S	2	7	92
125	M	2	8	8
125	S	2	6	6
125	M	2	20	5
125	S	2	8	5
125	M	2	15	4
125	M	2	7	1
125	S	2	5	1
137	M	2	11	699
137	M	2	16	263
137	S	2	11	26
137	M	2	3	21
137	S	2	4	9
137	M	2	2	4
137	S	2	3	2
137	S	2	16	1
161	M	2	3	952

161	M	2	8	281
161	M	2	5	47
161	S	2	3	1
192	S	3	7	839
192	M	3	2	180
192	S	3	4	178
192	M	3	4	102
192	S	3	2	22
192	M	3	7	10
192	S	3	3	2
192	S	3	15	1
192	S	3	26	1
192	M	3	14	1
206	W	2	5	950
206	W	2	14	6
206	M	2	5	2
233	M	2	5	77
233	S	2	5	11
233	M	2	26	2
236	W	2	4	179
236	M	2	4	118
236	S	2	4	20
236	W	2	35	2
247	W	1	388	1339
272	M	2	13	1150
272	M	2	24	2
305	S	2	2	497
305	S	2	12	180
332	M	2	4	1104
332	M	2	3	191
332	M	2	36	4
332	M	2	26	2
363	M	3	6	105
363	W	3	6	76
378	M	3	13	187
378	W	3	12	8
378	M	3	12	5
416	W	2	8	1103
416	W	2	17	212
416	M	2	8	1
431	M	2	3	934
431	M	2	12	223
469	W	1	314	1362

497	W	2	9	909
497	W	2	23	283
497	W	2	27	2
528	S	3	7	507
528	S	3	20	48
528	S	3	35	8
528	S	3	22	7
528	S	3	21	4
528	M	3	7	1
543	W	3	2	960
543	M	3	2	177
543	W	3	15	29
543	M	3	17	4
543	M	3	16	4
543	W	3	30	3
543	M	3	15	2
548	M	2	6	1215
548	M	2	10	2
551	S	2	5	1183
551	S	2	9	4
551	M	2	5	1
561	S	3	9	1012
561	S	3	24	135
561	S	3	11	25
561	S	3	10	24
561	M	3	9	2
561	S	3	27	2
561	S	3	4	1
565	W	1	282	1298
565	M	1	282	3
590	W	2	4	579
590	W	2	16	457
590	W	2	6	10
617	S	2	7	1047
617	S	2	16	285
617	S	2	15	8
617	S	2	8	2
617	S	2	12	1
635	W	2	10	66
635	W	2	9	5
635	W	2	2	2
635	W	2	6	1
648	M	3	16	177

648	M	3	18	5
648	M	3	25	4
648	M	3	40	1
684	M	3	6	1051
684	M	3	4	179
684	M	3	13	4
684	M	3	28	4
705	W	3	30	441
705	W	3	21	230
705	W	3	11	51
705	W	3	16	5
705	W	3	27	4
705	W	3	19	3
705	W	3	6	2
705	W	3	47	1
718	M	1	231	1362
725	M	2	19	1281
773	S	2	3	196
809	M	2	4	1214
809	M	2	76	74
809	M	2	32	27
809	M	2	33	6
809	M	2	104	2
809	W	2	16	2
809	M	2	16	1
828	M	3	6	873
828	M	3	5	140
828	M	3	8	8
834	W	3	4	914
834	W	3	3	117
834	W	3	6	8
845	W	2	21	912
845	W	2	92	283
845	W	2	64	71
845	W	2	20	45
845	W	2	4	25
845	W	2	57	1
845	W	2	19	1
845	W	2	91	1
848	S	2	63	77
848	S	2	19	24
848	S	2	3	8
848	S	2	20	1

861	M	3	3	748
861	M	3	11	146
861	M	3	34	12
861	M	3	41	5
861	M	3	19	3
861	W	3	3	1
861	M	3	23	1
867	W	3	9	178
867	W	3	3	3
945	W	3	6	450
945	W	3	13	255
945	W	3	5	17
945	W	3	21	8
945	W	3	40	1
945	W	3	48	1
983	M	2	18	100
983	S	2	46	5
983	M	2	11	1
994	M	1	139	103
1002	M	3	2	912
1002	W	3	7	160
1002	W	3	2	84
1002	M	3	12	32
1002	W	3	21	22
1002	W	3	12	2
1002	M	3	21	1
1002	M	3	29	1
1015	M	1	132	1104
1031	S	2	30	966
1031	S	2	2	111
1031	S	2	15	16
1031	S	2	49	3
1031	S	2	53	1
1031	S	2	29	1
1064	M	2	19	1040
1064	M	2	18	39
1064	M	2	4	24
1064	M	2	42	1
1064	M	2	14	1
1067	M	2	18	1008
1067	W	2	18	91
1067	M	2	3	24
1067	W	2	17	9

1067	W	2	9	5
1067	M	2	17	4
1067	M	2	37	3
1067	M	2	41	1
1071	M	3	6	1096
1071	M	3	14	169
1071	M	3	7	1
1075	M	1	112	1139
1133	S	2	19	673
1133	S	2	15	331
1133	S	2	14	195
1133	S	2	10	94
1133	S	2	34	32
1133	S	2	16	6
1133	S	2	9	2
1133	S	2	3	1
1133	S	2	32	1
1133	S	2	11	1
1184	W	2	17	103
1184	W	2	2	2
1184	W	2	31	1
1248	M	3	8	178
1248	W	3	8	1
1260	W	3	4	999
1260	W	3	20	5
1276	M	1	45	1357
1276	S	1	45	1
1308	S	3	4	878
1308	S	3	3	188
1308	M	3	4	121
1308	S	3	14	60
1308	S	3	29	1
1333	M	1	26	1362
1368	S	3	9	854
1368	S	3	12	4
1368	S	3	5	2
1368	S	3	2	2
1368	S	3	3	1

Table S37. Length and count data for influenza A putative ORFs accompanying Figure S35 (N9).

Position	Kozak	Frame	Length	Count
1	W	1	470	421
24	W	3	40	333
24	W	3	33	42
24	W	3	54	33
24	W	3	36	5
24	W	3	31	2
24	W	3	51	1
24	W	3	29	1
24	W	3	55	1
155	W	2	5	227
155	M	2	5	26
188	M	2	7	303
188	M	2	2	1
229	M	1	394	360
291	M	3	28	208
291	M	3	31	118
291	M	3	10	55
291	M	3	16	7
291	M	3	7	1
302	M	2	6	325
302	M	2	15	64
314	M	2	2	353
314	M	2	11	65
341	W	2	2	370
341	W	2	45	11
341	W	2	3	1
365	W	2	37	396
365	W	2	19	12
365	W	2	38	6
365	W	2	13	5
372	W	3	4	118
383	W	2	31	395
383	W	2	13	7
383	W	2	32	6
383	W	2	7	5
383	M	2	13	4
383	M	2	31	2
387	W	3	23	107
387	W	3	22	13

387	W	3	6	7
387	W	3	12	5
398	W	2	26	397
398	W	2	8	12
398	W	2	27	6
398	W	2	2	5
440	M	2	12	304
452	W	2	8	25
452	W	2	9	4
528	M	3	7	357
528	M	3	10	9
528	M	3	5	2
537	M	3	4	50
537	M	3	7	13
557	M	2	20	399
557	M	2	39	9
560	S	2	19	407
560	S	2	38	10
574	M	1	279	412
574	W	1	279	9
582	M	3	16	275
582	M	3	23	104
582	M	3	6	13
582	M	3	36	9
582	W	3	23	6
582	W	3	36	4
582	M	3	15	4
582	M	3	7	3
582	M	3	28	1
582	W	3	5	1
582	M	3	5	1
605	M	2	4	406
605	M	2	23	9
621	S	3	10	71
621	S	3	15	1
657	M	3	11	322
657	M	3	2	11
657	M	3	3	1
657	M	3	40	1
657	M	3	55	1
693	W	3	28	207
693	W	3	43	178
693	W	3	4	17

693	W	3	46	5
693	W	3	13	4
693	W	3	21	4
699	W	3	26	202
699	W	3	41	162
699	W	3	2	17
699	W	3	11	6
699	W	3	44	5
699	W	3	19	4
707	S	2	5	26
707	S	2	34	3
707	S	2	29	3
714	W	3	36	113
714	M	3	6	10
714	W	3	39	5
714	W	3	14	4
714	W	3	21	2
714	M	3	39	1
734	M	2	20	407
734	M	2	19	2
734	M	2	14	1
798	S	3	8	387
798	S	3	11	16
798	M	3	11	9
798	S	3	29	3
798	M	3	8	2
837	M	3	16	299
837	M	3	22	91
837	M	3	14	17
843	W	3	14	310
843	W	3	20	77
843	W	3	12	7
848	S	2	21	40
848	S	2	6	9
848	S	2	4	1
876	W	3	3	120
876	W	3	9	74
934	M	1	159	420
934	S	1	159	1
957	W	3	12	127
957	W	3	7	2
992	W	2	4	195
992	W	2	32	10

1016	M	2	24	258
1016	M	2	17	33
1016	M	2	11	3
1016	S	2	24	1
1016	M	2	28	1
1043	S	2	15	280
1043	S	2	8	51
1043	S	2	19	6
1043	S	2	2	4
1070	M	2	6	404
1070	M	2	10	11
1070	W	2	6	2
1124	M	2	3	142
1129	M	1	94	418
1145	W	2	2	419
1157	W	2	13	135
1157	W	2	42	5
1157	W	2	11	5
1157	W	2	10	3
1231	M	1	60	395
1254	M	3	24	47
1254	M	3	15	7
1254	M	3	25	3
1254	M	3	18	2
1254	M	3	2	2
1266	M	3	14	23
1266	M	3	4	11
1266	M	3	21	11
1266	M	3	20	2
1274	W	2	3	181
1314	S	3	4	202
1314	S	3	5	76
1314	S	3	3	1
1342	W	1	23	421
1371	S	3	4	384
1371	S	3	7	11
1385	M	2	4	421

Table S38. Length and count data for influenza A putative ORFs accompanying Figure S36 (N10).

Position	Kozak	Frame	Length	Count
1	W	1	442	3
21	M	3	38	2
21	M	3	47	1
50	W	2	2	2
58	M	1	423	3
79	W	1	416	3
96	M	3	13	2
96	M	3	22	1
161	W	2	6	3
250	W	1	359	3
341	W	2	5	1
424	S	1	301	3
429	S	3	42	1
490	M	1	279	3
519	M	3	12	3
537	S	3	6	3
557	M	2	8	3
583	M	1	248	3
593	S	2	4	3
660	M	3	13	3
737	W	2	13	3
749	S	2	9	2
765	S	3	11	2
860	M	2	18	3
863	M	2	17	3
926	S	2	53	2
926	S	2	11	1
979	S	1	116	3
1073	S	2	4	3
1136	M	2	33	3
1261	M	1	22	3

Table S39.Length and count data for influenza A putative ORFs accompanying Figure S37 (N11).

Position	Kozak	Frame	Length	Count
1	W	1	447	1
21	M	3	35	1
45	M	3	27	1
96	S	3	10	1
182	W	2	5	1
290	M	2	21	1
341	W	2	4	1
349	W	1	331	1
412	S	1	310	1
463	W	1	293	1
467	W	2	4	1
551	M	2	16	1
554	S	2	15	1
611	M	2	4	1
647	M	2	4	1
684	W	3	2	1
689	M	2	24	1
692	S	2	23	1
719	M	2	14	1
767	M	2	28	1
855	M	3	8	1
878	M	2	53	1
881	M	2	52	1
939	M	3	12	1
1036	W	1	102	1
1211	W	2	27	1
1286	W	2	2	1
1321	W	1	7	1

Table S40. An abridged ORF list of influenza HA and NA genes.

Gene	Position (Frame)	Dominant length (aa)	Prevalence (%)
H1	311 (2)	14	95.4
	498 (3)	3	82.1
H2	158 (2)	6	78.42
	481 (1)	402	99.79
H3	278 (2)	34	90.8
	297 (3)	5	78.29
	446 (2)	18	53.84
H4	109 (1)	528	99.23
	285 (3)	17	72.72
	380 (2)	29	76.9
	434 (2)	11	96.92
	551 (2)	31	68.98
H5	106 (1)	533	99.8
	273 (3)	12	93.4
	492 (3)	17	91.07
H6	308 (2)	8	96.87
H7	391 (1)	430	99.33
H8	139 (1)	520	100
	557 (2)	28	91.3
H9	167 (2)	14	86.36
	480 (3)	18	57.88
H10	226 (1)	486	98.62
	270 (3)	21	75.83
	358 (1)	442	100
	449 (2)	9	75.64
H11	273 (3)	22	63.87
	487 (1)	403	99.74
H12	276 (3)	20	76.61
	307 (1)	462	97.58
	492 (3)	14	92.74
H13	119 (2)	10	76.32
	231 (3)	21	55.26
	347 (2)	18	57.89
H14	431 (2)	12	100
	482 (2)	6	54.55
	533 (2)	41	100
H15	477 (3)	7	100
H16	122 (2)	4	67.74
	350 (2)	10	100
H17	167 (2)	6	66.67

	279 (3)	7	100
H18	155 (2)	6	100
	302 (2)	11	100
N1	96 (3)	2	94.31
N3	437 (2)	12	98
N4	96 (3)	2	76.6
	308 (2)	30	93.09
N5	302 (2)	13	81.44
N7	282 (3)	13	66.45
N8	116 (2)	18	52.06
	192 (3)	7	62.33
N10	424 (1)	301	100
N11	96 (3)	10	100
	412 (1)	310	100

The filtering criteria used in Table 2, strong Kozak consensus sequence, an upstream AUG location within the upstream third of the transcript and high prevalence (≥95% and ≥50% for in-frame and alternative-frame ORFs, respectively), were applied to obtain the most probable HA and NA ORF candidates from Figs. S9–S37 (Tables S11–S39). Because some of the genes have multiple frame-2 or frame-3 ORFs, we did not name the ORFs according to Table 2. Instead, their starting positions are provided for identification purposes.

Table S41. HHblits hits against PDB database for alternative-frame ORFs.

<table>
<tr><th>Query (Count) / Frame No.</th><th>Avg. E-value</th><th>Avg. identity (%)</th><th>Avg. prob. (%)</th></tr>
<tr><th colspan="4">PDB ID: Definition / Classification / Organism / Molecule Name</th></tr>
<tr><th colspan="4">One representative alignment</th></tr>
<tr><td>N4-381 (4) / Frame-3</td><td>1.20E-04</td><td>75.0</td><td>95.5</td></tr>
<tr><td colspan="4">1EYO: Solution structure of conotoxin tviia from conus tulipa / Toxin / Conus tulipa / Conotoxin tviia</td></tr>
<tr><td colspan="4">N4-381 6 LDSRCPPQ 13 (13)
Consensus 6 ldsrcppq 13 (13)
-|||||||
Consensus 5 r~srcpp~ 12 (30)
1EYO 5 RDSRCPPV 12 (30)</td></tr>
<tr><td>NP-987 (1) / Frame-3</td><td>1.50E-07</td><td>75.0</td><td>97.3</td></tr>
<tr><td colspan="4">[a]1CLV: yellow meal worm alpha-amylase in complex with the amaranth alpha-amylase inhibitor / Hydrolase / Tenebrio molitor / protein (alpha-amylase)</td></tr>
<tr><td colspan="4">NP-987 1 MDGMPCCS 8 (9)
Consensus 1 mdg~pcc~ 8 (9)
|||+|||+
Consensus 12 mdgvpcce 19 (32)
1CLV 12 MDGVPCCE 19 (32)</td></tr>
<tr><td>N7-684 (6) / Frame-3</td><td>7.65E-10</td><td>55.0</td><td>98.1</td></tr>
<tr><td colspan="4">[a]1CLV: (Same as above)</td></tr>
<tr><td colspan="4">N7-684 2 CMPQWNMCGNN 12 (13)
Consensus 2 c~p~wn~cg~n 12 (13)
|||+||||||.
Consensus 1 cipkwnrcgpk 11 (32)
1CLV 1 CIPKWNRCGPK 11 (32)</td></tr>
<tr><td>PB1-1995 (1) / Frame-3</td><td>8.70E-09</td><td>50.0</td><td>97.8</td></tr>
<tr><td colspan="4">4KT3: Structure of a type VI secretion system effector-immunity complex from Pseudomonas protegens / Hydrolase / Pseudomonas protegens Pf-5 / Uncharacterized protein</td></tr>
</table>

<table>
<tr><td colspan="4">PB1-1995 1 MDPQGKPFHPQYQP 14 (18)
Consensus 1 ~D~qGkpfHpgYy~ 14 (18)
||+||||||||||.
Consensus 51 ~d~~G~p~hpGyy~ 64 (137)
4KT3 51 QDENGKPPHPGYYD 64 (137)</td></tr>
<tr><td>NS1-50 (6) / Frame-2</td><td>6.87E-09</td><td>37.0</td><td>97.8</td></tr>
<tr><td colspan="4">3IJW: Crystal structure of BA2930 in complex with CoA / Transferase / Bacillus anthracis str. Ames / Aminoglycoside N3-acetyltransferase</td></tr>
<tr><td colspan="4">NS1-50 1 MSANGLQTGDLVMLHSSTG 19 (26)
Consensus 1 L~~LGI~~Gd~llVHsSl~ 19 (26)
+++|||++||+|+|||||.
Consensus 24 L~~LGi~~Gd~llVHsSl~ 42 (268)
3IJW 24 LRKLGLKKGMTVIVHSSLS 42 (268)</td></tr>
<tr><td>H3-161 (1) / Frame-2</td><td>2.40E-04</td><td>44.0</td><td>95.0</td></tr>
<tr><td colspan="4">3G9R: Structure of the HIV-1 gp41 Membrane-Proximal Ectodomain Region in a Putative Prefusion Conformation / Viral Protein / Saccharomyces cerevisiae Human immunodeficiency virus 1 / Fusion complex of HIV-1 Envelope glycoprotein and Saccharomyces cerevisiae General control protein GCN4</td></tr>
<tr><td colspan="4">H3-161 2 LQNWFRISQWVKYARILI 19 (28)
Consensus 2 lqnwfrisqwvkyarili 19 (28)
|.|||.|+.|+.|-+|.+
Consensus 8 LWnWFdIT~WlwYIKIfi 25 (42)
3G9R 8 LWNWFNITNWLWYIKIEE 25 (42)</td></tr>
<tr><td>H13-623 (5) / Frame-2</td><td>1.60E-07</td><td>43.0</td><td>97.2</td></tr>
<tr><td colspan="4">2WM1: the crystal structure of human alpha-amino-beta-carboxymuconate-epsilon-semialdehyde decarboxylase in complex with 1,3- dihydroxyacetonephosphate suggests a regulatory link between nad synthesis and glycolysis / Lyase / Homo sapiens / 2-amino-3-carboxymuconate-6-semialdehyde decarboxylase</td></tr>
<tr><td colspan="4">H13-623 1 MSIVIHTHWFPPSL 14 (20)
Consensus 1 MiID~H~Hi~pp~~ 14 (20)
|+||+|+|++||++</td></tr>
</table>

<table>
<tr><td colspan="4">Consensus   1 m~ID~H~H~~p~~~   14 (336)
2WM1   1 MKIDIHSHILPKEW   14 (336)</td></tr>
<tr><td>H5-1479 (5), H6-1473 (1) / Frame-3</td><td>6.50E-09</td><td>40.0</td><td>97.8</td></tr>
<tr><td colspan="4">2DCV: The solution structure of horseshoe crab antimicrobial peptide tachystatin b with the inhibitory cystine-knot motif / Antimicrobial Protein / Tachypleus tridentatus / Tachystatin-B1</td></tr>
<tr><td colspan="4">H6-1473   1 MYGVCQKRNL   10 (10)
Consensus   1 ~yG~Cqrrn~   10 (10)
|||+||||||
Consensus 33 i~g~c~r~~~   42 (42)
2DCV   33 IFGTCSRRNF   42 (42)</td></tr>
<tr><td>PB1-1737 (6) / Frame-3</td><td>1.30E-09</td><td>36.0</td><td>98.0</td></tr>
<tr><td colspan="4">4A1S: Crystallographic structure of the Pins:Insc complex / Cell Cycle / Drosophila melanogaster / PARTNER OF INSCUTEABLE</td></tr>
<tr><td colspan="4">PB1-1737   3 ANPFQSWAAGLRWR   16 (17)
Consensus   3 ~~pvqsWl~eqRw~   16 (17)
-+||||||+||||+
Consensus   7 ~~~v~sWl~eqRw~   20 (40)
4ALS   7 PEPVASWMSEQRWA   20 (40)</td></tr>
<tr><td>NS1-86 (1) / Frame-2</td><td>2.40E-07</td><td>36.0</td><td>97.2</td></tr>
<tr><td colspan="4">2LI5: NMR structure of Atg8-Atg7C30 complex / Protein Transport / Saccharomyces cerevisiae S288c / Autophagy-related protein 8</td></tr>
<tr><td colspan="4">NS1-86   2 PHFLCGFAEIR   12 (14)
Consensus   2 PH~i~G~s~Ir   12 (14)
|||||||++++
Consensus   2 ~~~LSGL~~vq   12 (34)
2LI5   2 PHMISGLSVIK   12 (34)</td></tr>
<tr><td>PB1-993 (3) / Frame-3</td><td>3.30E-06</td><td>15.0</td><td>96.6</td></tr>
<tr><td colspan="4">1ROF: Crystal Structure of the Ferredoxin from Thauera aromatica / Electron Transport / Thauera aromatica K172 / ferredoxin</td></tr>
<tr><td colspan="4">PB1-993   3 QKCLEHCPYNVLK   15 (15)</td></tr>
</table>

<table>
<tr><td colspan="4">Consensus 3 ~~C~~~CP~~ai~ 15 (15)
+.|+++||++||+
Consensus 45 ~~C~~~CP~~Ai~ 57 (60)
1ROF 45 KDAADSCPTGAIS 57 (60)</td></tr>
<tr><td>[b]N2-831 (6) / Frame-3</td><td>9.90E-05</td><td>14.0</td><td>95.3</td></tr>
<tr><td colspan="4">1TP9: PRX D (type II) / Oxidoreductase / Populus trichocarpa / Peroxiredoxin</td></tr>
<tr><td colspan="4">N2-831 4 LSPISWCQMCLQRQLERLQQAHRRYKFK 31 (33)
Consensus 4 lspiswcqmclqrqlerlqqahrrykfk 31 (33)
..|-+||..|..+++..|++...+|+-+
Consensus 42 f~~~~~C~~C~~~~~~~~l~~~~~~~~~~~~ 69 (162)
1TP9 42 GVPGAFTPTCSLKHVPGFIEKAGELKSK 69 (162)</td></tr>
<tr><td>[b]N2-831 (1) / Frame-3</td><td>1.70E-04</td><td>14.0</td><td>95.0</td></tr>
<tr><td colspan="4">2R37: Crystal structure of human glutathione peroxidase 3 / Oxidoreductase / Homo sapiens / Glutathione peroxidase 3</td></tr>
<tr><td colspan="4">N2-831 3 LLSSISWCQMCLQRQLERLQSAHRRYKHKG 32 (33)
Consensus 3 llssiswcqmclqrqlerlqsahrrykhkg 32 (33)
+...-+||..| . ++..|+....+|+.+|
Consensus 43 l~F~at~C~~C-~-~~~~l~~l~~~~~~~~ 70 (207)
2R37 43 FVNVASYGGLT-G-QYIELNALQEELAPFG 70 (207)</td></tr>
</table>

This table supplements Table 4 for the hits of structural homologues to all frame-2 and -3 influenza A novel ORFs. E-value, sequence identity and probability were provided by averaging ones from HHblits hits. The alignments between putative ORFs and structures were generated by HHblits tool. The number in parenthesis following a sequence alignment is the length of either query or subject. Consensus sequences are shown in HMM format for both the query and the subject, in which capital and lowercase letters are used to represent conserved columns with ≥60 and ≥40 probabilities, respectively, and a tilde "~" is used to represent non-conserved column. Based on default settings with two iterations, HHblits tool adds significant hit(s) from the previous search/iteration to the HMM query for the next search/iteration. It results to that our query may not be single sequence. Five symbols are used to show the alignment quality, in which "|", "+", ".", "-" and "=" each represents a quality from the perfect to the worst.

[a] Two groups of putative ORFs from NP and N7 genes hit to 1CLV structure.

[b] One putative ORF, N2-281, hits to different structures.
