# Supplementary material for: Computational Analysis and Mapping of Novel Open Reading Frames in Influenza A Viruses
Source: PLoS One. 2014 Dec 15;9(12):e115016. doi: 10.1371/journal.pone.0115016 (PMC4266615; doi:10.1371/journal.pone.0115016)
Supplement: S2 File — S1–S37 Figures. ORF maps of PB2, NP, M1, M2, M42, NS1, NS2, NS3, H1–H18 and N1–N11 transcripts. Symbols and legends used are as defined in Figure 1. Briefly, the putative ORFs are graphed in 3 forward-reading frames: F1, F2 and F3. Each colored box represents the start codon of an ORF, with red, green and blue indicating strong, medium and weak Kozak sequences, respectively. Numbers from 1 indicate the positions of start codons. For ORFs with mixed populations of different Kozak strengths, multiple colors indicate the population proportions. F2 and F3 ORFs of major prevalence (>50%) are additionally labelled by their frequencies. ORFs of lower prevalence (<95% for F1, <50% for F2/F3) are grey-labelled. Each ORF ends with a solid line. For F2/F3 ORFs of various sizes, the corresponding dominant length is used to close the ORF. For overlapping ORFs, an asterisk (*) accompanying the start position is additionally labelled in grey to mark the end of the ORF. S38 Figure. Nucleotide alignments for the BLASTP reports. Four nt sequence alignments are used to illustrate the single nt insertions or deletions (red) that disturbed the reading frames, producing database hits to the ORFs (Table 3). (a) Corresponds to Case 5 in Table 3. Query = JN087010, which translates into AEI29961 in Table 3. Sbjct = HM145493, the second-closest hit in the NT database, excluding the query sequence itself. b) Corresponds to Case 6 in Table 3. Query = AY790308, which translates into AAV68025 in Table 3. Sbjct = AY862651, the second-closest hit in the NT database, excluding the query sequence itself. c) Corresponds to Case 7 in Table 3. Query = M25291, which translates into AAA43224 in Table 3. Sbjct = CY005956, the second-closest hit in the NT database, excluding the query sequence itself. d) Corresponds to Case 8 in Table 3. Query = EU429730, which translates into ACA04672 in Table 3. Sbjct = EU429760, the second-closest hit in the NT database, excluding the query sequence itself. (PDF) [file pone.0115016.s002.pdf]

Figures S1–S37. ORF maps of PB2, NP, M1, M2, M42, NS1, NS2, NS3, H1–H18 and N1–N11 transcripts. Symbols and legends used are as defined in Figure 1. Briefly, the putative ORFs are graphed in 3 forward-reading frames: F1, F2 and F3. Each colored box represents the start codon of an ORF, with red, green and blue indicating strong, medium and weak Kozak sequences, respectively. Numbers from 1 indicate the positions of start codons. For ORFs with mixed populations of different Kozak strengths, multiple colors indicate the population proportions. F2 and F3 ORFs of major prevalence (>50%) are additionally labelled by their frequencies. ORFs of lower prevalence (<95% for F1, <50% for F2/F3) are grey-labelled. Each ORF ends with a solid line. For F2/F3 ORFs of various sizes, the corresponding dominant length is used to close the ORF. For overlapping ORFs, an asterisk (*) accompanying the start position is additionally labelled in grey to mark the end of the ORF.

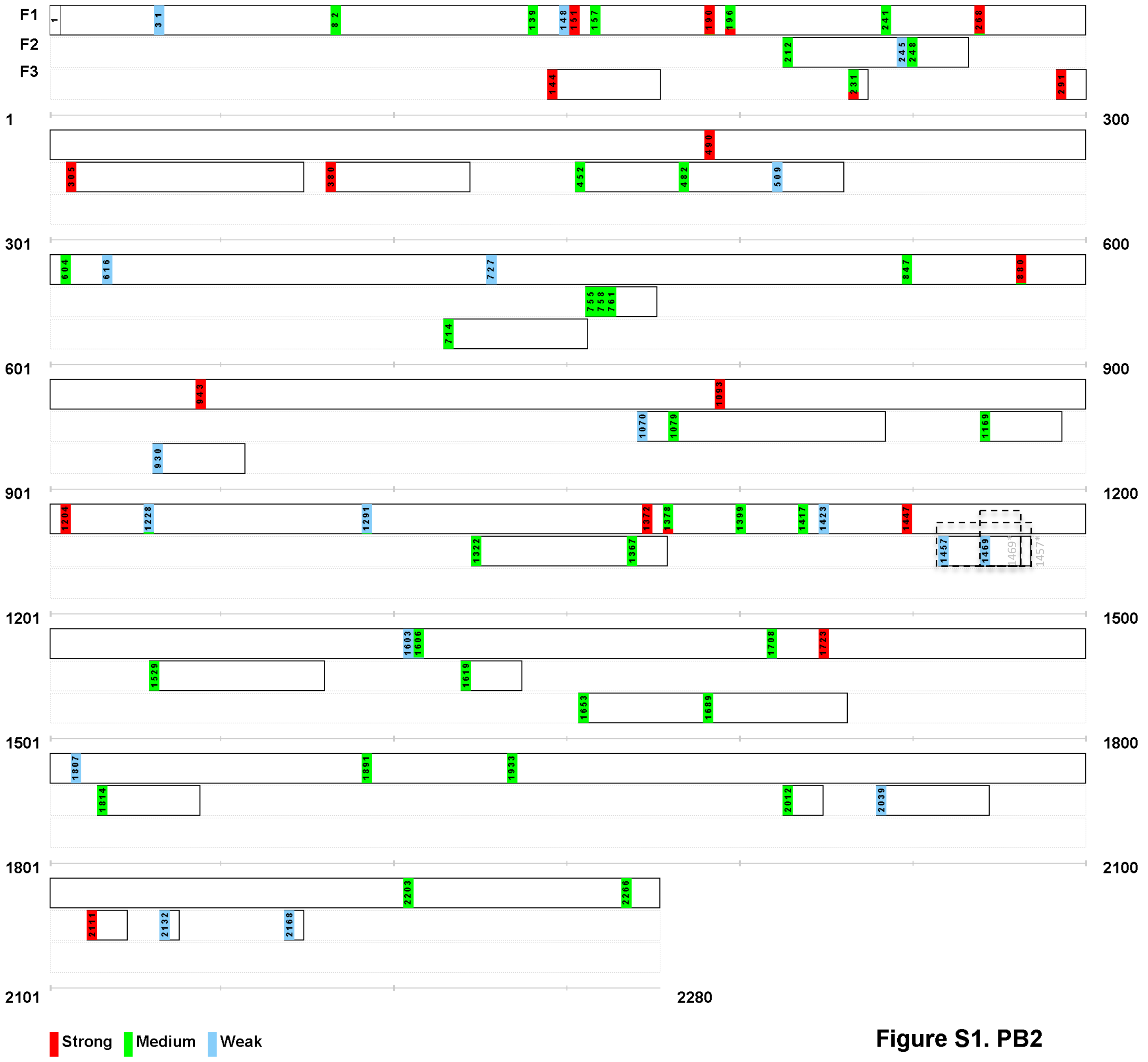

Figure S1. PB2

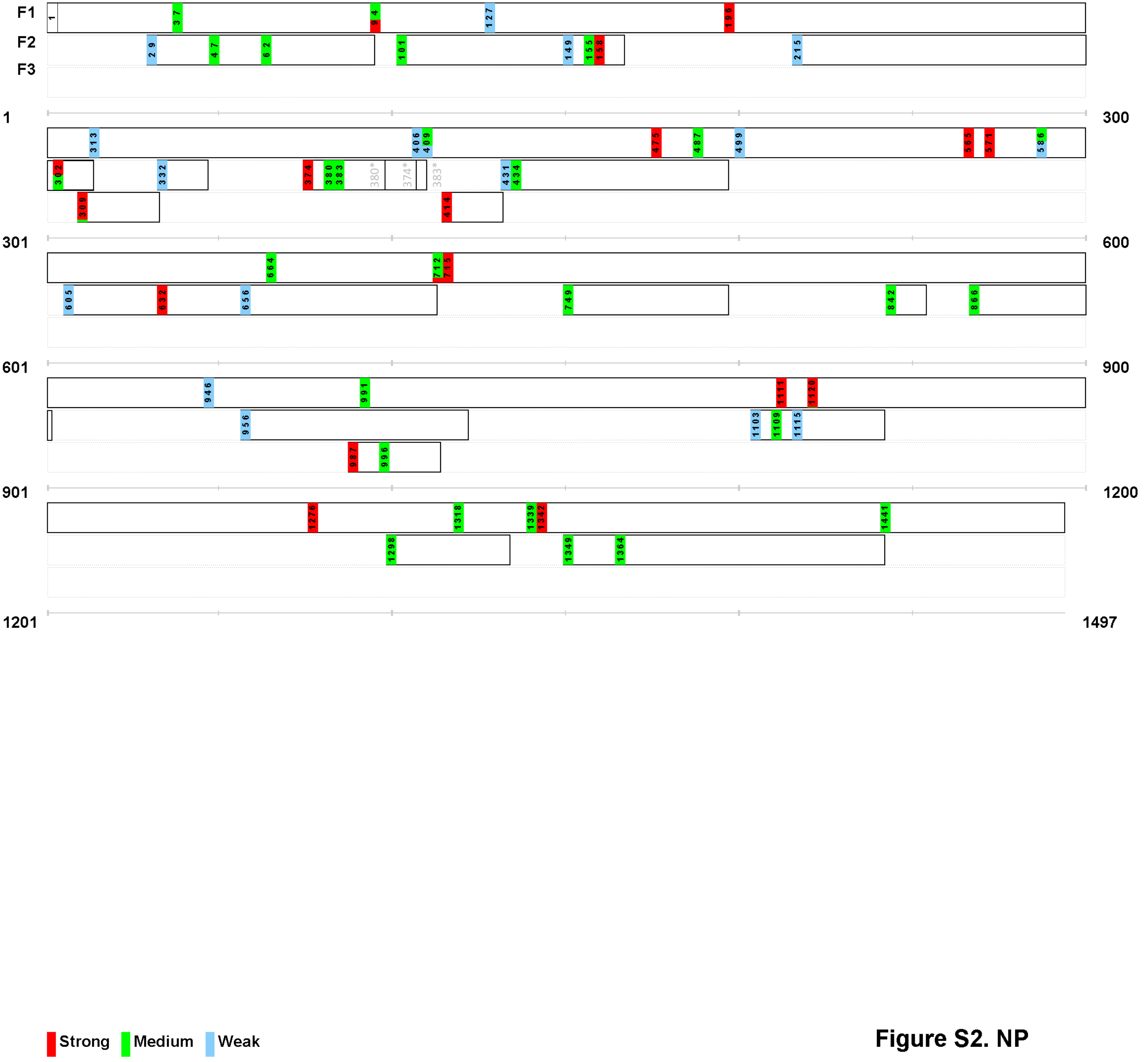
F1
F2
F3
1
300
301
600
601
900
901
1200
1201
1497
Strong
Medium
Weak

Figure S2. NP

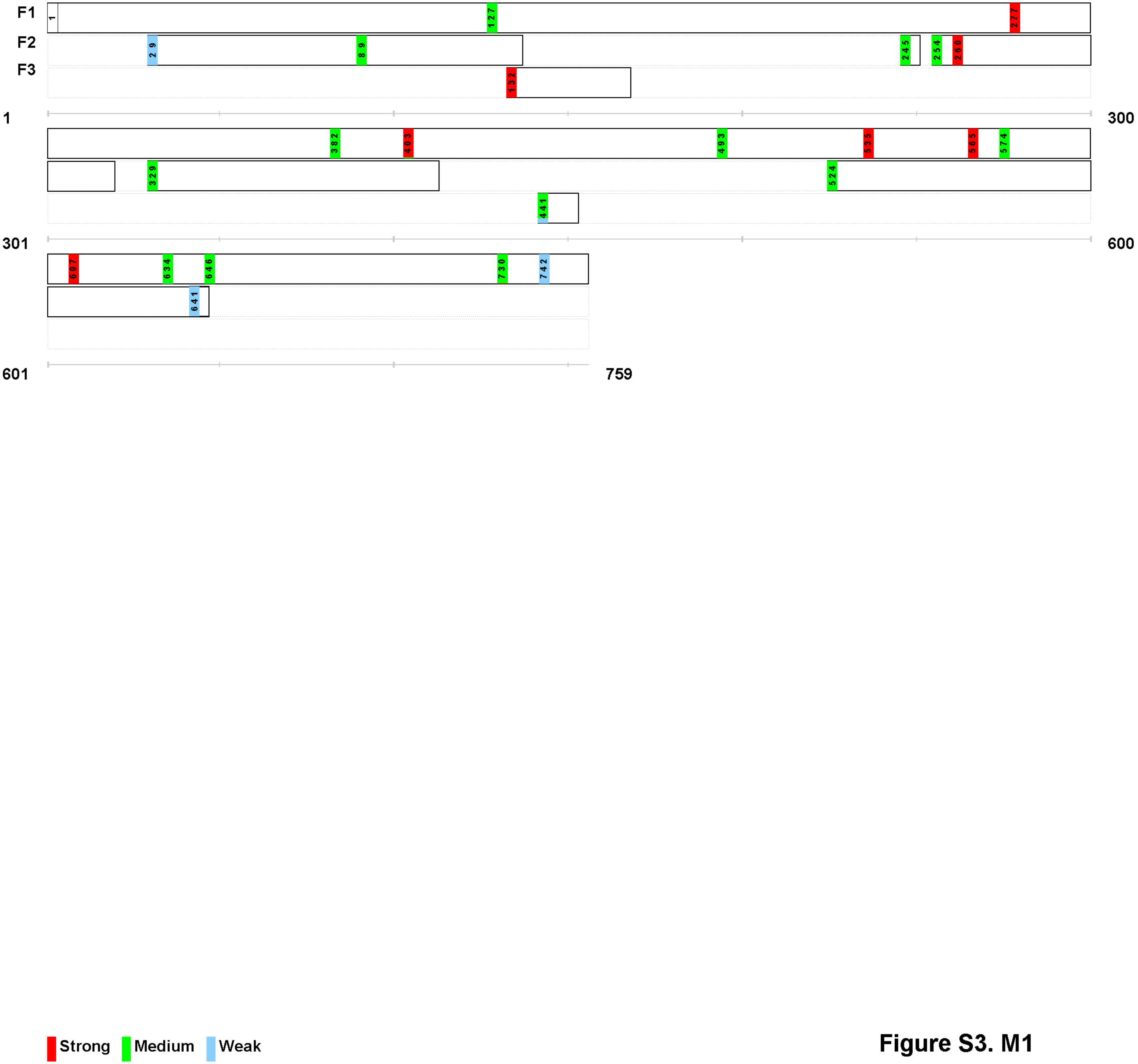

Figure S3. M1

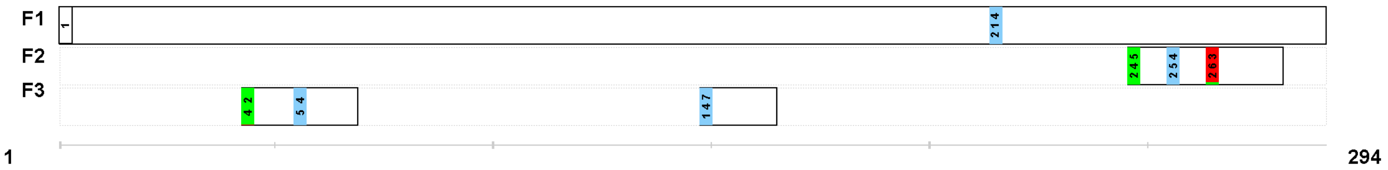

Strong Medium Weak

Figure S4. M2

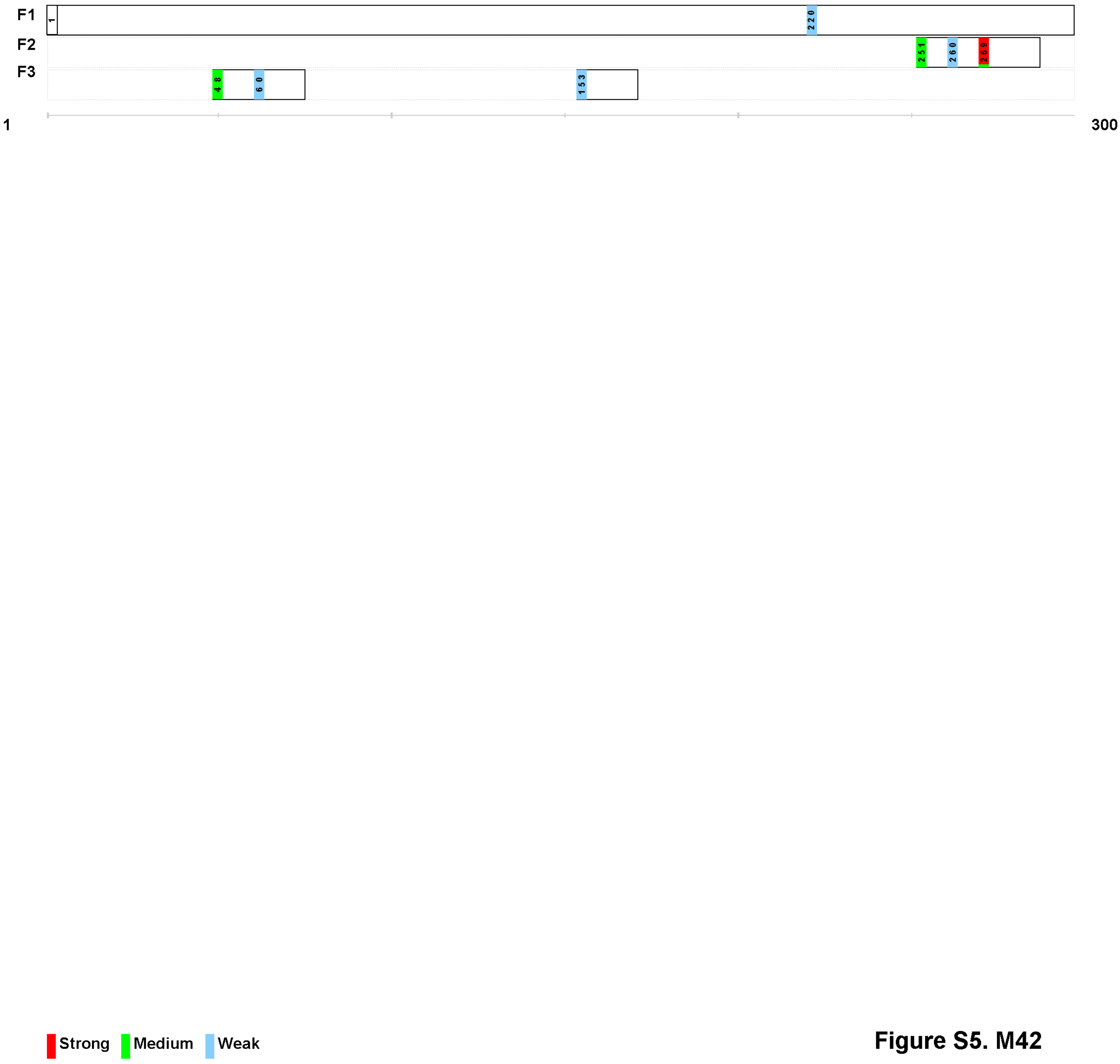

Figure S5. M42

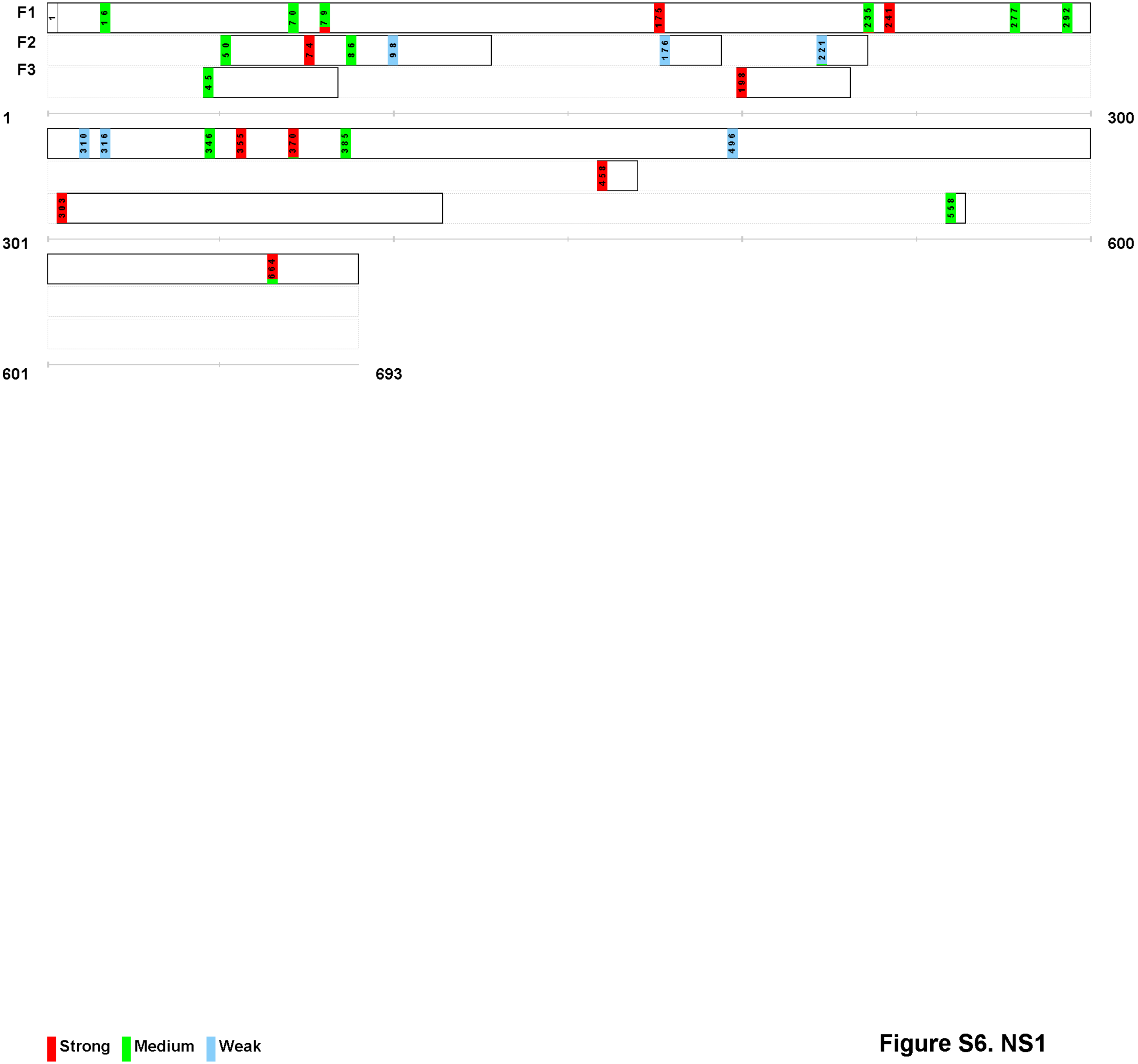

Figure S6. NS1

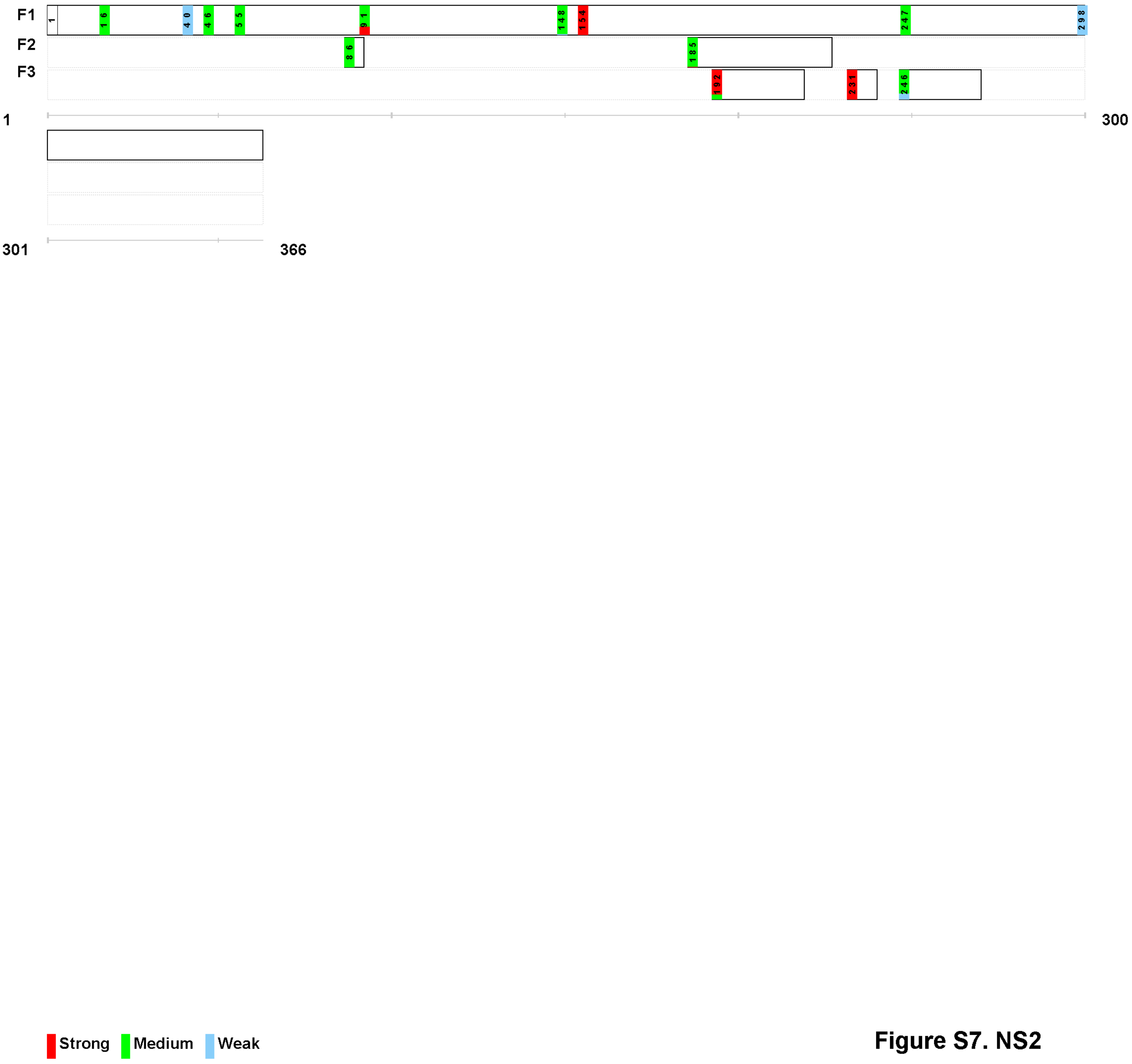

Figure S7. NS2

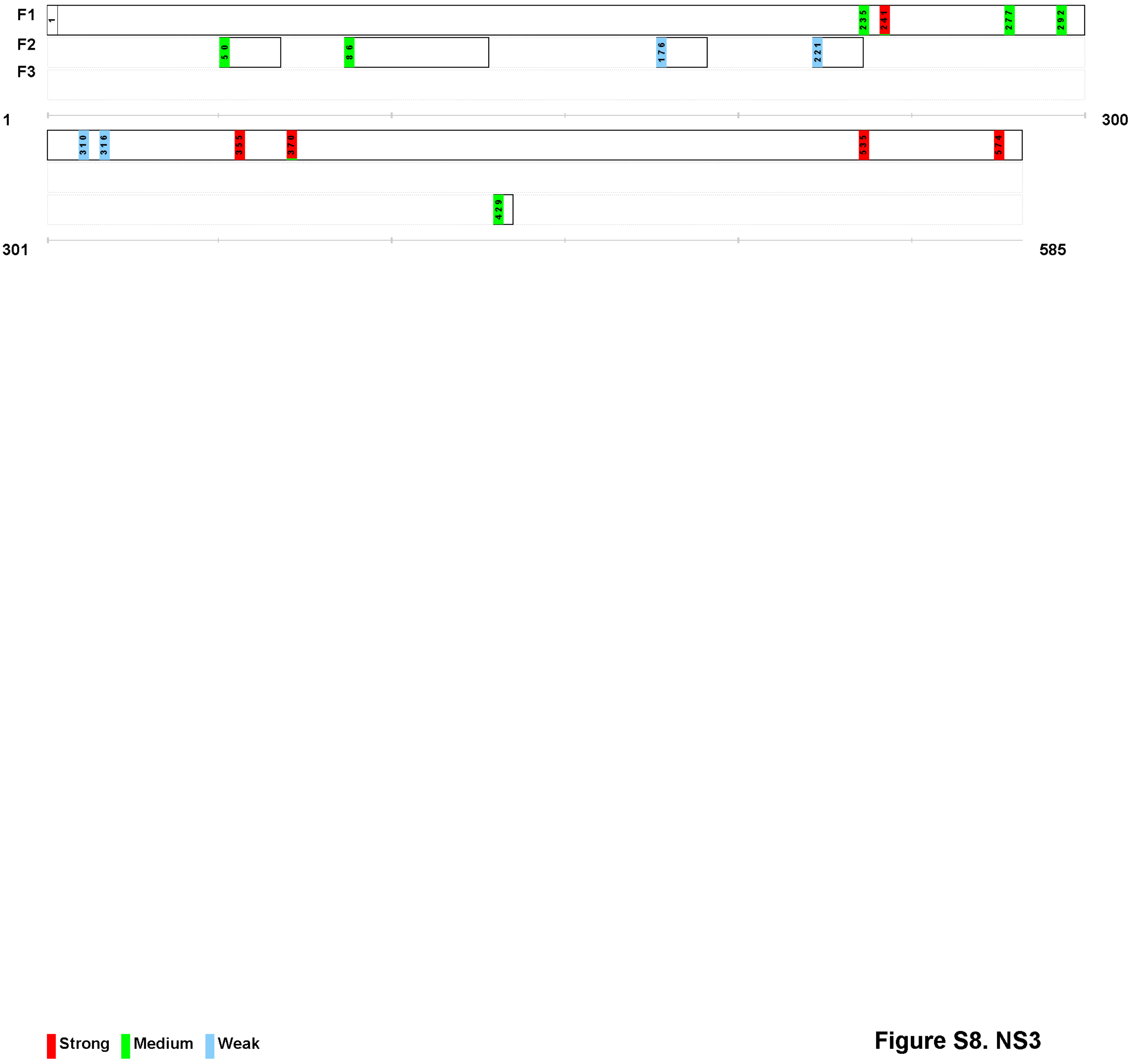

Figure S8. NS3

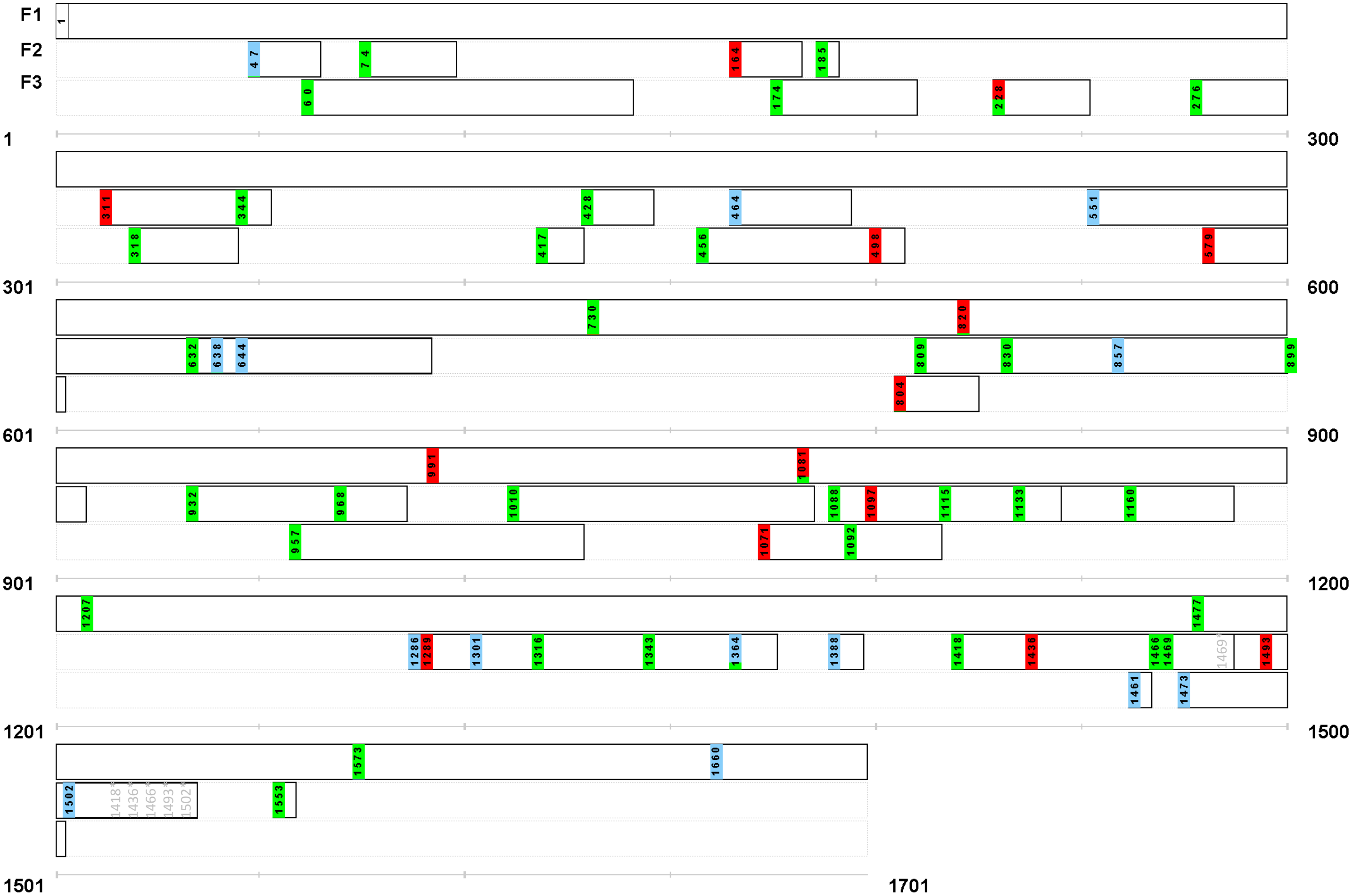

Strong Medium Weak

Figure S9. H1

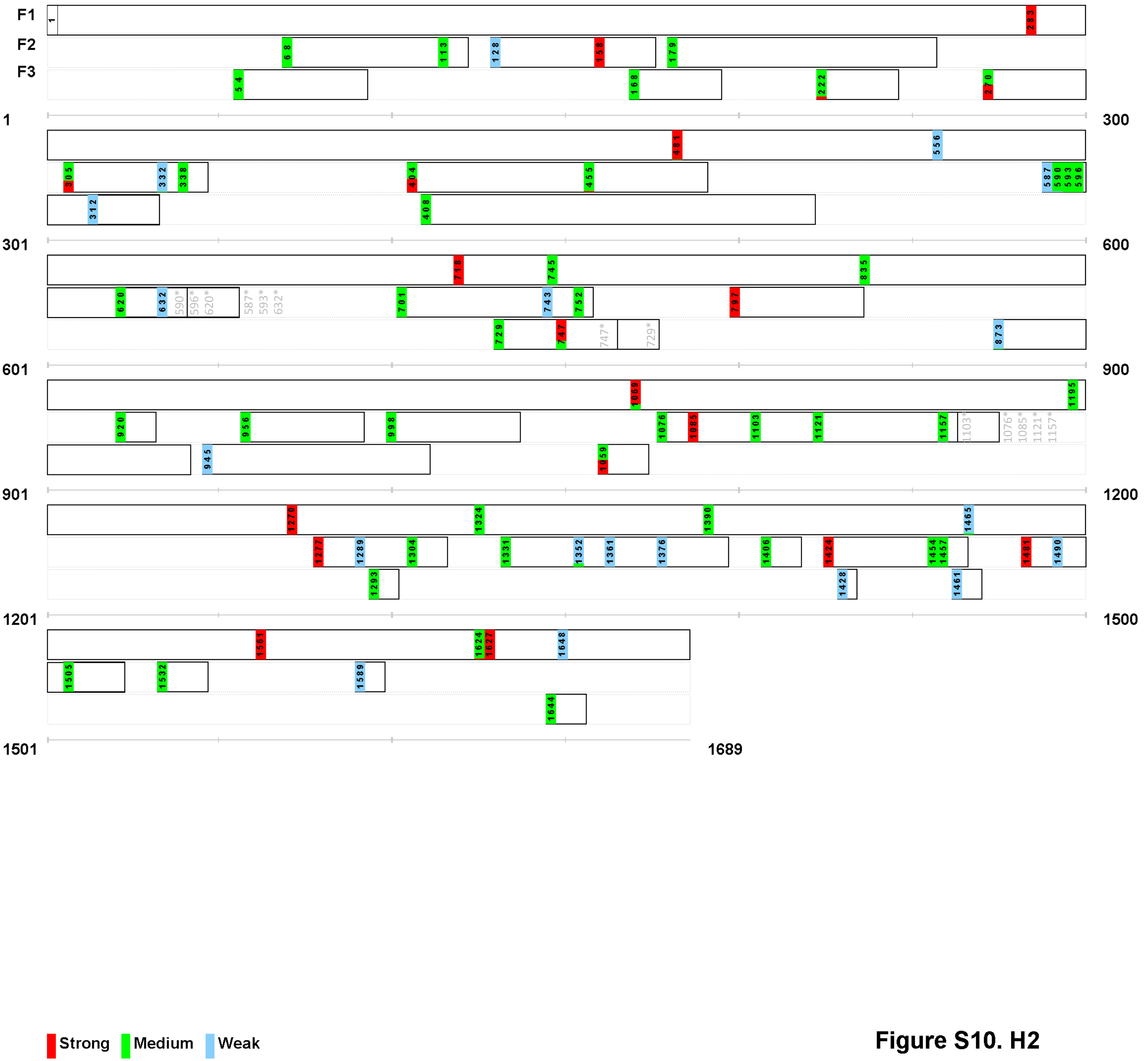

Figure S10. H2

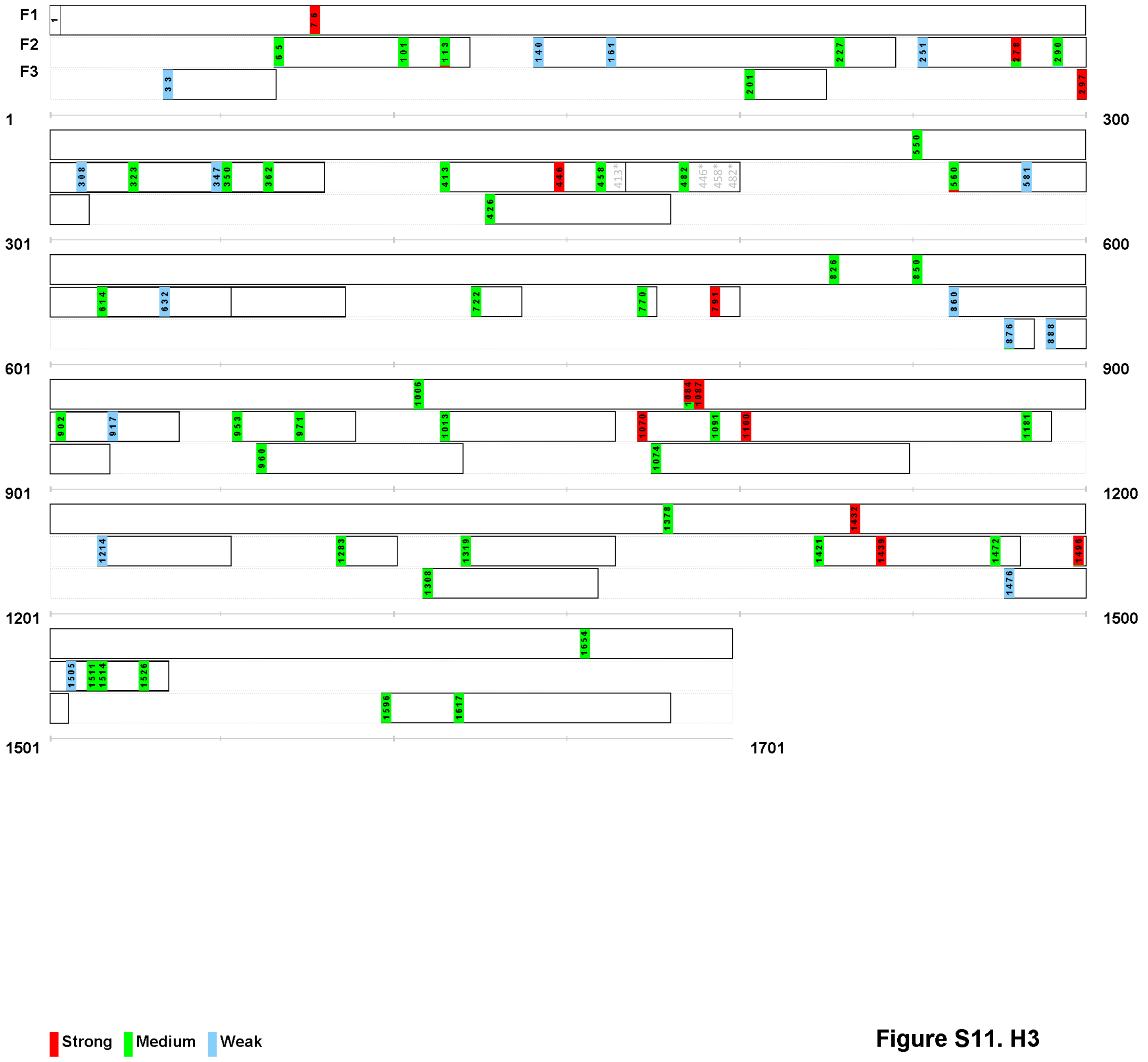

Figure S11. H3

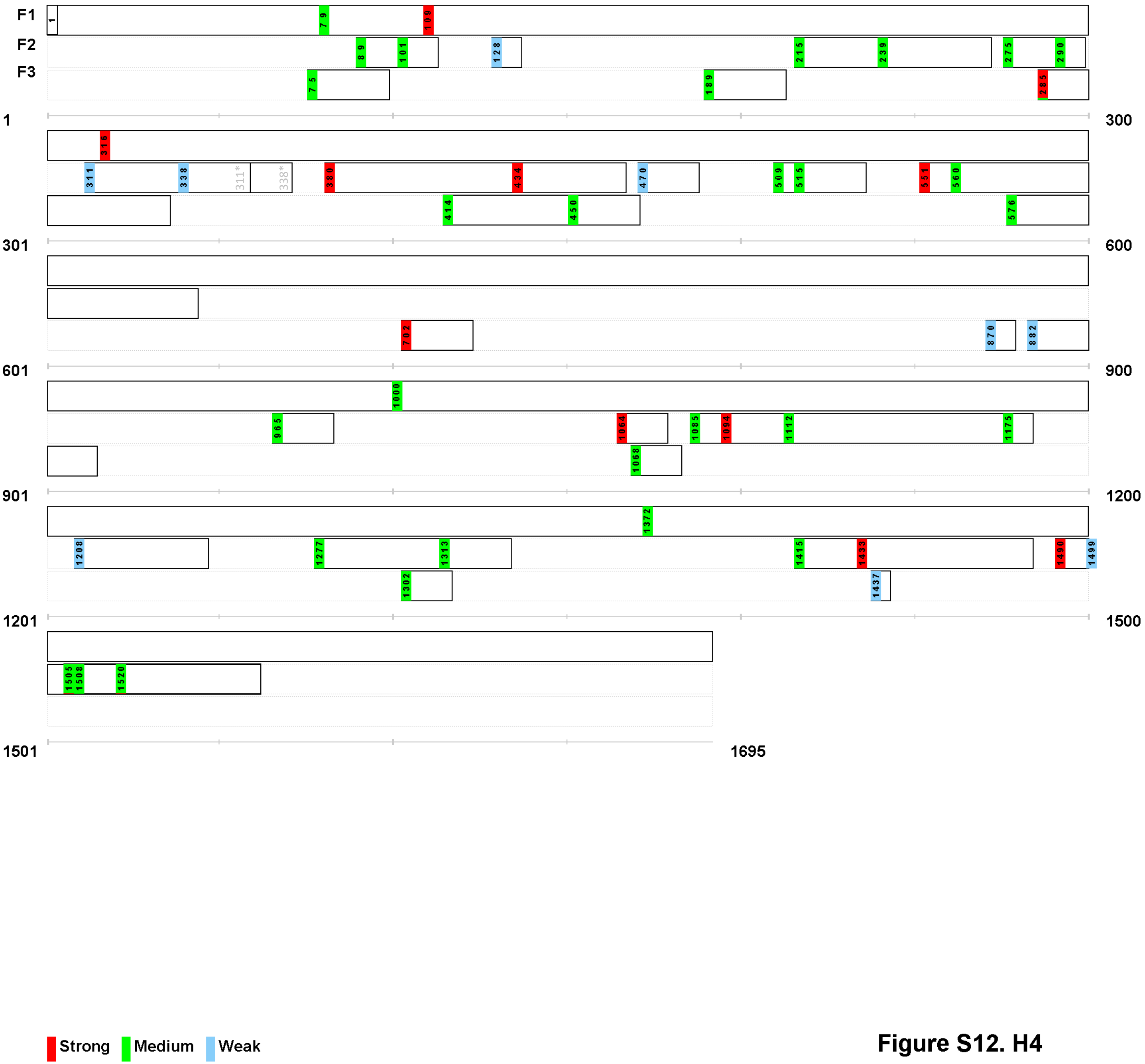

Figure S12. H4

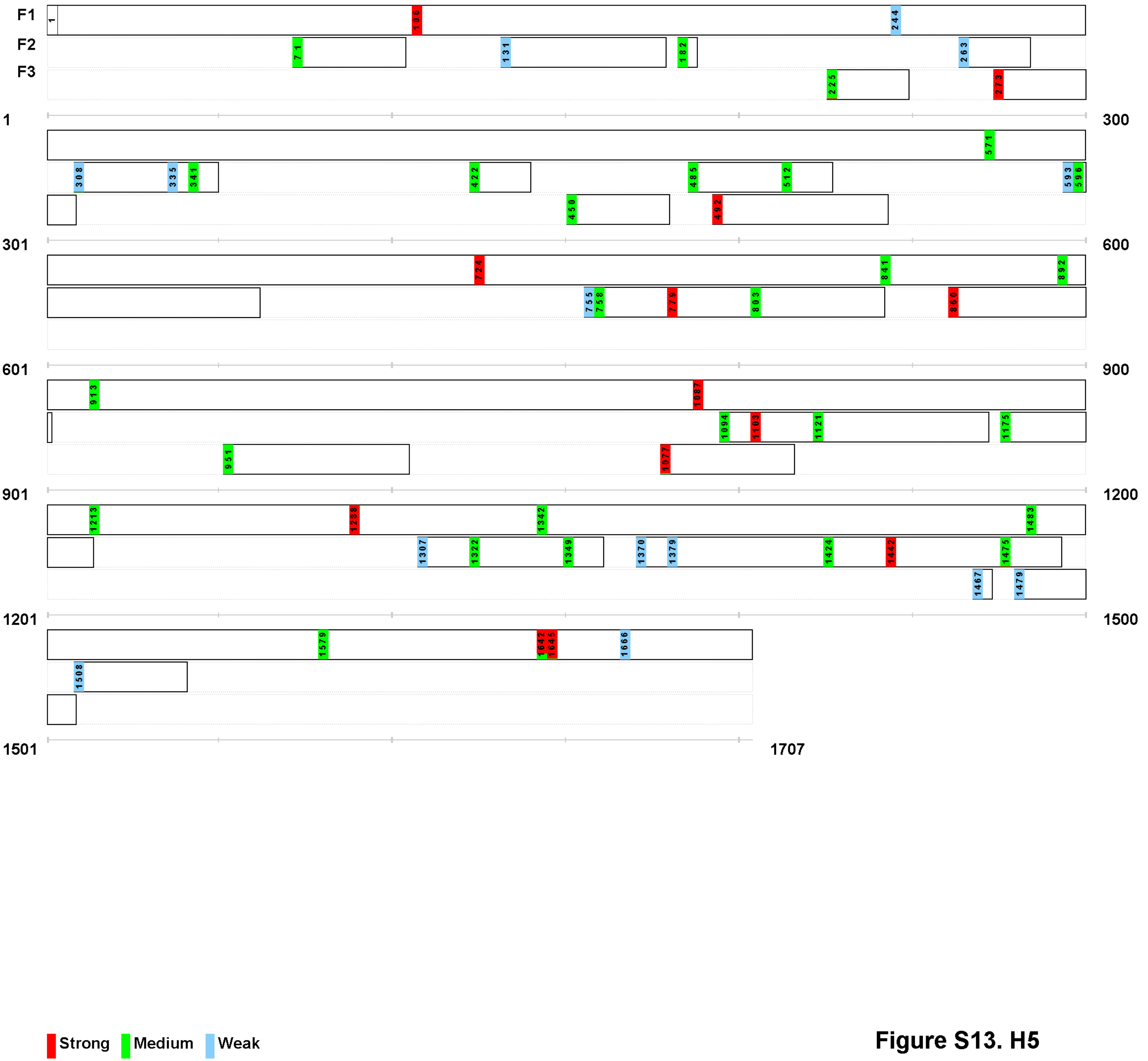

Figure S13. H5

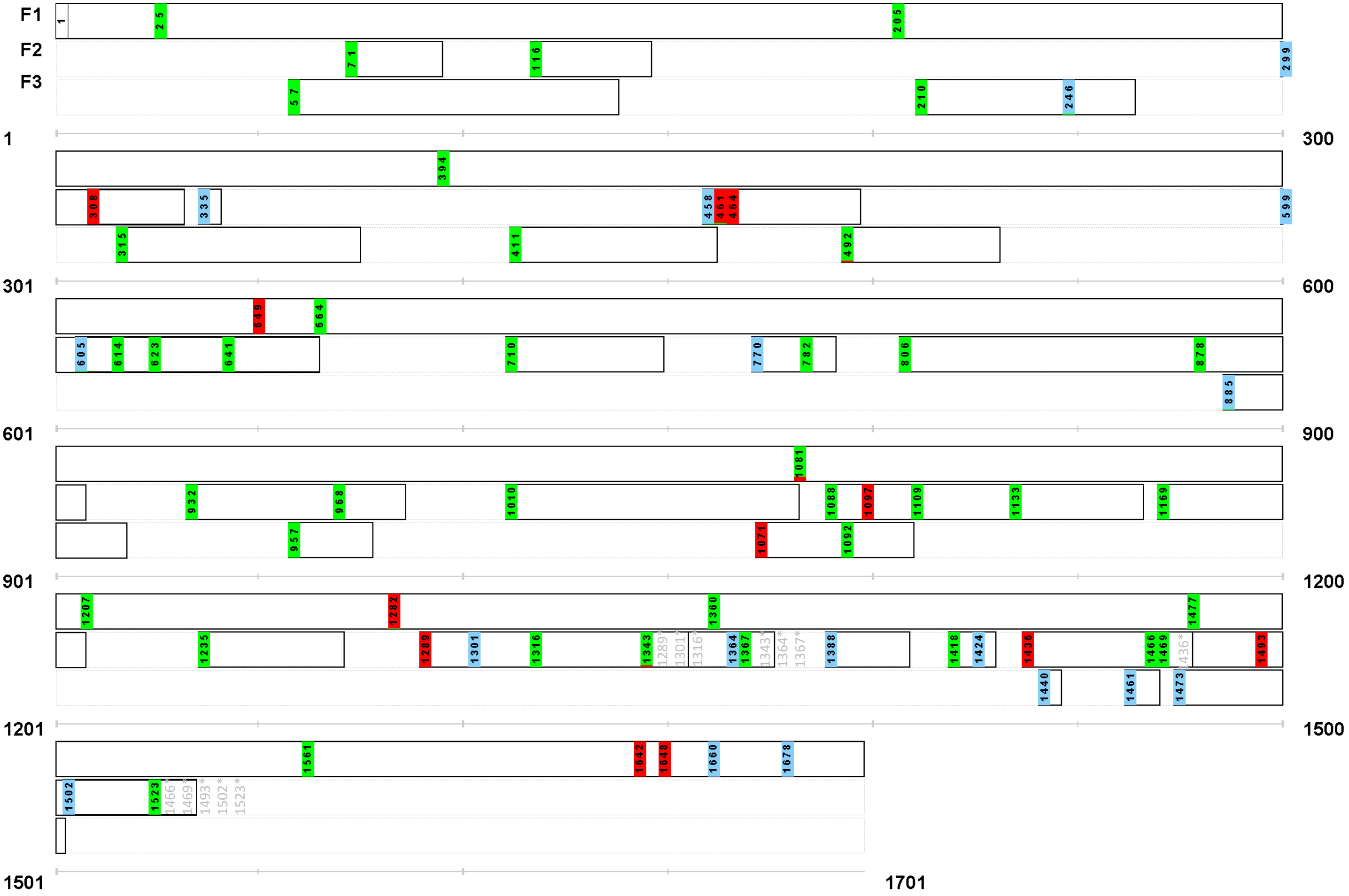

Strong Medium Weak

Figure S14. H6

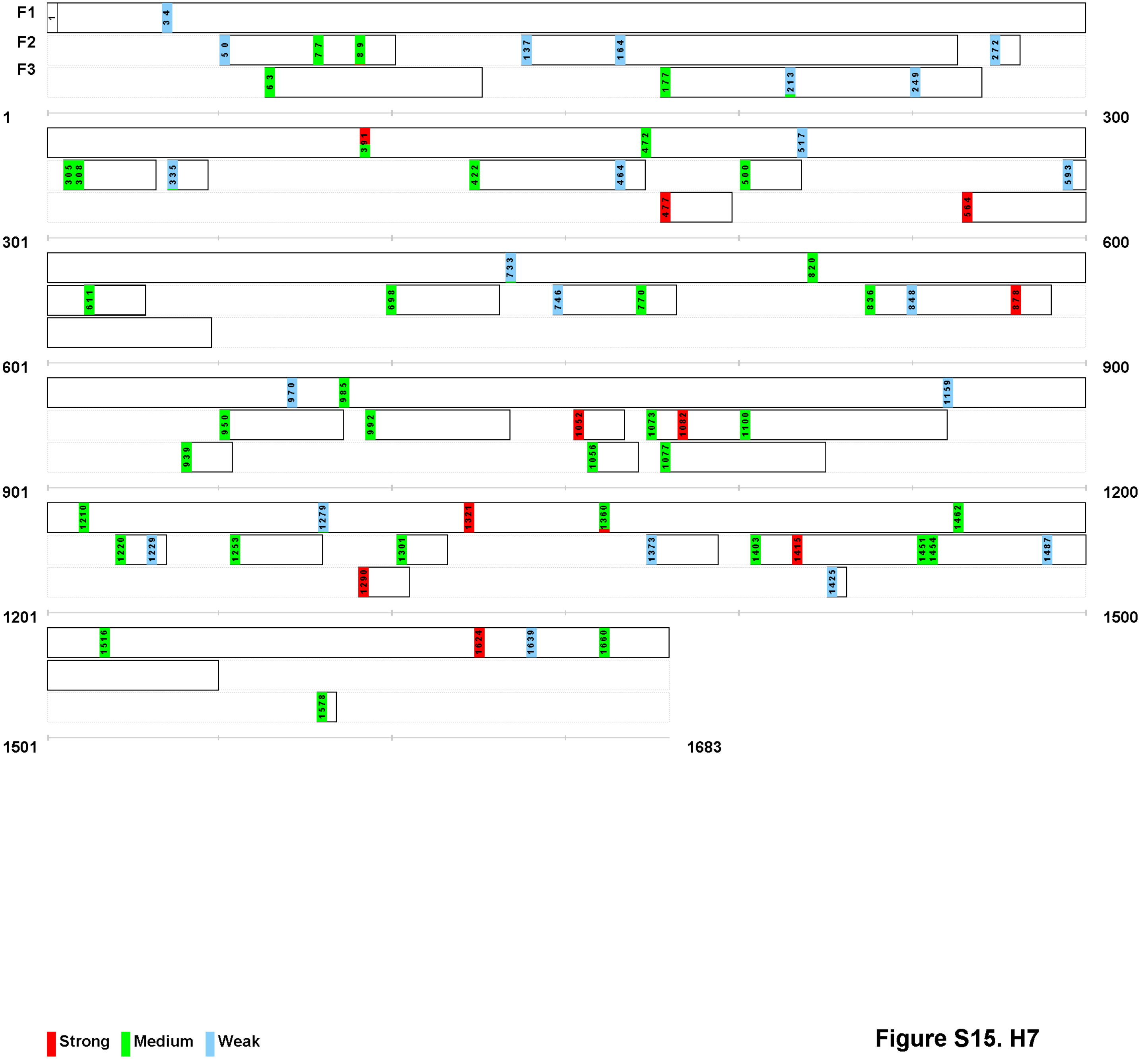

Figure S15. H7

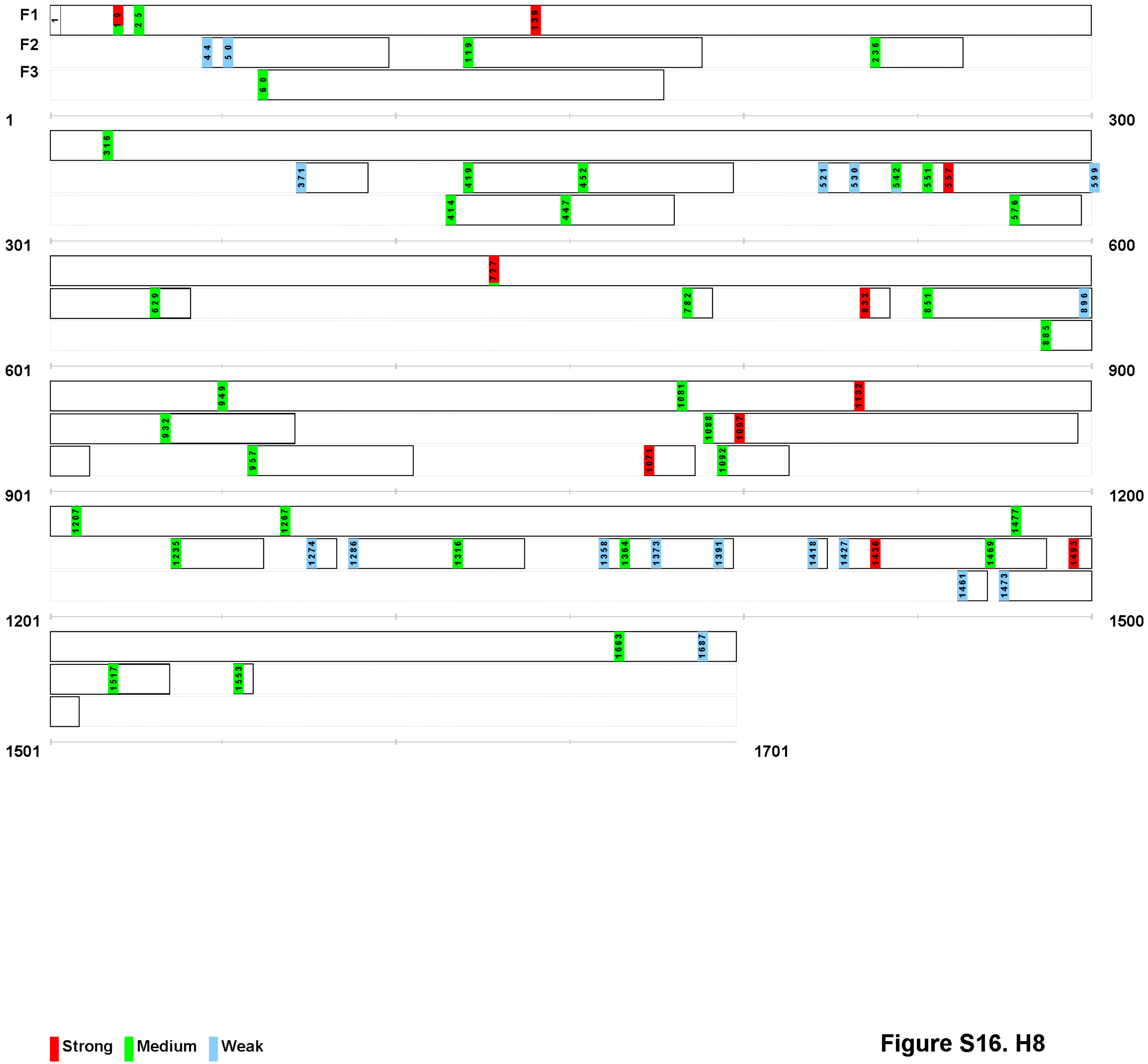

Figure S16. H8

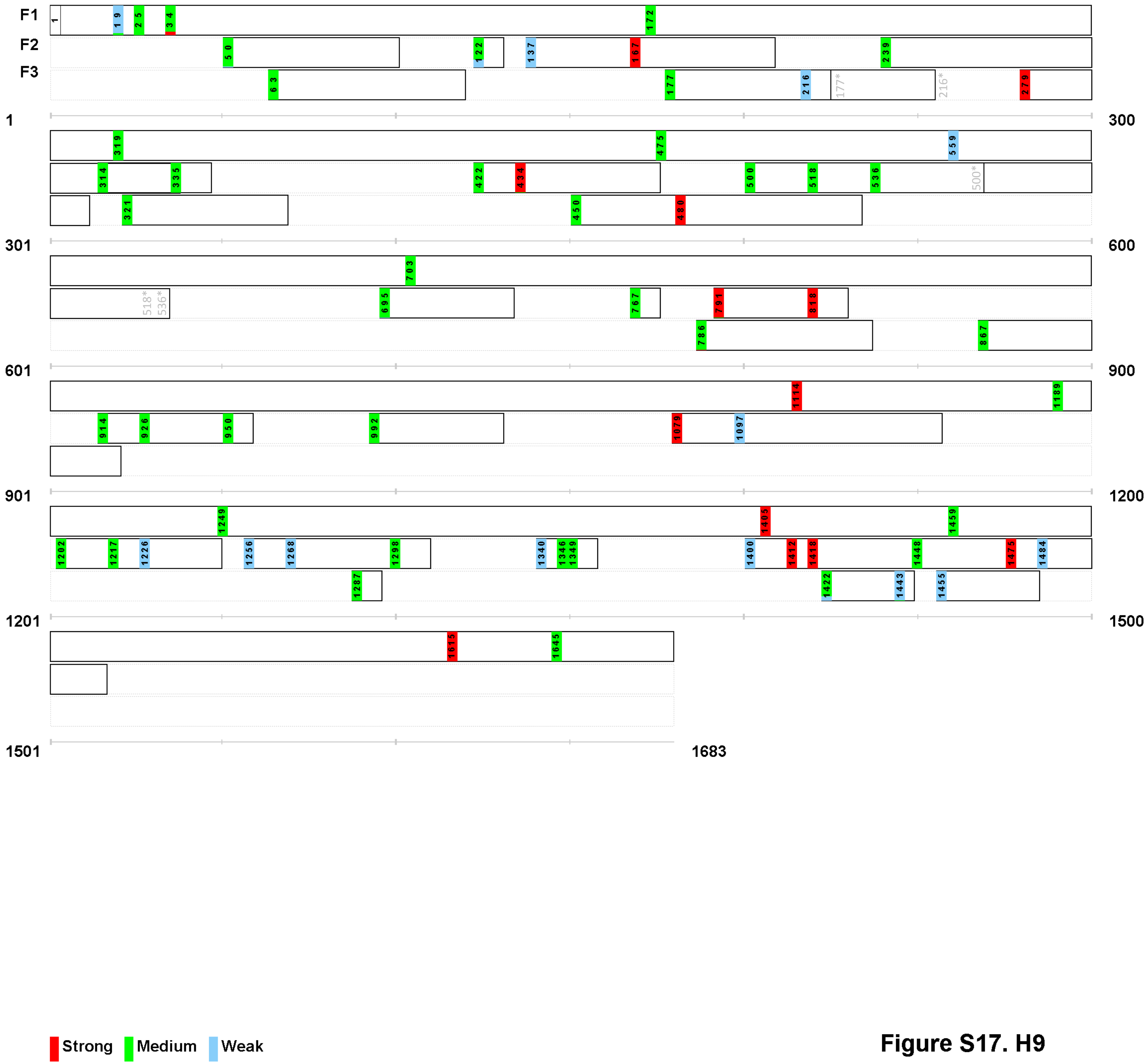

Figure S17. H9

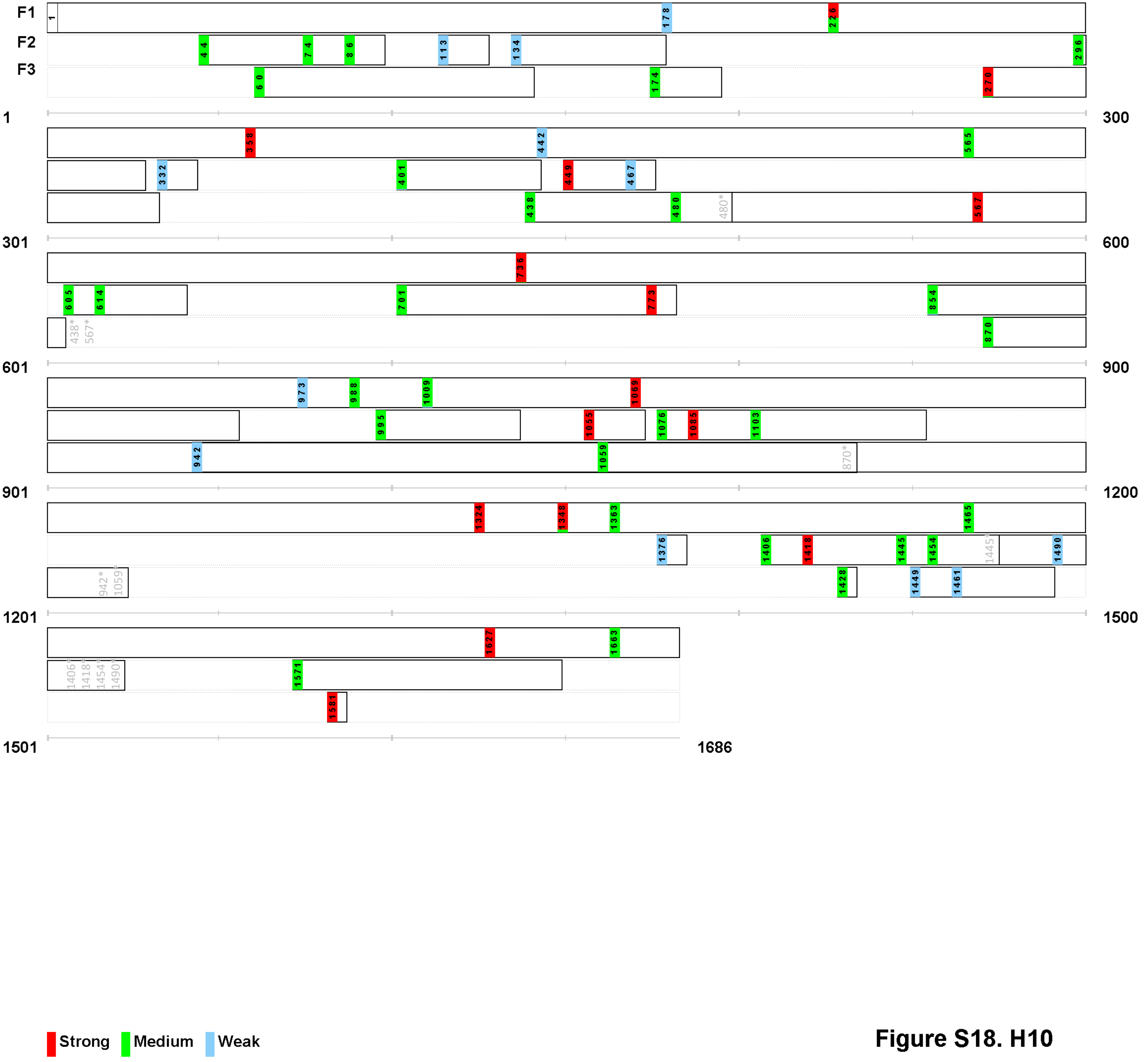

Figure S18. H10

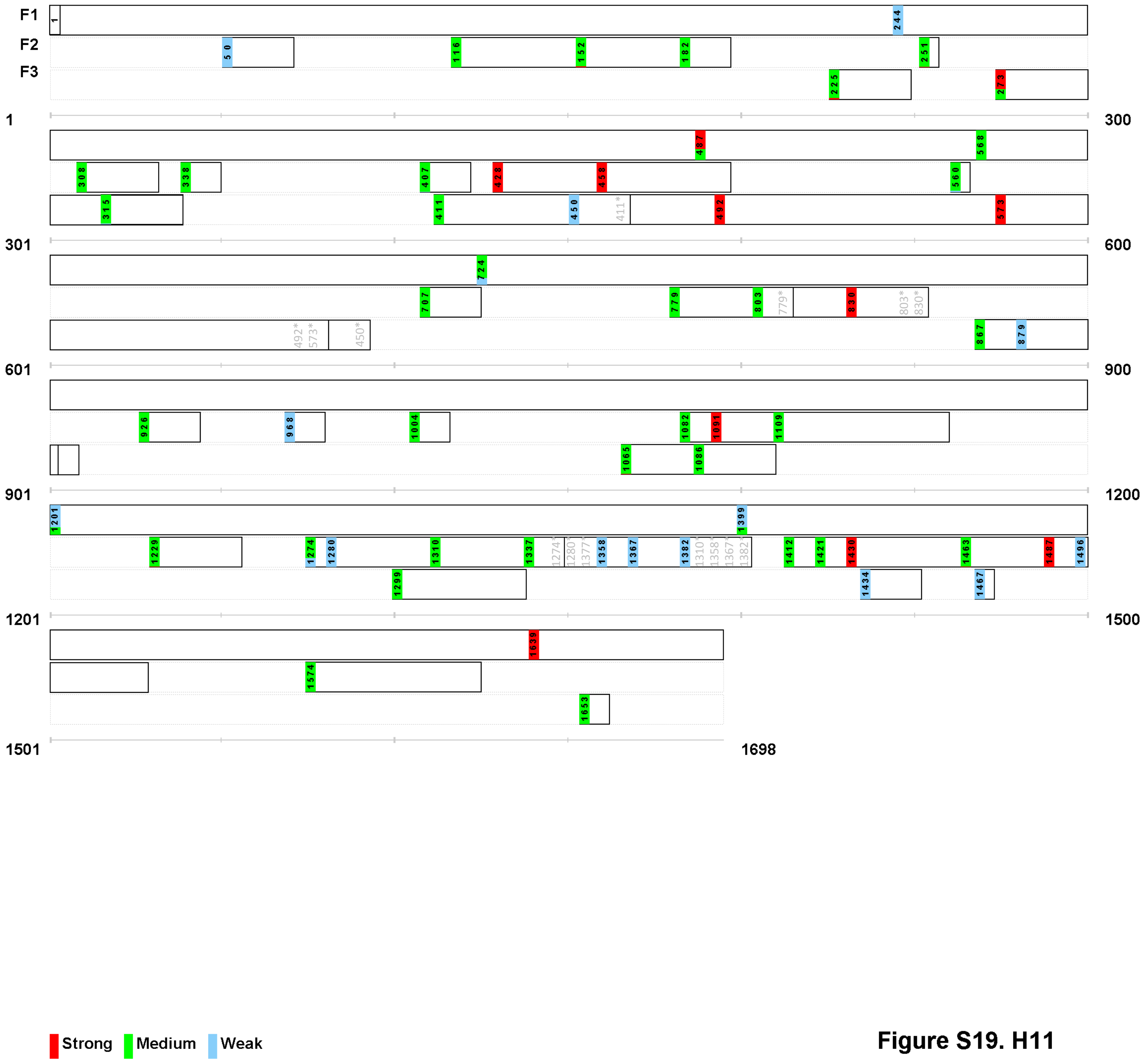

Figure S19. H11

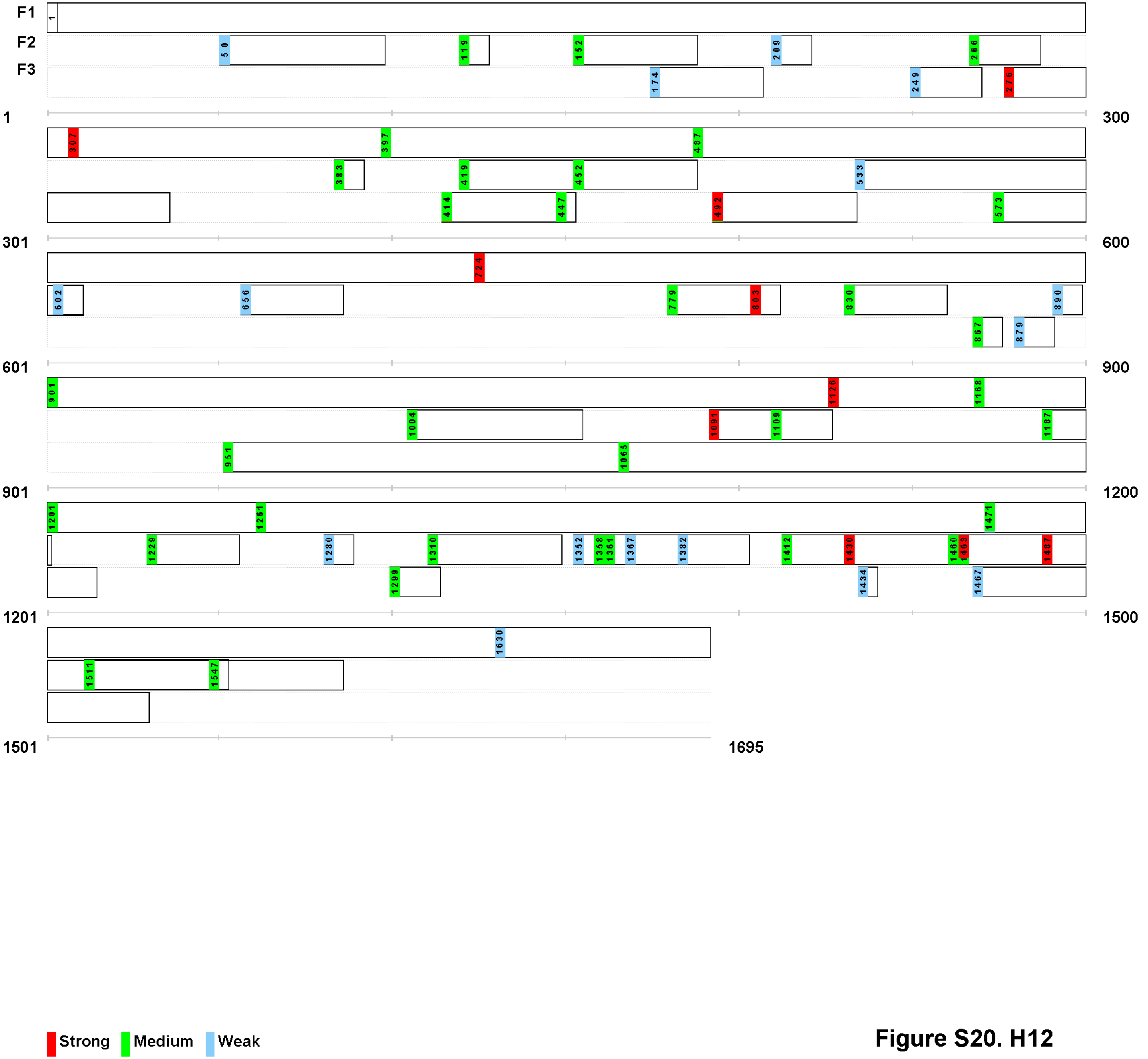

Figure S20. H12

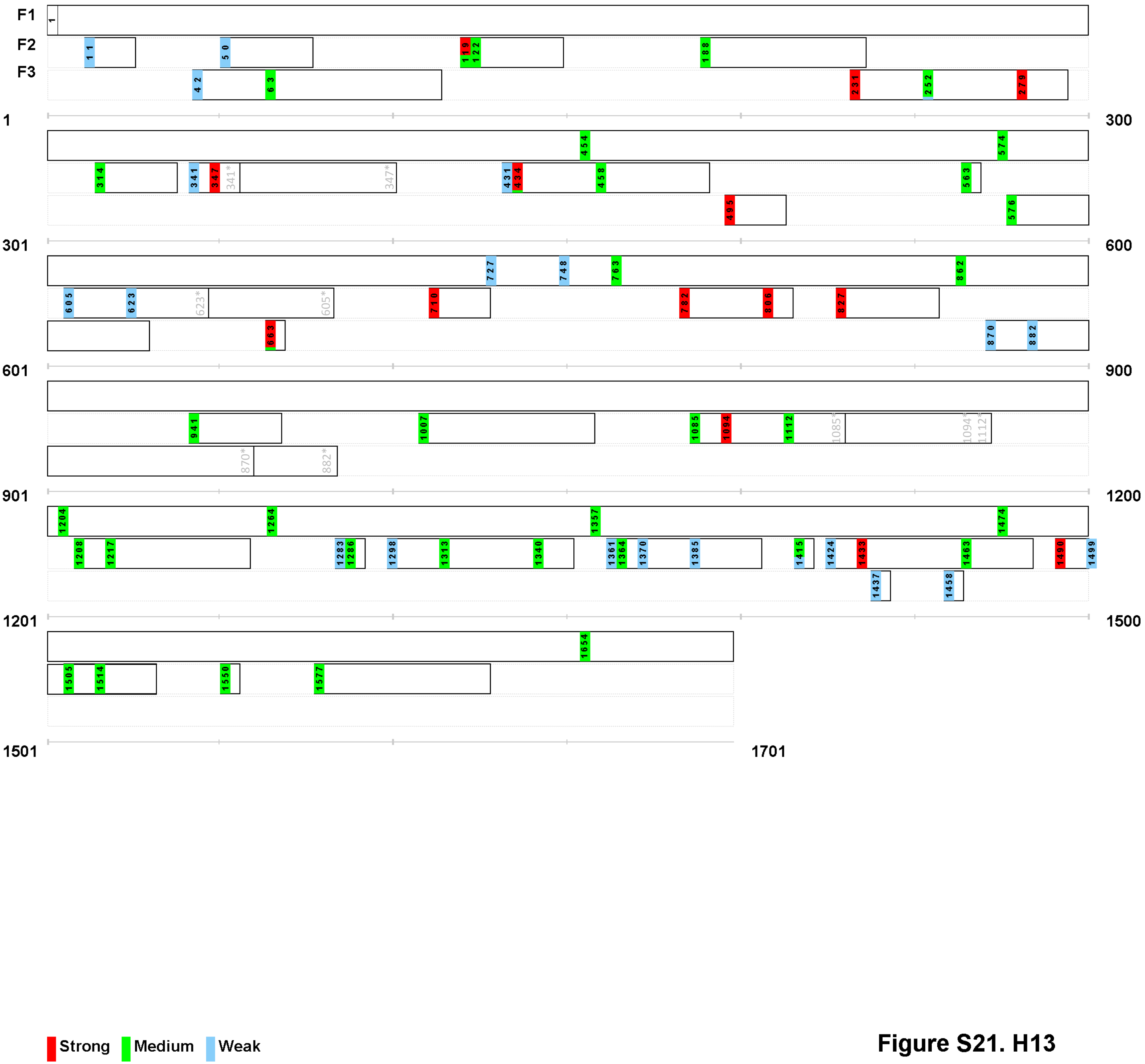

Figure S21. H13

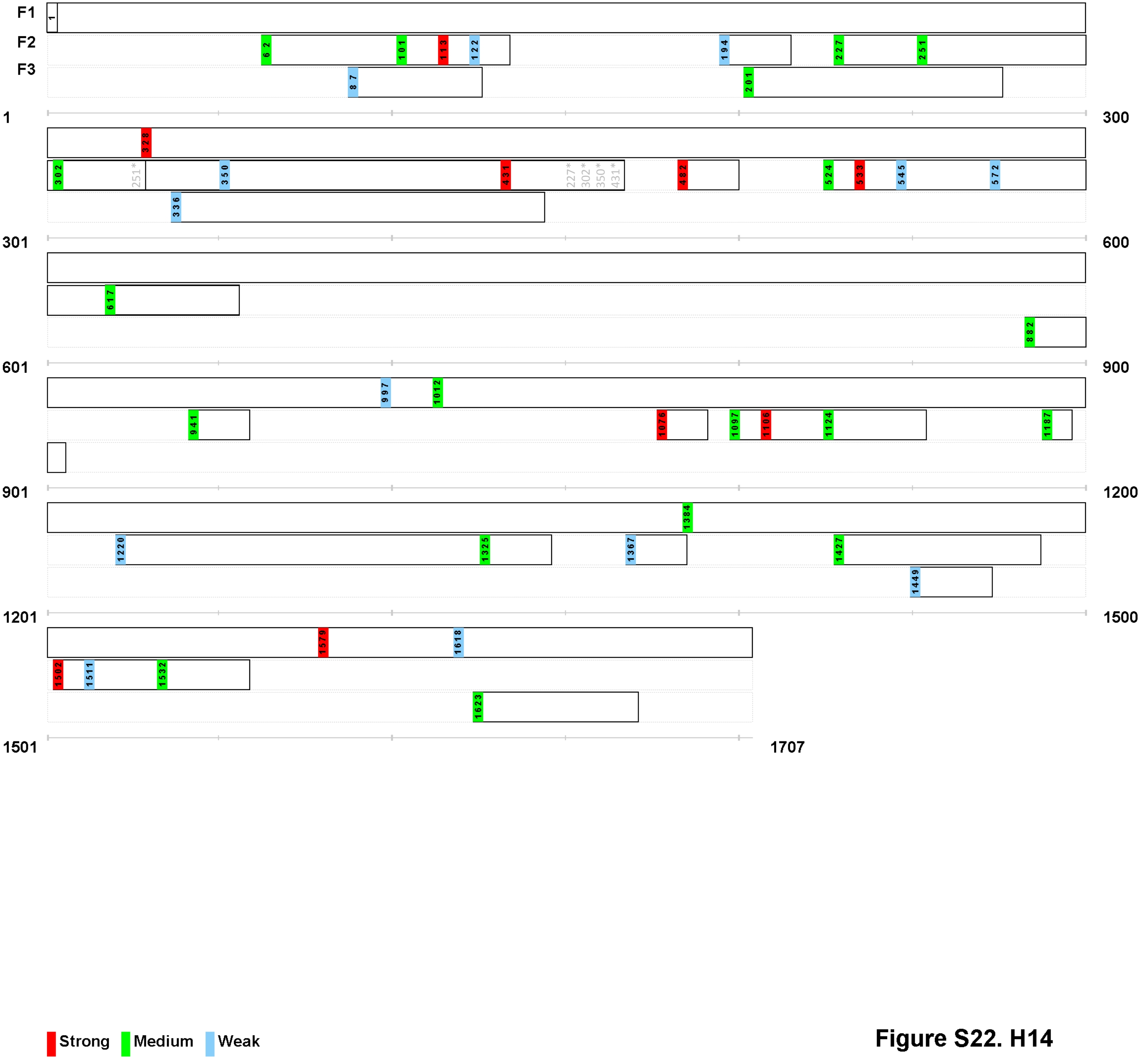

Figure S22. H14

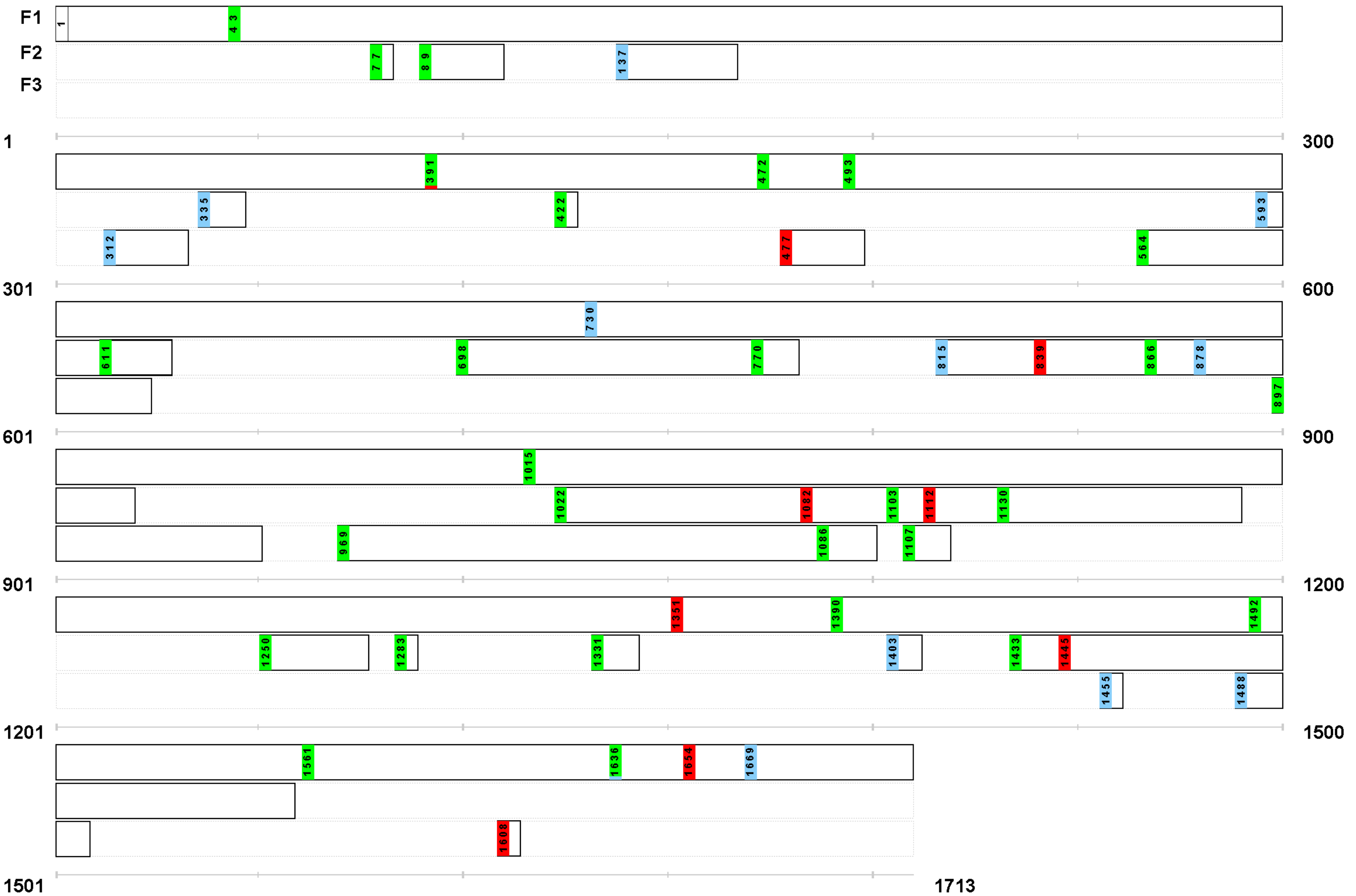

Strong Medium Weak

Figure S23. H15

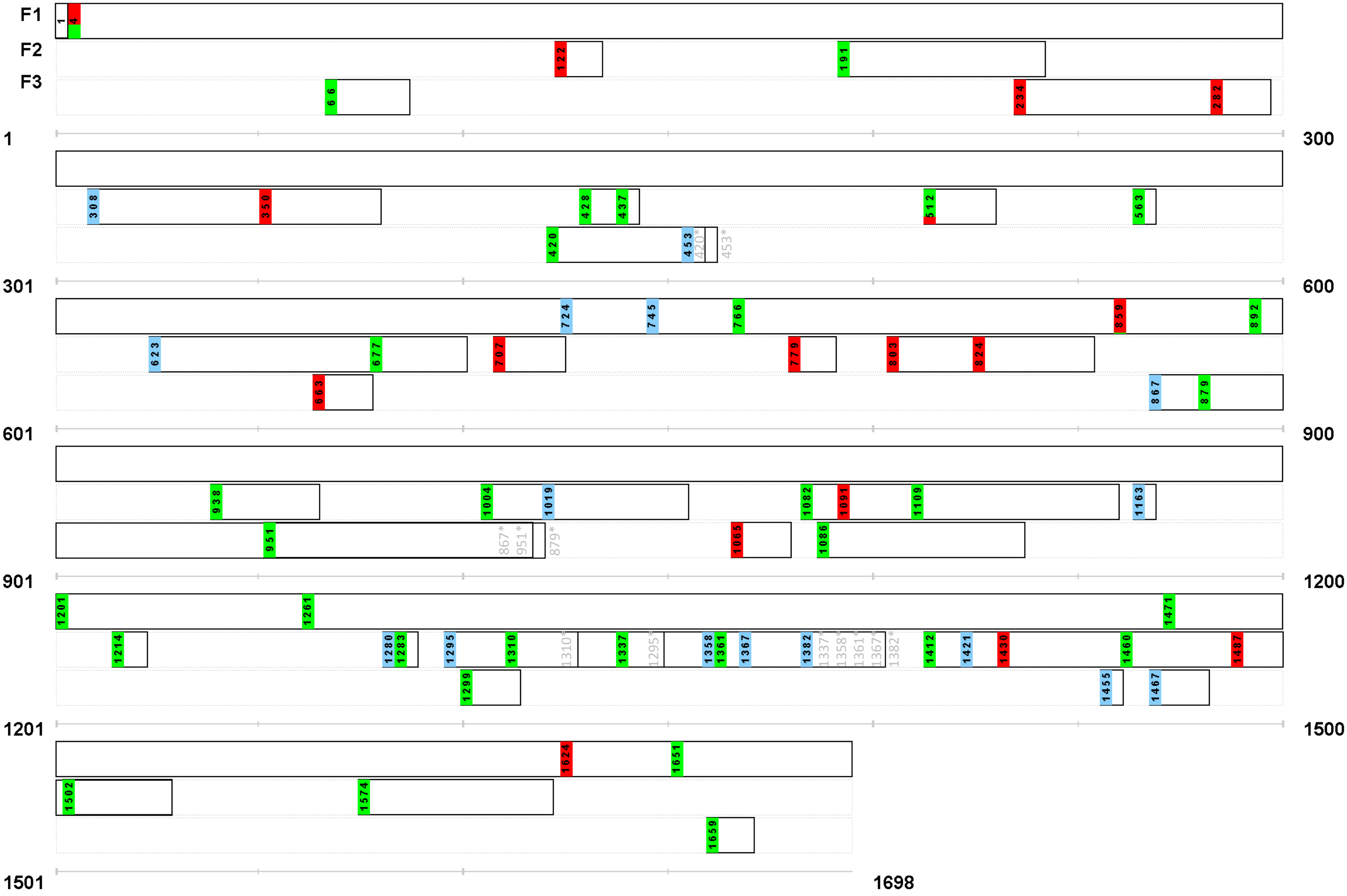

Strong Medium Weak

Figure S24. H16

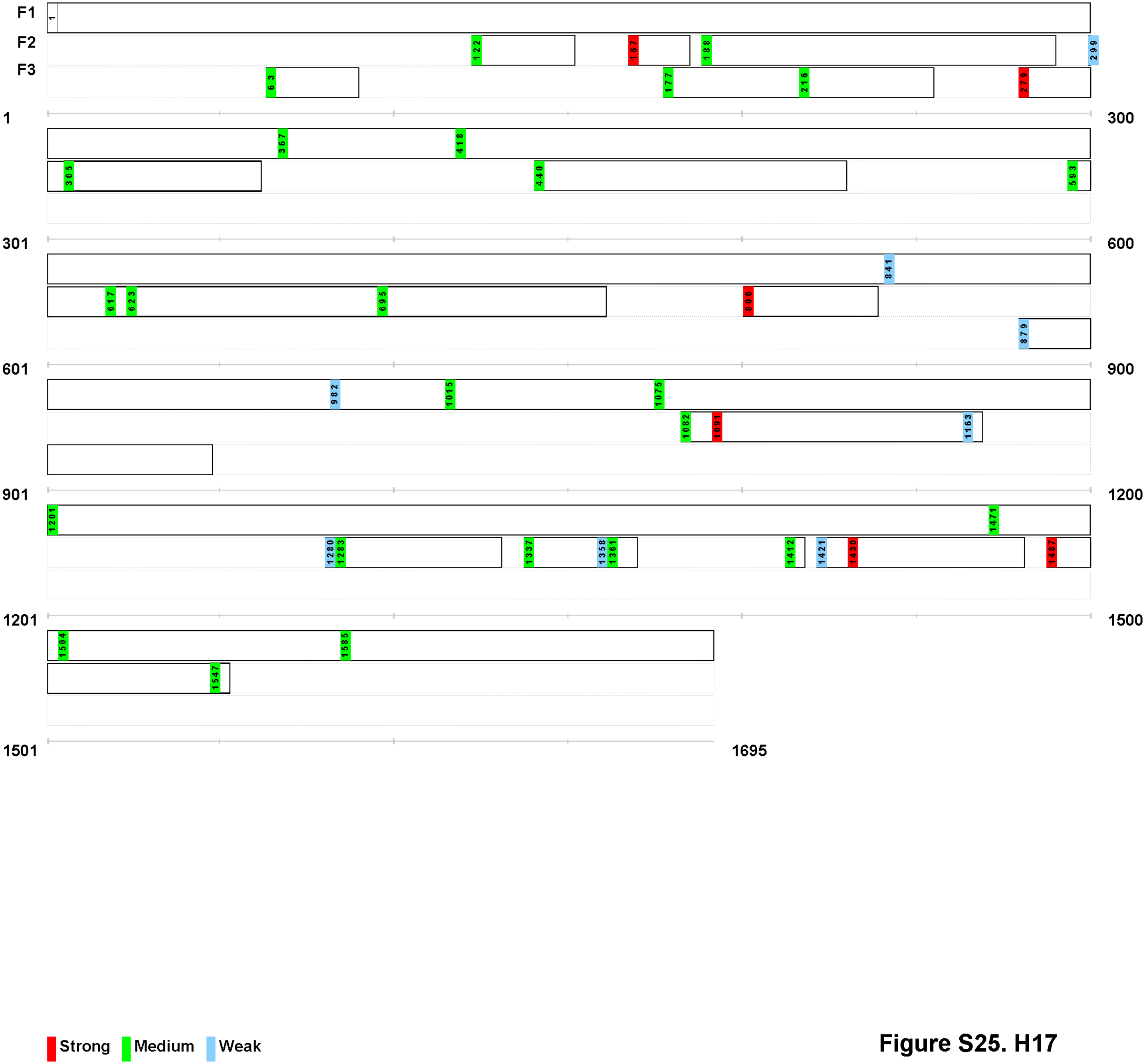

Figure S25. H17

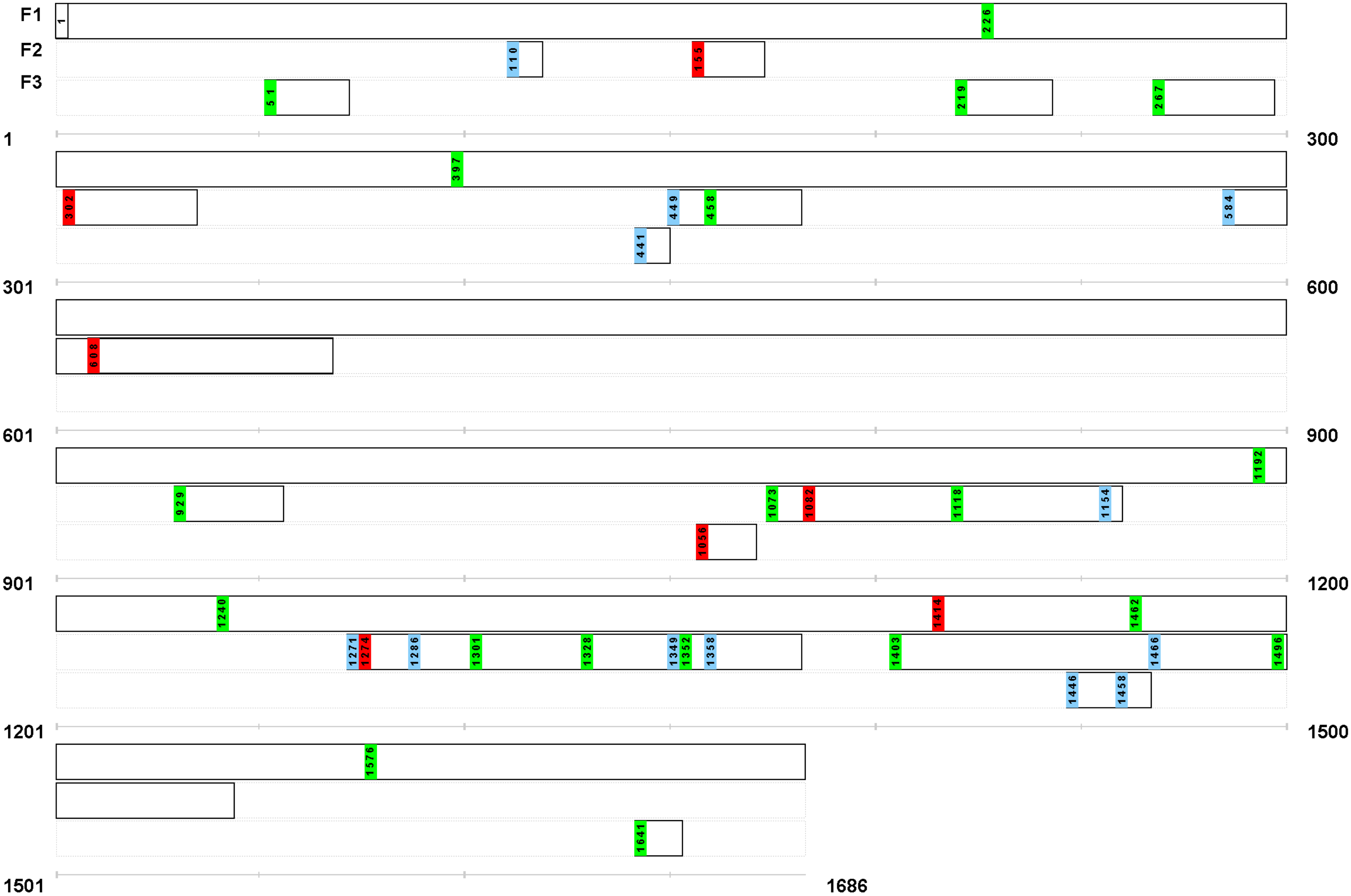

Figure S26. H18

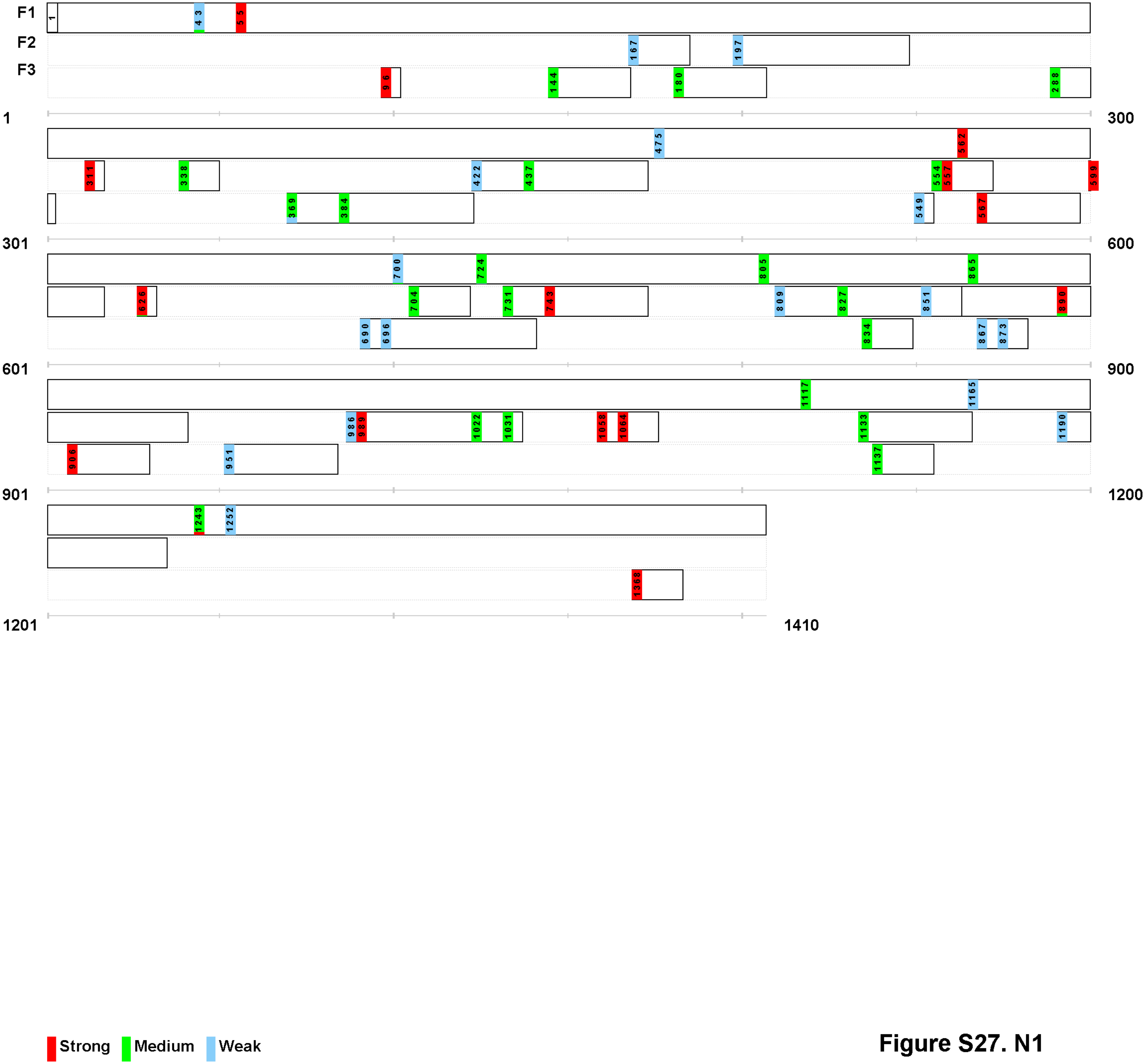

Figure S27. N1

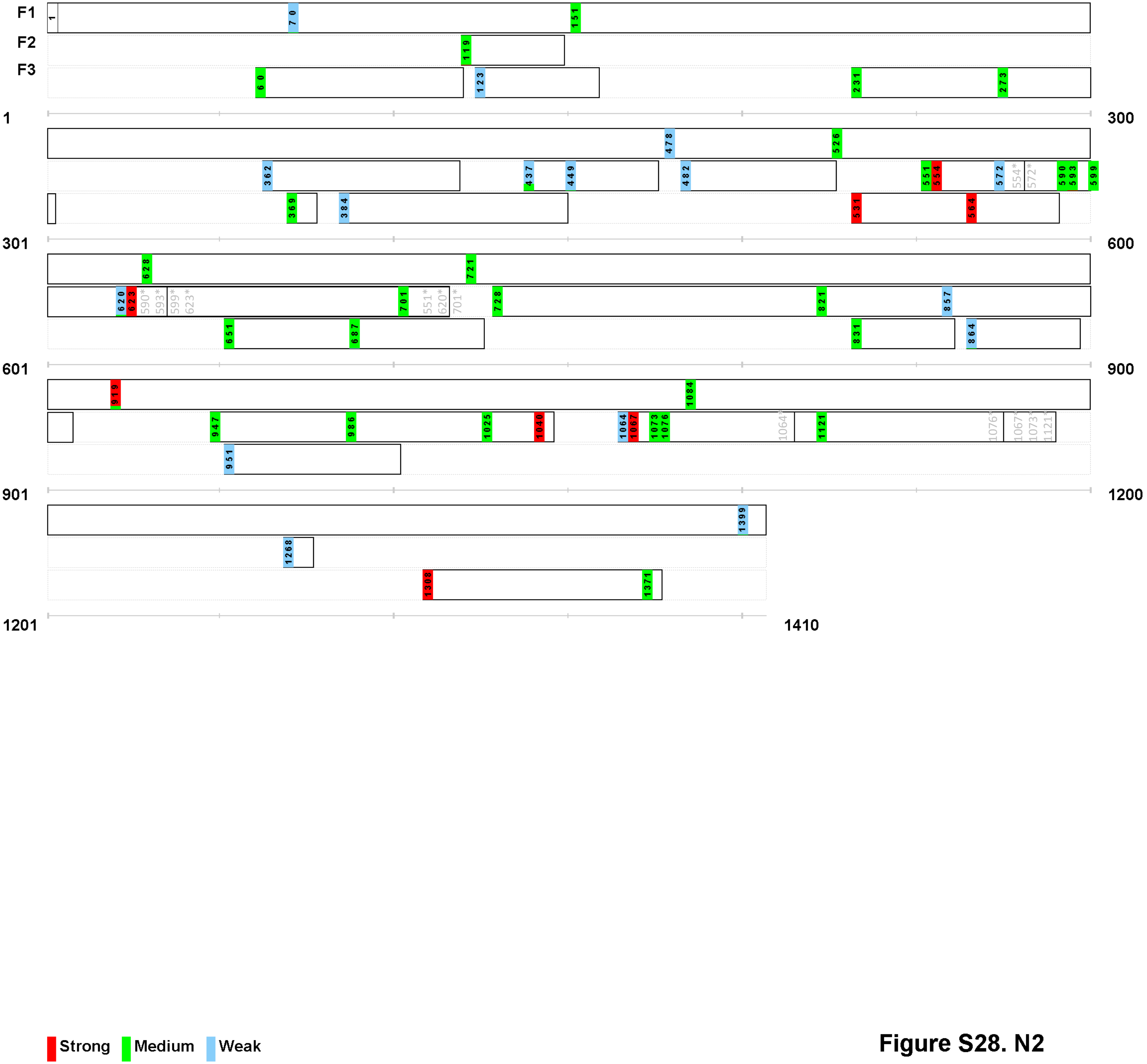

Figure S28. N2

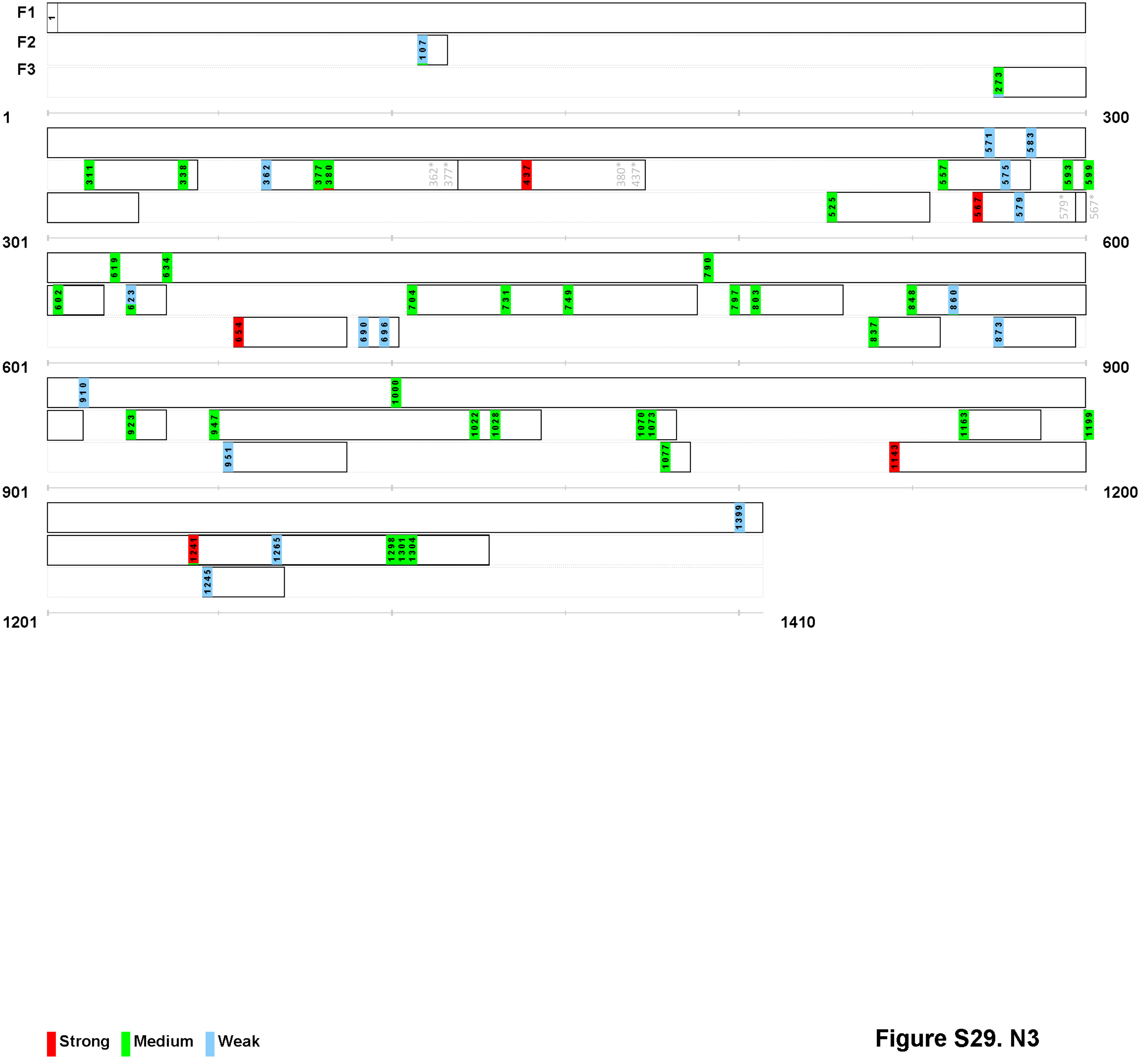

Figure S29. N3

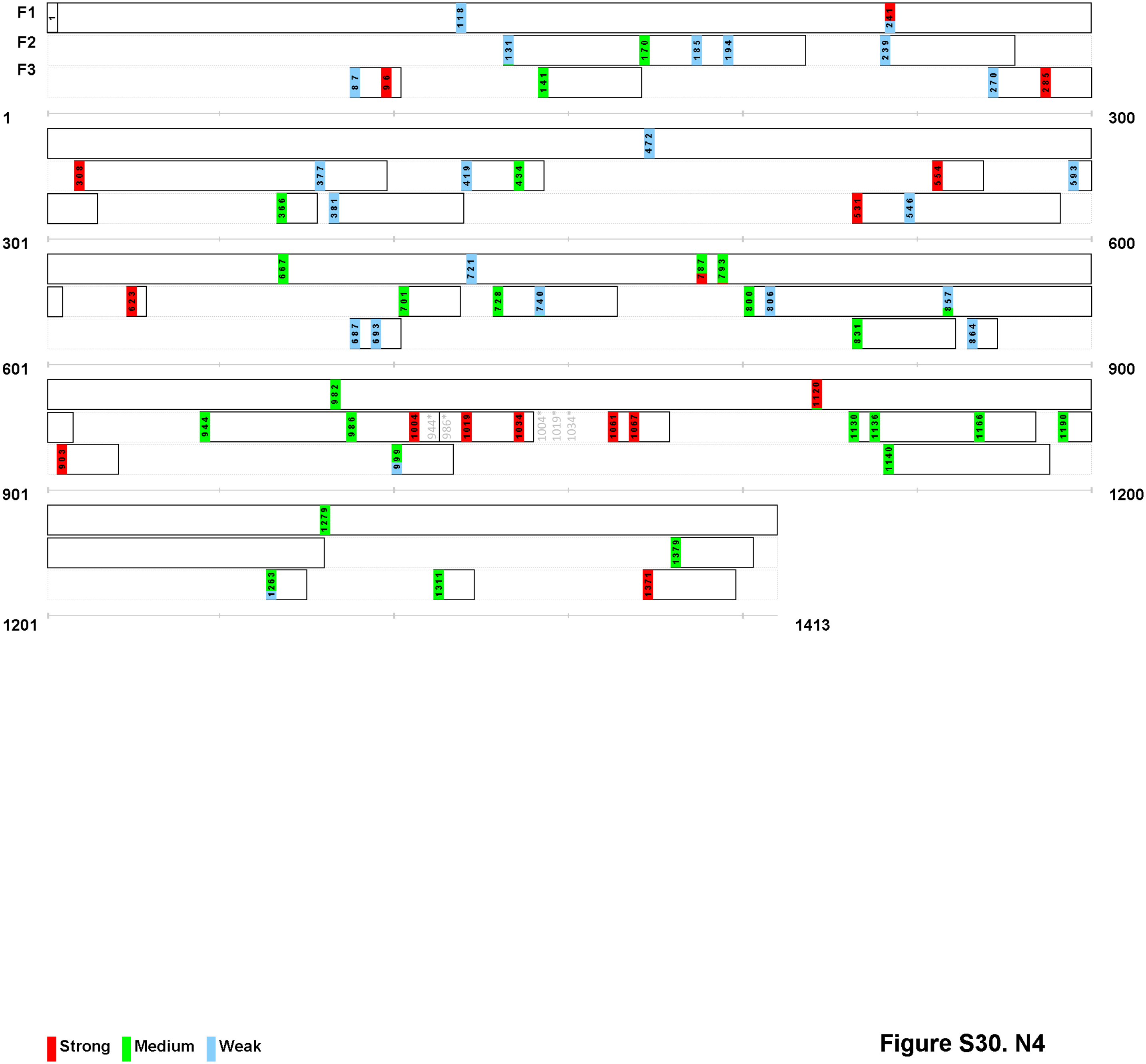

Figure S30. N4

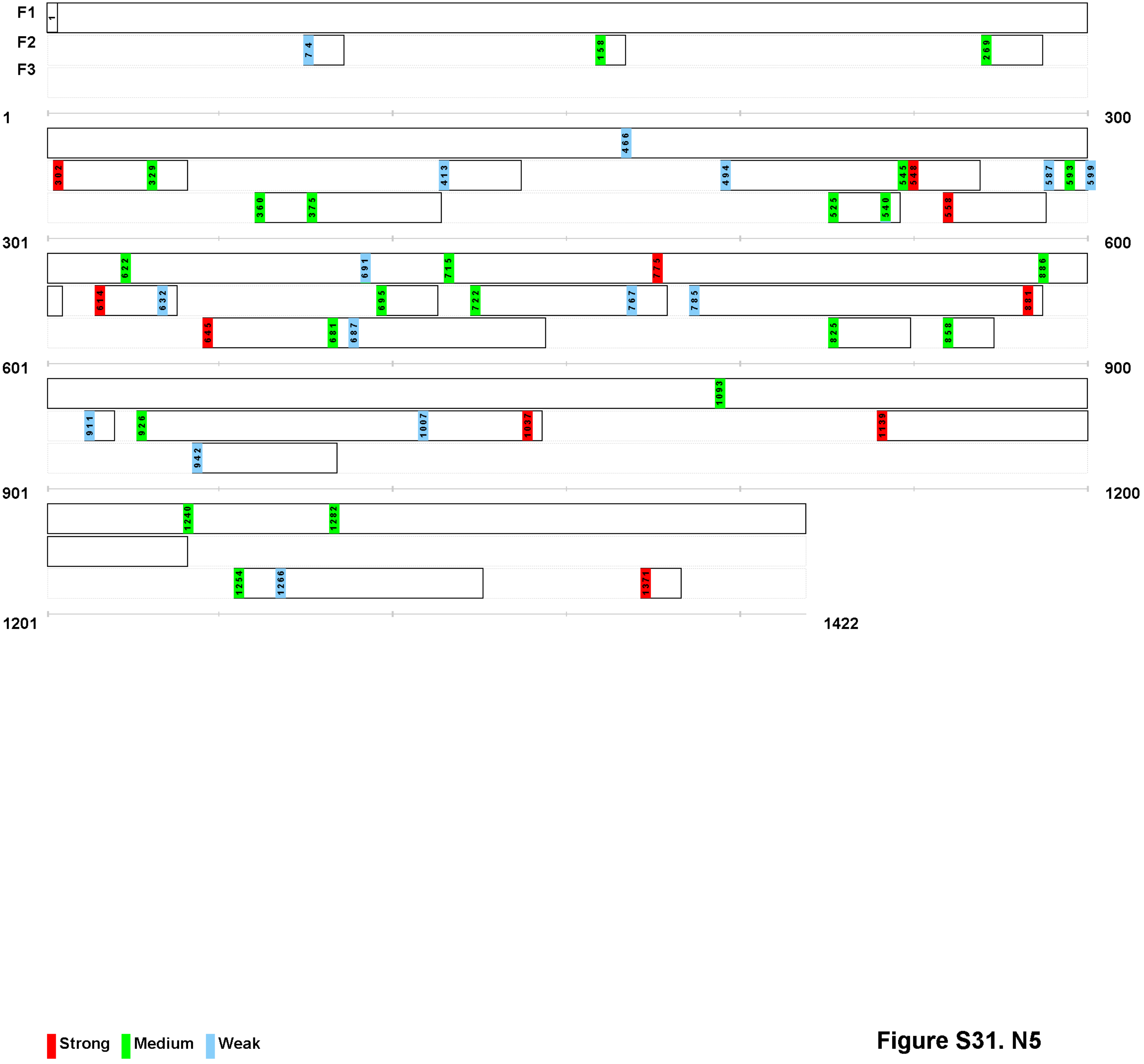

Figure S31. N5

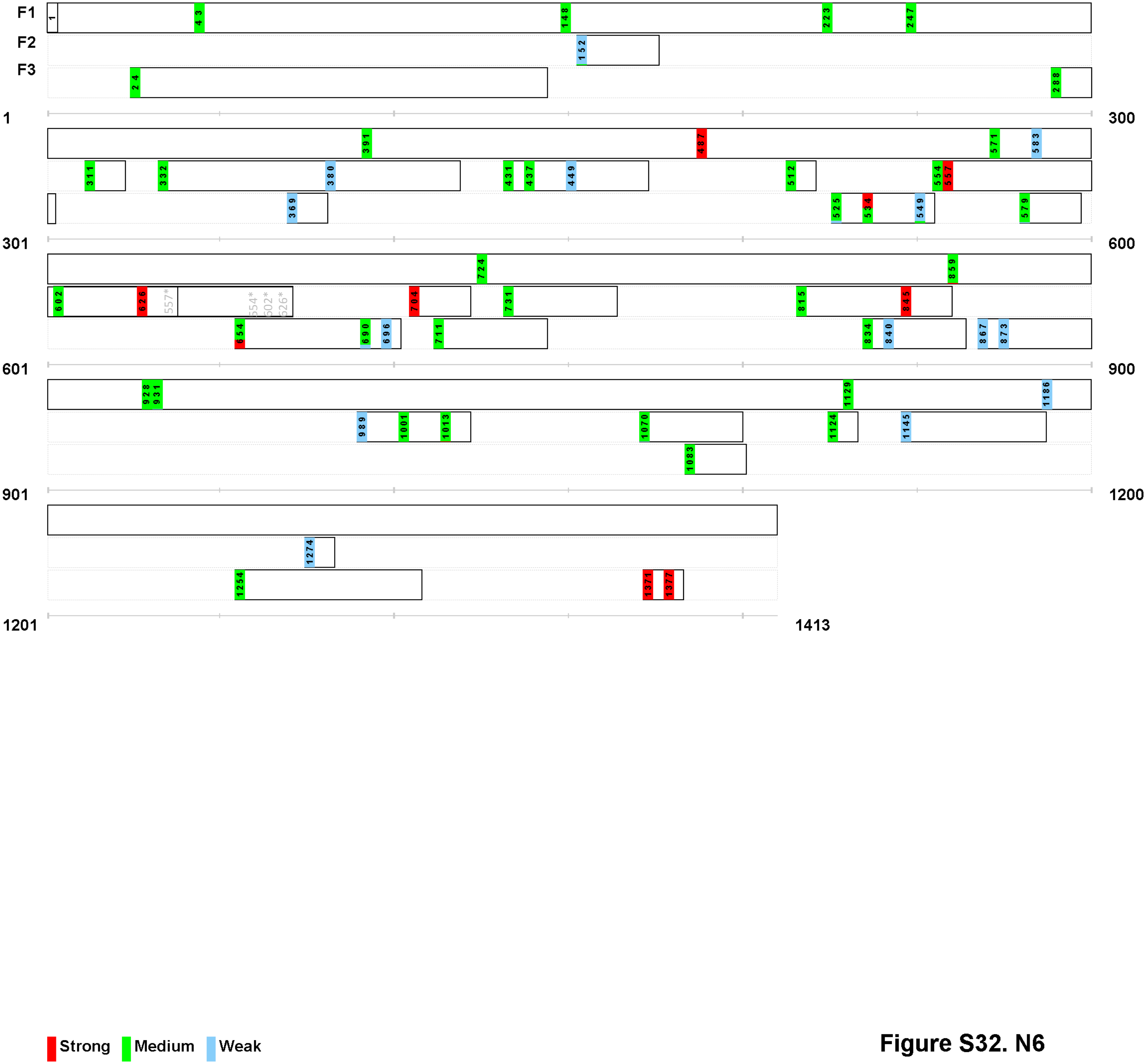

Figure S32. N6

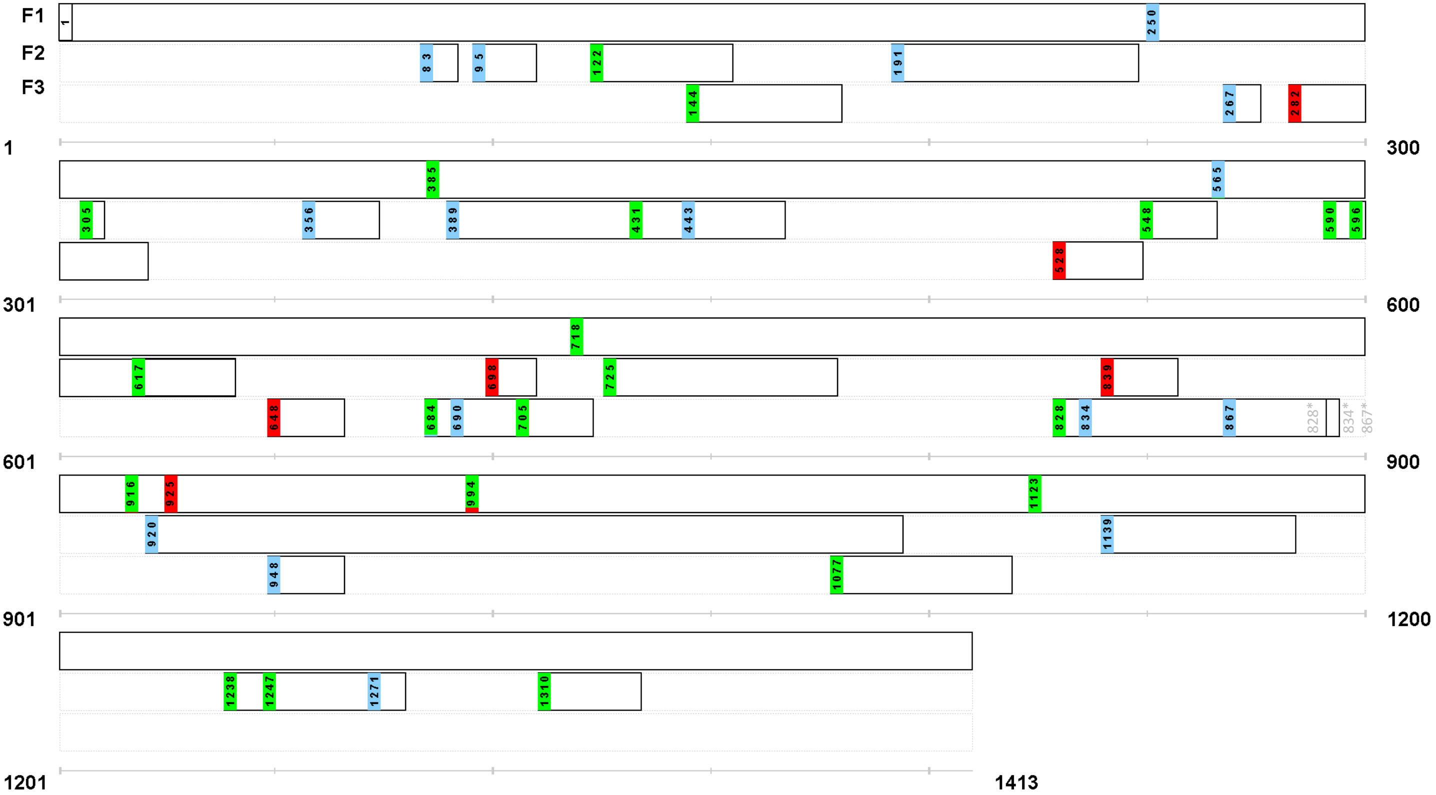

Strong Medium Weak

Figure S33. N7

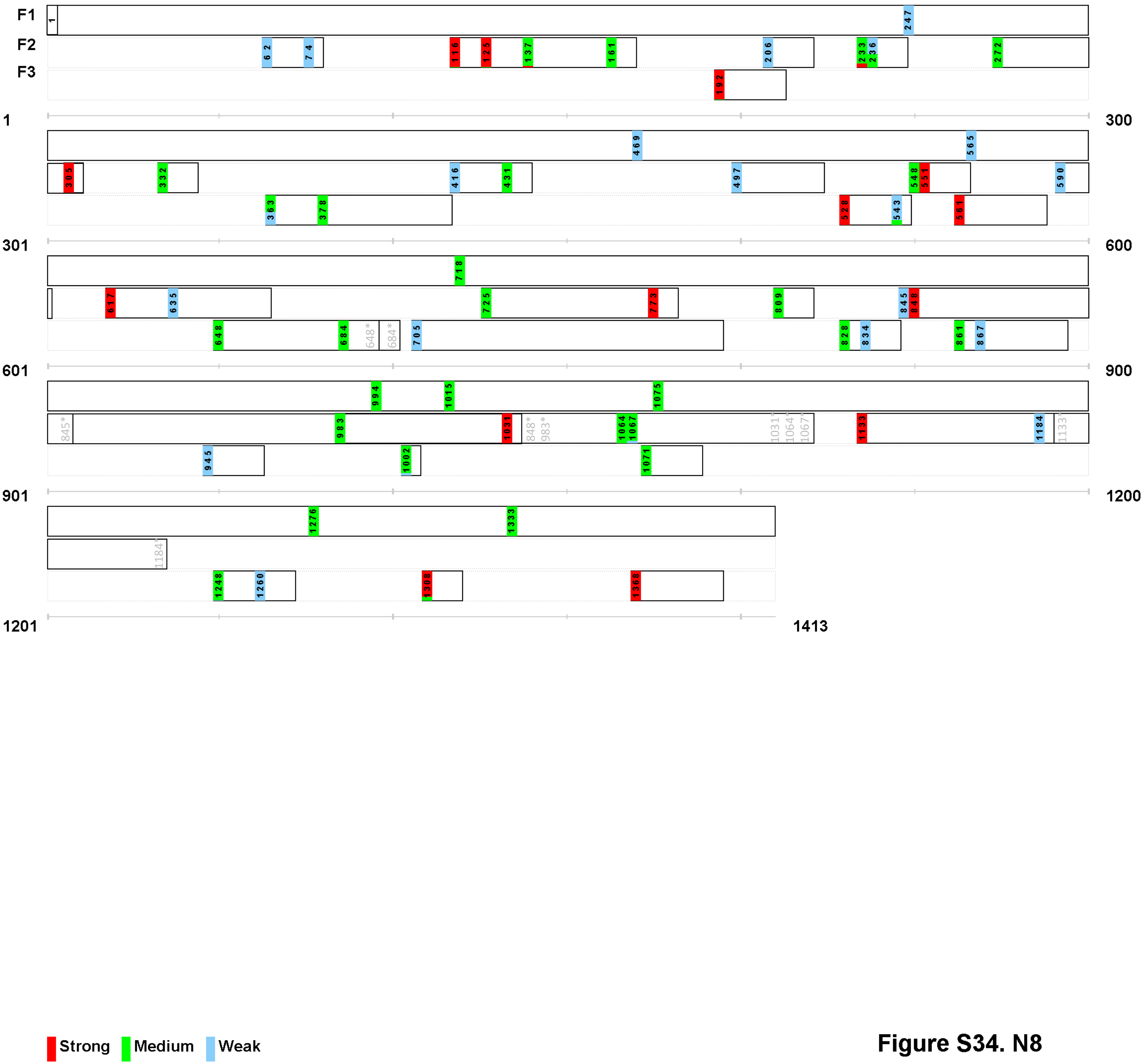

Figure S34. N8

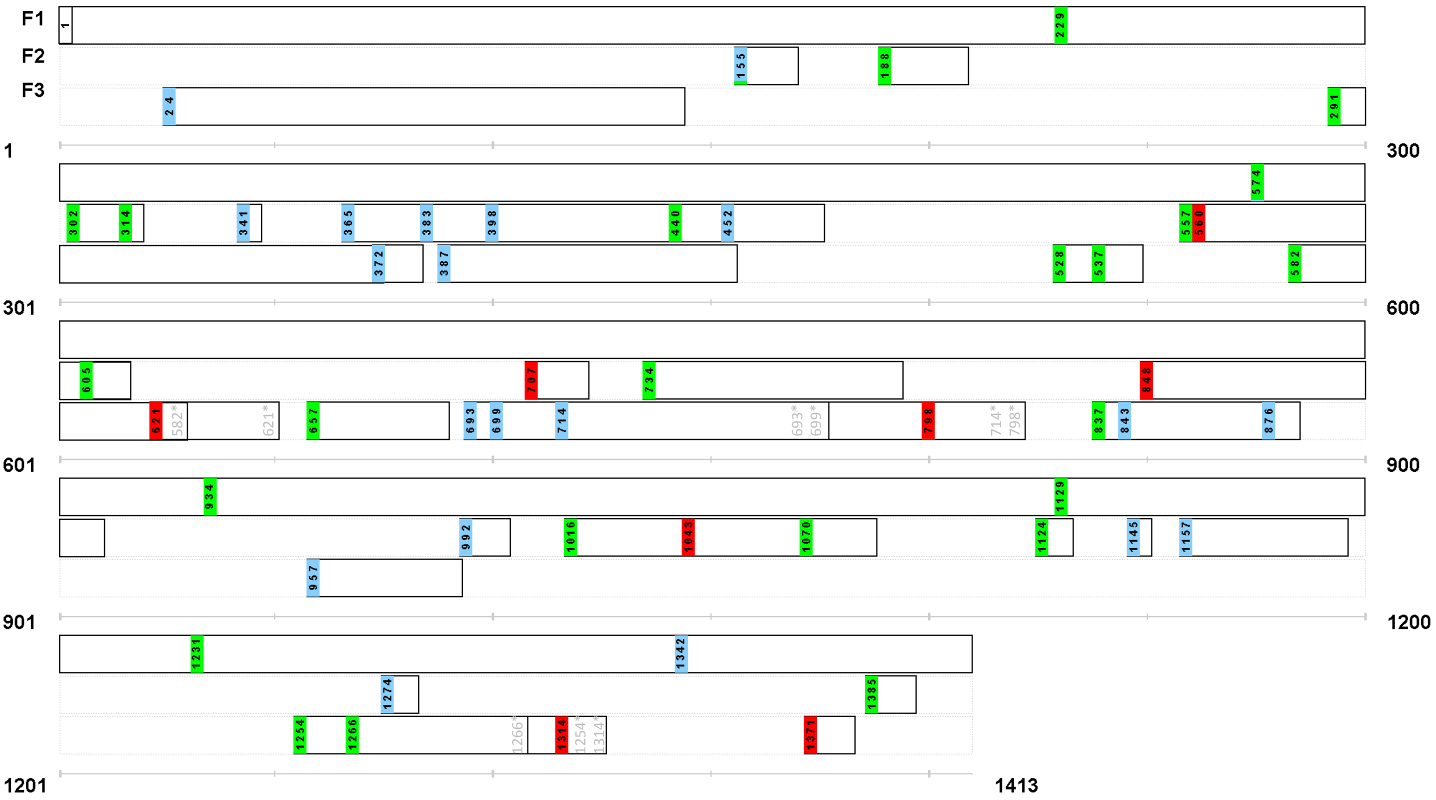

Strong Medium Weak

Figure S35. N9

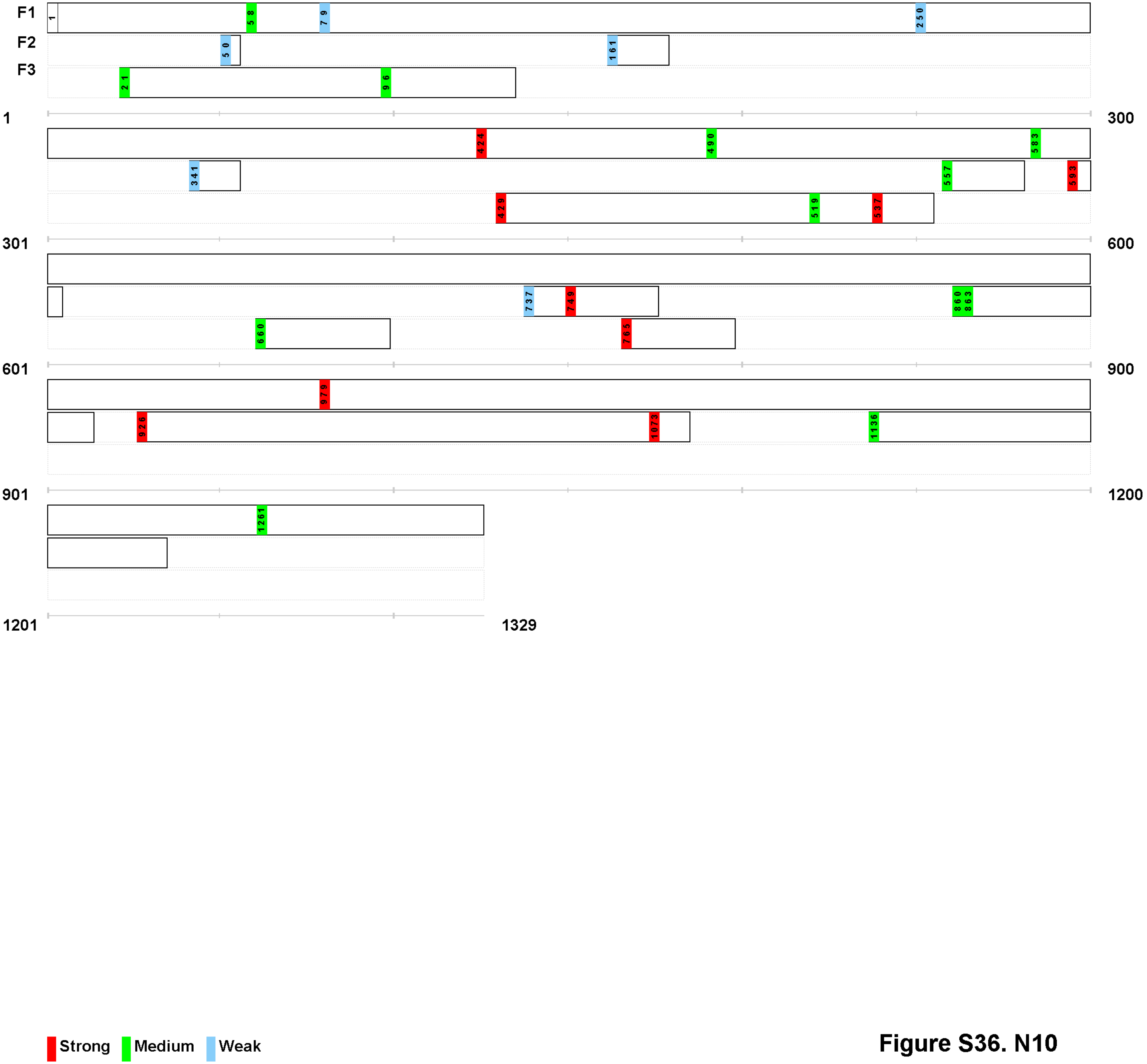

Figure S36. N10

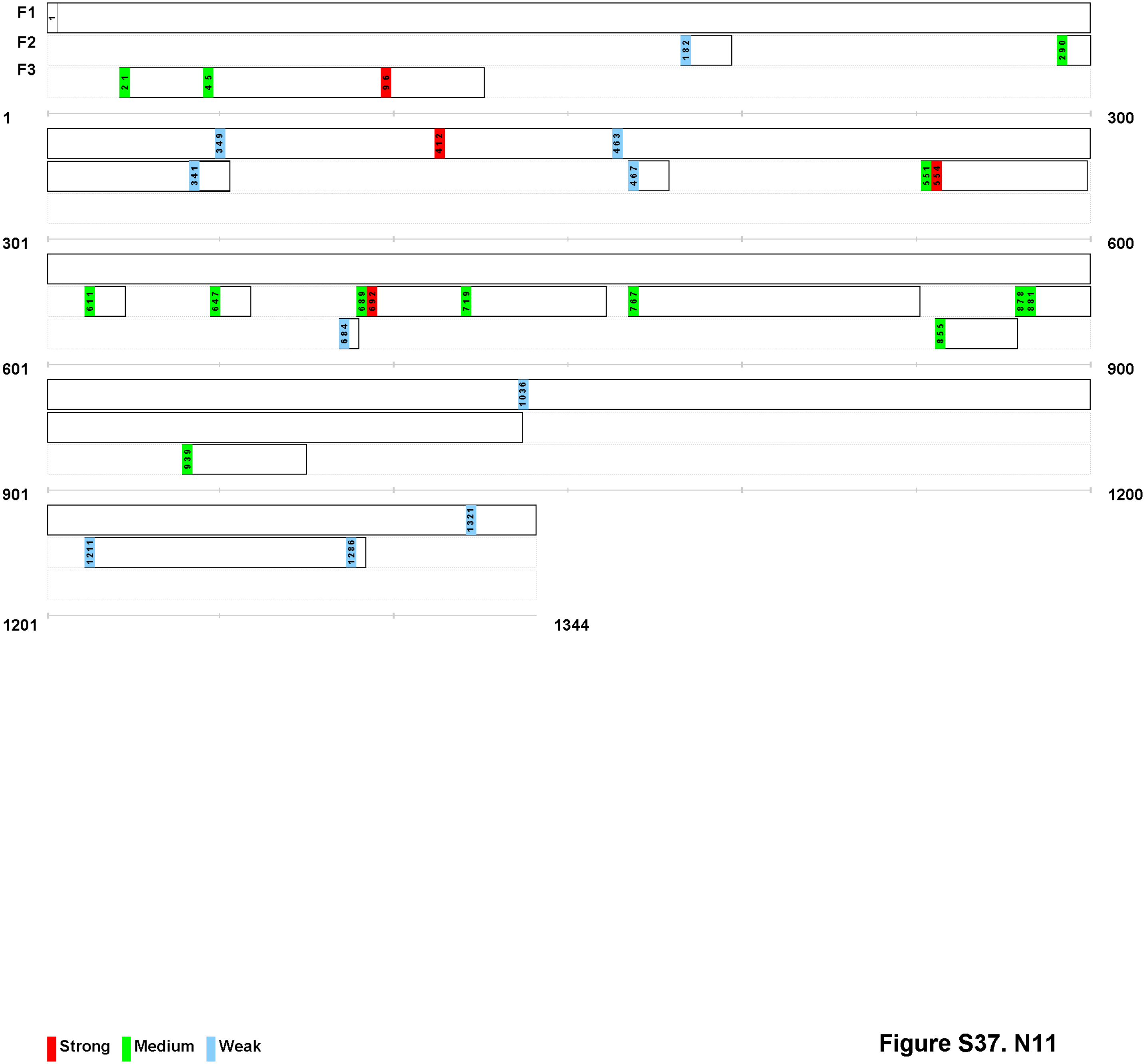

Figure S37. N11

Figures S38. Nucleotide alignments for the BLASTP reports. Four nt sequence alignments are used to illustrate the single nt insertions or deletions (red) that disturbed the reading frames, producing database hits to the ORFs (Table 3). (a) Corresponds to Case 5 in Table 3. Query = JN087010, which translates into AEI29961 in Table 3. Sbjct = HM145493, the second-closest hit in the NT database, excluding the query sequence itself. b) Corresponds to Case 6 in Table 3. Query = AY790308, which translates into AAV68025 in Table 3. Sbjct = AY862651, the second-closest hit in the NT database, excluding the query sequence itself. c) Corresponds to Case 7 in Table 3. Query = M25291, which translates into AAA43224 in Table 3. Sbjct = CY005956, the second-closest hit in the NT database, excluding the query sequence itself. d) Corresponds to Case 8 in Table 3. Query = EU429730, which translates into ACA04672 in Table 3. Sbjct = EU429760, the second-closest hit in the NT database, excluding the query sequence itself.

(a)

```
Query 1681 TGCCATAGGGGTGATACACAAATTCAAACGAGAGAGATCATTCGAGCTGAAGAAGCTGTG 1740
Sbjct 1681 TGCCATAGGGGTGATACACAAATTCAAACAAG-GAGATCATTCGAGCTGAAGAAGCTGTG 1739
...
Query 1801 TATCCGGAATCTCCACATCCCAGAGGTCTGCTTGAAGTGGGAACTAGATGGATGAAGATT 1860
Sbjct 1800 TATCCGGAATCTCCACATCCCAGAGGTCTGCTTGAAGTGGGAACTA-ATGGATGAAGATT 1858
Query 1861 ATCAGGGTAGGCTGTGTAATCCTTCTGAACCCATTTGTCAGTCATAAGGAAATTGAGTCT 1920
Sbjct 1859 ATCAGGGTAGGCTGTGTAATCCTT-TGAACCCGTTTGTCAGTCATAAGGAAATTGAGTCT 1917
```

(b)

```
Query  646 ATTGACGATCGGATTTTCTGGAGAGGTGAAAATGGACCAAAGAACAAGGGATTGCATATG 705
Sbjct  601 ATTAACGATCGGAATTTCTGGAGAGGTGAAAATGGACGAA-GAACAA-GGATTGCCTATG 658
```

(c)

```
Query 1260 AACAAGTAGAGGGAAGGATCCAAGACTTAGAGAAGTATGTTGAAGACAC-AAGATTGACT 1318
Sbjct 1260 AACAAGTAGAGGGAAGAATTCAAGACTTAGAGAAGTATGTTGAAGACACAAAGATTGACC 1319
```

(d)

```
Query  721 TGGGAAC-TGTACAGTAGTAATGACTGATGGAAAGTGCATCAGGAAGGGGCTGATACTAG 779
Sbjct  721 TGG-AACTTGTACGGTAGTAATGACTGATGG-AAGTGCATCAGGAA-GGGCTGATACTAG 777
Query  780 AATATTATTCGTTAAAGAGGGGAAAATCGTGCATATCAGCCCACTATCAGGGAAGTGCCC 839
Sbjct  778 AATATTATTCGTTAAAGAGGGGAAAATCGTGCATATCAGCCCACTATCA-GGAAGTGCCC 836
```
